# Pedigree-Based Analysis in a Multiparental Population of Octoploid Strawberry Reveals QTL Alleles Conferring Resistance to *Phytophthora cactorum*

**DOI:** 10.1534/g3.117.042119

**Published:** 2017-06-05

**Authors:** Jozer Mangandi, Sujeet Verma, Luis Osorio, Natalia A. Peres, Eric van de Weg, Vance M. Whitaker

**Affiliations:** * Department of Horticultural Science, University of Florida, IFAS Gulf Coast Research and Education Center, Wimauma, Florida 33598; † Department of Plant Pathology, University of Florida, IFAS Gulf Coast Research and Education Center, Wimauma, Florida 33598; ‡ Plant Breeding, Wageningen University and Research, 6708 PB, The Netherlands

**Keywords:** disease resistance, *FaRPc2*, FlexQTL, *Fragaria*, haplotype, quantitative trait locus, MPP, Multiparental Populations

## Abstract

Understanding the genetic architecture of traits in breeding programs can be critical for making genetic progress. Important factors include the number of loci controlling a trait, allele frequencies at those loci, and allele effects in breeding germplasm. To this end, multiparental populations offer many advantages for quantitative trait locus (QTL) analyses compared to biparental populations. These include increased power for QTL detection, the ability to sample a larger number of segregating loci and alleles, and estimation of allele effects across diverse genetic backgrounds. Here, we investigate the genetic architecture of resistance to crown rot disease caused by *Phytophthora cactorum* in strawberry (*Fragaria* × *ananassa*), using connected full-sib families from a breeding population. Clonal replicates of > 1100 seedlings from 139 full-sib families arising from 61 parents were control-inoculated during two consecutive seasons. Subgenome-specific single nucleotide polymorphism (SNP) loci were mapped in allo-octoploid strawberry (2*n* = 8 × = 56), and FlexQTL software was utilized to perform a Bayesian, pedigree-based QTL analysis. A major locus on linkage group (LG) 7D, which we name *FaRPc2*, accounts for most of the genetic variation for resistance. Four predominant SNP haplotypes were detected in the *FaRPc2* region, two of which are strongly associated with two different levels of resistance, suggesting the presence of multiple resistance alleles. The phenotypic effects of *FaRPc2* alleles across trials and across numerous genetic backgrounds make this locus a highly desirable target for genetic improvement of resistance in cultivated strawberry.

The use of multiple connected families to analyze QTL in crop species has risen dramatically in recent years (Sneller *et al.* 2009). Multiparental populations have many advantages for QTL analysis compared to biparental populations. Single F_1_ or F_2_ crosses sample only the alleles present in two parents, describing only a small portion of the genetic variability present within breeding programs (Würschum 2012). In contrast, complex populations facilitate the sampling of many allele combinations and estimation of QTL effects across a wide array of representative genetic backgrounds. Such populations also allow increased recombination and mapping resolution, as well as increased power to detect loci with small phenotypic effects (Ogut *et al.* 2015).

For inbred crops, multiparental designs such as Multiparent Advanced Generation Intercross (MAGIC) are quite efficient for analyzing complex traits in breeding-relevant germplasm (Cavanagh *et al.* 2008). In clonally propagated outbreeding crops, such as cultivated strawberry (*Fragaria* × *ananassa*), breeding germplasm is usually structured as many pedigree-connected full-sib families. Thus, for outbreeding crops, simultaneous QTL detection in many connected families is ideal, allowing the same breeding germplasm to be used for both QTL analysis and selection of high-performing clones. In such an approach, existing families, phenotypic data, and pedigree information from breeding programs are efficiently utilized (Tisné *et al.* 2017). However, breeding populations typically have many families that differ in size and relatedness and span many generations. Furthermore, each full-sib family may segregate for up to four alleles at a given locus. Analyses of complex pedigree structures have been limited in the past by a lack of appropriate statistical methods and software. To address this challenge, Bayesian analysis methods originally developed for the analysis of human and cattle genetic data have been adapted and applied for genome-wide multiple QTL mapping in crops (Bink *et al.* 2002). Using FlexQTL software, pedigree-based QTL analyses can now be efficiently implemented. Bink *et al.* (2014) demonstrated the power of this software in an apple dataset of > 1000 individuals from 27 full-sib families, detecting multiple QTL for fruit firmness, estimating QTL effects, and assigning QTL genotypes to pedigree-related individuals.

The development of molecular tools for cultivated strawberry has been hindered in the past by its allo-octoploid (2*n* = 8 × = 56) genetic constitution, limiting the development of dense linkage maps and high-throughput marker resources (Sargent *et al.* 2009). For this reason, only a few studies have reported major genes and QTL in cultivated strawberry to date (Van de Weg 1997a,b; Haymes *et al.* 1997, 2000; Weebadde *et al.* 2008; Zorrilla-Fontanesi *et al.* 2011, 2012; Lerceteau-Köhler *et al.* 2012; Antanaviciute *et al.* 2015; Sooriyapathirana *et al.* 2015; Roach *et al.* 2016). However, the sequencing of the diploid woodland strawberry (*F. vesca*) genome (Shulaev *et al.* 2011) set the stage for the development of the Affymetrix IStraw90 Axiom array, a 90 K SNP array for octoploid strawberry (Bassil *et al.* 2015). Various ploidy reduction strategies were employed in the design of the array, resulting in a high proportion of subgenome-specific SNP markers. The array has been revolutionary in strawberry genetics, allowing the marker throughput and marker call consistency necessary for large-scale marker-trait association studies.

Phytophthora crown rot (PhCR) of strawberry is a disease of commercial importance worldwide, particularly in annual production systems in California and Florida after the loss of methyl bromide soil fumigation. *Phytophthora cactorum* was first reported as the cause of crown rot of strawberry in Germany in 1952 (Deutschmann 1954) and in North America in 1989 (Wilcox 1989). Oospores survive in the soil or in infected plants and, under optimal conditions, germinate and produce sporangia from which zoospores are released. Zoospores are motile and can disperse through water, entering root and crown tissues through wounds. After infection, young leaves turn bluish-green and wilt, followed by entire plant collapse and death. Dissection of crowns reveals brown discoloration and disintegration of vascular tissues (Maas 1998). While some fungicides are labeled for control of PhCR in strawberry, they are prone to the development of resistance by the pathogen (Jeffers *et al.* 2004). Thus, host resistance is a desirable strategy for management of this disease.

Screening for genetic resistance to PhCR via controlled inoculations is expensive, labor-intensive, and time-consuming, and resources for marker-assisted breeding would be highly desirable. Identifying and introgressing new sources of resistance to *P. cactorum* from wild relatives may be possible (Harrison *et al.* 1996, 1997). However, extensive backcrossing and linkage drag would be major drawbacks to this approach. Utilizing genetic variation for PhCR resistance already available in elite germplasm should be more feasible and desirable, given that strong resistance has been observed in the University of Florida (UF) breeding population and in other breeding programs (Eikemo *et al.* 2003; Shaw *et al.* 2006, 2008). However, very little is known about the genetic architecture of resistance to PhCR. The inheritance of resistance to PhCR in strawberry has been the subject of only a few studies (Eikemo *et al.* 2003; Shaw *et al.* 2006, 2008; Davik *et al.* 2015), and the number and location of loci involved in cultivated strawberry is not known.

The strawberry breeding program at UF utilizes a phenotypic recurrent selection system that has resulted in the release of 14 cultivars since 1952 (Whitaker *et al.* 2011, 2012, 2015). Improvements in quality traits such as fruit size, yield uniformity, and cull rates have been evident when comparing cultivars from different eras (Whitaker *et al.* 2011). However, regular screening for PhCR has been conducted at UF only in the last 5 yr. The elite strawberry breeding population evaluated at UF in a given year consists of ∼10,000 seedlings generated through 75–125 biparental crosses. For this population, a multifamily QTL mapping approach would be highly appropriate, allowing the detection of multiple alleles and their effects in many genetic backgrounds representative of the breeding germplasm.

The specific objectives of the present study were to: (1) identify QTL for resistance to PhCR via pedigree-based analysis in a complex, multiparental breeding population, (2) estimate QTL effects across breeding germplasm, (3) develop an understanding of QTL allelic variation and allele effects, and (4) validate QTL presence and effects in advanced breeding selections and cultivars representing the parent pool of the breeding program.

## Materials and Methods

### QTL discovery populations

**Figure 1:**
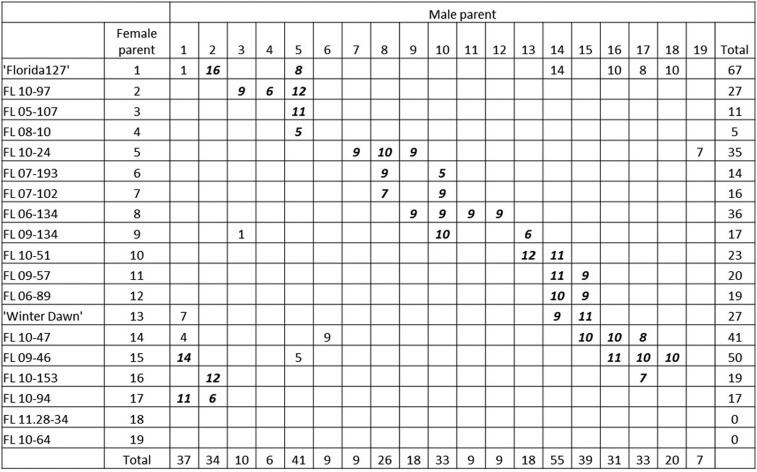
Schematic representation of an incomplete, circular diallel mating design with additional biparental crosses from the strawberry elite breeding population at University of Florida evaluated during the 2013–2014 season. The number shown at the intersection of each pair of parents represents the number of seedling individuals evaluated for the respective full-sib family. Circular diallel crosses are in bold and additional crosses are in plain text. The total number of seedlings arising from each parent used as a female and as a male are shown in the far right column and bottom row, respectively.

Two multiparental population sets were evaluated in two consecutive strawberry production seasons, 2013–2014 and 2014–2015. Each consisted of a representative subset of crosses made in the UF breeding program in each year. All parents were highly related, as they all arose from an elite breeding population that had undergone phenotypic recurrent selection for > 15 generations. The 2013–2014 and 2014–2015 population sets consisted of 67 and 75 full-sib (Table 1) families respectively, each from crosses made among 35 parents. Most of the crosses (40 in 2013–2014 and 37 in 2014–2015) were made following an incomplete circular diallel design to achieve maximum connectedness among the full-sib families, and the remaining biparental crosses were made among other parents of interest in the breeding program (Figure 1, Table 1, Supplemental Material, Figure S1 and Figure S2). Nine parents were common to both years, but specific cross combinations (full-sib families) were not replicated across years. Seeds from each cross were germinated at the UF Gulf Coast Research and Education Center (GCREC) in Wimauma, Florida in April of each year, transplanted into individual peat pellets and transported to a summer nursery located near Monte Vista, Colorado in June of each year for clonal replication. Runner plants (clonal replicates) from each seedling were allowed to root, then collected from the nursery as bare-root transplants in September and transported to Florida for evaluations at the GCREC research farm.

**Table 1:** Summary statistics of QTL discovery populations and validation sets from the University of Florida strawberry breeding program evaluated for resistance to crown rot caused by *Phytophthora cactorum* during the 2013–2014 and 2014–2015 seasons

	Discovery		Validation	
	2013–2014	2014–2015	2013–2014	2014–2015
Parents	35	35	128	78
Full-sib families	67	72	132	92
Individuals/family	1–16	1–11	1–15	1–24
Total individuals	551	580	281	343
Clones*^a^*/individual	3	3	4–20	6–20
Total clones	1922	1793	5133	6351

a Clones refer to runner plants obtained from the same individual.

From each full-sib family, a maximum of 16 and 11 seedlings (called individuals henceforth) were selected randomly in 2013 and 2014, respectively. Three clonal replicates were collected from each individual in the summer nursery and were planted in a randomized complete block field design with one runner plant in each of three blocks, each block consisting of two planting beds. Transplants were inoculated at the time of planting on 10 Oct 2013 and 9 Oct 2014.

### QTL validation sets

Two sets of individuals consisting of previous season selections, elite selections, and commercial cultivars were evaluated for resistance to PhCR during the same two seasons but in separate control-inoculated trials (Table 1). The set evaluated in 2013–2014 consisted of 281 individuals (253 selections, 23 elite selections, and 5 commercial cultivars), and the set evaluated in the 2014–2015 season consisted of 343 individuals (237 selections, 98 elite selections, and eight commercial cultivars). Sixty individuals were common across the two validation sets.

For each selection or cultivar, two plots containing between 2 and 10 plants (nine plants per plot or 18 plants total on average in each year) were planted in two blocks in a randomized complete block (RCB) field design with each block consisting of six planting beds in the 2013–2014 season and eight planting beds in the 2014–2015 season. The transplants were inoculated at the time of planting on 10 Oct and 14 Oct 2013 and 15 Oct and 16 Oct 2014. The QTL discovery population set and the validation set in a given year were planted nearby each other in the same field but in separate trials.

### Inoculation and disease rating

*P. cactorum* inoculum was produced by transferring 5 mm plugs of actively growing colonies on potato dextrose agar to sterile, empty petri dishes and then flooding the plugs with V8 juice broth up to the surface of the plugs. Plates were incubated at room temperature in the dark for 5 d allowing the isolates to grow and form a mat over the broth. The broth was then discarded and the mats were washed twice with sterile-deionized water. The mats were flooded with sterile pond water and incubated at room temperature under constant light between 24 and 48 hr, until sporangia were visible under the dissecting scope. The mycelial mats were flooded with fresh, cold sterile-deionized water (at 4°) and the plates chilled in the refrigerator for 15–30 min, followed by 1 hr at room temperature to release zoospores. Motile zoospores were collected by filtering the suspension through a double layer of cheese cloth. Inoculum was produced for three to four isolates separately following this protocol. The concentration of motile zoospores for each isolate was determined by taking 500 µl aliquots and vortexing for 2 min to dislodge flagella before counting. The concentrations of the zoospore suspensions were adjusted for each isolate and then combined. The isolates were obtained from crowns of symptomatic strawberry plants of different cultivars submitted to the GCREC plant disease diagnostic clinic. The samples came from different strawberry farms located in the production area of central Florida. Isolates were obtained from single branched hyphae and were stored at room temperature in sterile water for up to 4 yr. All isolates were cycled through susceptible genotypes several weeks prior to inoculation to achieve maximum virulence.

The discovery population evaluated during the 2013–2014 season was inoculated with isolates 10–115, 12–419, and 12–420, combined in a zoospore suspension with a final concentration of 1 × 10^4^ zoospores/ml. Due to low zoospore production, the validation set in this season was inoculated only with isolates 10–115 and 12–419 using a zoospore suspension at 1 × 10^4^ zoospores/ml for the replication planted on 10 Oct 2013, and the same two isolates with a suspension of oogonia, chlamydospores, and sporangia at 7 × 10^3^ mixed structures/ml for the replication planted on 14 Oct 2013. For the 2014–2015 season, isolates 12–420, 12–417, 09–101, and 10–115 were combined at 1 × 10^4^ zoospores/ml for both the discovery population and validation set. To our knowledge, there is no evidence for the presence of races or pathotypes of *P. cactorum*. Inoculations were performed by immersing the roots of 10 transplants for 5 sec in 500 ml of the zoospore suspension up to the base of the crown immediately before planting. Used inoculum was replaced continuously and ∼4 L of inoculum were used per planting bed. Overhead irrigation was applied during daylight hours for 10 d for plant establishment and to ensure wet conditions for infection. Susceptibility to *P. cactorum* was recorded weekly for a period of up to 20 wk starting 3 wk after transplanting. Plant collapse was recorded when 75% or more of the total leaf canopy, considering leaves from all developed crowns, of each plant had wilted and collapsed onto the bed surface. Noninoculated controls of multiple susceptible genotypes were included in both seasons. Reisolations from crowns of randomly chosen collapsed plants were performed every 2 or 3 wk in both seasons to confirm *P. cactorum* infection.

### Production trait measurements

Potential relationships between resistance and other vegetative and fruit traits commonly measured in the breeding program were explored by evaluating noninoculated field trials of the validation sets. Such traits could possibly influence susceptibility responses or be negatively correlated with resistance, negatively impacting resistance breeding. The traits were soluble solids content (SSC), total marketable yield (TMY, in grams), total marketable fruit number (TMF), total fruit number (TFN, marketable and unmarketable), total number of unmarketable fruit or total culls (TC), and average fruit weight (AWT, in grams). Five clonal replicates of each individual were planted adjacent to the inoculated plots on 7 Oct 2013 and 15 Oct 2014. Single plant plots were planted in a RCB design in five beds, each bed serving as a block. SSC was evaluated as the average of five measurements performed between 10 Dec 2013 to 4 Mar 2014 and 16 Dec 2014 to 10 Mar 2015. From each plant, one fully ripe fruit was squeezed over a hand-held refractometer (PAL-1; Atago Co., Tokyo, Japan) at each evaluation. Five additional fruit production traits were evaluated from weekly harvests from 19 Nov 2013 to 11 Mar 2014 and 24 Nov 2014 to 3 Mar 2015. For each harvest, marketable fruit were separated from unmarketable fruit and both categories counted, but only marketable fruit were weighed.

### DNA isolation and genotyping

For the discovery population sets and validation sets in both years, 30–60 mg of unexpanded leaf tissue from each individual were collected into 96 well plates and frozen at −80° until extraction. DNA extraction was performed using the E-Z 96 Plant DNA Kit (Omega Bio-Tek, Norcross, GA) with only minor modifications for the 2013–2014 samples and the same kit or a modified CTAB method (Chang *et al.* 1993) for the 2014–2015 samples. Prior to DNA extraction, frozen samples were ground with a Fisher Scientific PowerGen high-throughput homogenizer (Pittsburgh, PA) twice for 2 min with a 30 min refreezing at −80° between grindings.

From the discovery population sets, 551 (Table 1) individuals were genotyped in the 2013–2014 season and 580 in the 2014–2015 season. Immediate parents and other pedigree-connected individuals were also included. From the validation sets, 245 individuals were submitted for genotyping each year. Genotyping was performed using the Affymetrix IStraw90 Axiom Array (Bassil *et al.* 2015). Genotyping errors were detected by comparing, within full-sib families, each SNP diplotype of each individual with that of the parental genotypes and replaced with “no call” if incorrect. Similarly, when SNP diplotypes of the parents were not consistent with their progeny, the correct parent was inferred and was corrected accordingly when possible using a Microsoft Excel-based tool. If the correct parent was not obvious, the incorrect parent was replaced with a virtual “undetermined” parent. In addition, markers with inheritance errors in LG 7D, as determined in the “mconsistency” file of FlexQTL outputs, were removed from the data and the data were analyzed in FlexQTL until no errors were observed.

### Linkage mapping

In order to assign SNP markers to the four subgenomes of octoploid strawberry and order them in a genetically meaningful way, we relied on a previously developed map (E. van de Weg, unpublished data). This map was constructed from 75 progeny of “Holiday” × “Korona” using JOINMAP 4.1 software (Van Ooijen 2006, 2011), and at the time contained 12,966 SNP markers spanning 21.0 Morgans divided over 28 LGs (version 20150310, H. J. J. Koehorst-van Putten and E. Van de Weg, unpublished data) which were ordered and named according to the SSR reference map of Van Dijk *et al.* (2014). Nine of the 28 LGs were represented by two separate subgroups for the proximal and distal part of a homologous chromosome pair, as large homozygous regions in both parents (consistently present in the species) prevented their merging (Van Dijk *et al.* 2014). As a result, ∼11% (250 cM) of the genome was not represented in the “Holiday” × “Korona” linkage map (H. J. J. Koehorst-van Putten and E. Van de Weg, unpublished data).

In view of computation time, we further decreased marker density by eliminating markers at identical map positions with lowest minor allele frequencies (MAF), resulting in a set of 5100 makers that were informative for both the 2013–2014 and 2014–2015 discovery population sets. These markers were further curated for consistent performance across the germplasm, eliminating those that showed conflicting calls based on true pedigree relationships. Fraction values of observed (oDR) and expected (eDR) double recombinant singletons were obtained from FlexQTL software and markers with oDR minus eDR > 0.05 were also excluded from the map (Clark *et al.* 2017). The small proportion of markers with MAF below 0.1 was retained to capture unique map positions. The map was further curated using graphical genotyping, following the procedure of Bassil *et al.* (2015) for LG that showed a QTL. Spurious singleton calls identified by JOINMAP’s genotype probability module that could not be resolved by manual reordering of markers were made missing, and graphical genotyping-derived marker orders were used.

### QTL analysis

Weekly plant collapse evaluations from the discovery populations were utilized to calculate the area under the disease progress curve (AUDPC, Madden and Campbell 1990) for each individual for 28 wk in both seasons. In addition to the progeny, marker data for pedigree-connected cultivars and selections up to two generations previous to the direct parents of the individuals in the discovery population sets were included in the analysis (Figure S1 and Figure S2).

The QTL analysis was performed using a Markov chain Monte Carlo (MCMC)-based Bayesian analysis in FlexQTL, using a model with additive QTL effects and a maximum number of QTL of 15. Prior number of QTL was set to 1 or 3 (Bink *et al.* 2002, 2014) and genome-wide analyses were performed twice for each prior. Each analysis was performed with different starting seeds to create independence between iterations, using simulation chain lengths of 100,000 iterations with thinning values of 100. The effective sample size in the parameter file was set to 101 to ensure convergence with effective chain size of at least 101. Each of the two iterations converged (effective chain samples, or ECS, ≥ 100 for each of the parameters mean, variance of the error, number of QTL, and the variance for the number of QTL) as recommended by Bink *et al.* (2014). Full FlexQTL parameters and values chosen for the present analyses are provided in Table S1.

Two times the natural log of Bayes factors (BF) generated from genome-wide FlexQTL analysis were used to determine the total number of QTL (2lnBF_10_ ≥ 5) as well as QTL positions on individual LGs (Kass and Raftery 1995; Rosyara *et al.* 2013; Bink *et al.* 2014; Guan *et al.* 2015). Once the genome-wide total number of QTL was established, further analyses of individual LGs containing QTL with strong evidence (2lnBF_10_ ≥ 5) was conducted, including only those present in at least two of the four runs. Individual LGs were analyzed to examine whether the phenotypic variation explained would be similar to the genome-wide analysis. Analyses were performed in triplicate with simulation chain lengths of 10,000 iterations and thinning values of 10, and other parameters detailed in Table S1. Narrow-sense heritability (*h*^2^) was calculated by using statistical inferences from FlexQTL software outputs with the formula

$$h^{2}=\frac{\text{VP}-\text{VE}}{\text{VP}}$$

where VP is the phenotypic variance of the trait, and VE is the residual error variance (Bink *et al.* 2014; FlexQTL output). The proportion of phenotypic variation explained (PVE) by a particular QTL was calculated using the formula

$$\text{PVE}=\left(\frac{\text{wAVt}}{\text{VP}}\right)\times 100$$

where wAVt is weighted additive variance of the trait, adjusted for the portion of the variance explained by the QTL on a particular chromosomal position (obtained after PostQTL analysis), and VP is the total phenotypic variance of the trait.

To examine recombination patterns in the tested germplasm in and around identified QTL regions, pairwise linkage disequilibrium (LD) ($r^{2}$) among SNP markers was determined with Haploview software using the four-gamete method (Barrett *et al.* 2005). Marker haploblocks and haplotypes for the identified QTL regions were constructed from FlexQTL outputs. Markers were selected within the QTL intervals defined by FlexQTL and according to LD values between markers. The same set of markers was utilized to determine haplotypes in both the discovery population sets and validation sets. The “mhaplotype” files of the QTL discovery populations were examined first since the presence of multiple individuals within full-sib families and the circular diallel crossing design allowed determinations of the consistency of SNP haplotypes.

### Haplotype effects

For the QTL region, incomplete marker information of parents was imputed manually by comparing the SNP haplotypes of individuals from all the full-sib families in which the parent was involved and matching to the most probable SNP haplotype that included the markers already present in the parent. Incomplete marker information of individuals was imputed manually based on the haplotype segregation from the respective parents. Unique haplotypes were named ^1^H to Hn according to the proportion of total haplotypes, from higher to lower proportion.

A Kruskall–Wallis nonparametric test was utilized to determine the statistical significance of diplotype effects using Proc NPAR1WAY in SAS (version 9.3; SAS Institute Inc., Cary, NC). Pair-wise comparisons for the diplotype effects were determined with the Steele–Dwass nonparametric multiple comparison test and were considered significant at *P* < 0.05. Diplotype effects were compared, and haplotype effects were inferred from combinations of diplotypes. For example, the relative effects of haplotypes H2 and H3 can be discerned by comparing the ^1^H|H2 and ^1^H|H3 diplotype effects. To further investigate the phenotypic variation explained by the *FaRPc2* locus, haplotypes were assigned based on the direction of their effects to two putative QTL allele categories: *FaRPc2* and *farpc2*. *FaRPc2* alleles included haplotypes with negative AUDPC effects (lower mortality) in both discovery populations. Haplotypes with positive (higher mortality) AUDPC effects were assigned to the *farpc2* allele. Genotypes were assigned to each individual based on its SNP haplotypes as *FaRPc2*|*FaRPc2*, *FaRPc2*|*farpc2*, and *farpc2*|*farpc2*. The mean effects of allele combinations were evaluated with the same statistical procedure as for the diplotypes.

The PVE by putative allele combinations was estimated by fitting the AUDPC values to a normal distribution using Proc GLM in SAS. Allele combination effects were considered as fixed. In single-factor analysis of variance, the coefficient of determination (*R*^2^) can be used as a measure of the PVE. GLM groupings were compared with those obtained from the NPAR1WAY procedure to assess the adequacy of *R*^2^ estimation.

### QTL validation

AUDPC values for the individuals in the validation sets were calculated for each plot at the last evaluation date for analysis. SNP haplotypes from both validation sets were determined and labeled using the same methods as for the discovery populations, assigning matching SNP haplotypes with the same Hn designation. Diplotype and putative QTL allele combination effects were analyzed by the same procedures as for the discovery population sets. Significant diplotype and QTL allele effects in the validation sets would validate the presence and effects of QTL detected in the discovery population sets.

Putative QTL allele combinations were assigned to the individuals evaluated in the noninoculated plots from which production traits were measured. The effect of PhCR QTL allele combinations on production traits were evaluated using Proc GLM. The effect of blocks was included as fixed, and the allele combination and trait interaction was tested. Differences among putative allele combinations for the least square means of each production trait were determined using orthogonal contrasts at *P* < 0.05.

### Genetic parameter estimates

Variance component outputs of FlexQTL for discovery populations did not directly account for variation among clonal replicates, as FlexQTL considers just a single AUDPC value per individual calculated from the mortality of all clonal replicates over time. Thus, variance components taking clonal replication into account were obtained with ASReml software (Gilmour *et al.* 2009) for comparison. Disease incidence was utilized on the binary scale, assigning “1” to collapsed plants and “0” to surviving plants at the end of the disease rating period. Univariate analyses for the binomial scale were performed using general linear mixed models. Variance components were estimated by fitting the probability of mortality for individual plants (π) using a logit link function, η = [π / (1 − π)] which relates to the binary observations as follows:

$$y=\eta +e=X\beta +Z_{1}d+Z_{2}p+Z_{3}a+Z_{4}f+Z_{5}c+e\;$$

where y  is the vector of observations, β  is the vector of fixed effects (*i.e.*, mean, replications), X is the design matrix relating the fixed effects to the observations in y, d is the vector of beds within replication effects, p is the vector of plot within bed effects, a is a vector of random parental effects, f is a vector of random family effects, c is a vector of random clone within family effects, and e is the vector of random residuals effects. $Z_{1}$ to $Z_{5}$ are known design matrices relating the observations in y to effects in d,p,a,f, and c, respectively. The vectors, design matrices, and random components are according to the clonal test in Osorio *et al.* (2014), with the exception that a term for the effect of plots within beds $\left(Z_{2}p\right)$ was included in our analyses to account for the variation along the beds. Univariate analyses for the production traits were analyzed with the same linear model except that the model was fitted to the observed continuous data and included the initial weight of runner plants as a fixed covariate effect. Broad-sense heritability (*H*^2^) and narrow-sense heritability (*h*^2^) for each variable were estimated as follows:

$$H^{2}=\frac{\sigma_{G}^{2}}{2\sigma_{\mathrm{gca}}^{2}+\sigma_{\mathrm{sca}}^{2}+\sigma_{c\left(f\right)}^{2}+\sigma_{e}^{2}}\;\;\;$$

$$h^{2}=\frac{4\sigma_{\mathrm{gca}}^{2}}{2\sigma_{\mathrm{gca}}^{2}+\sigma_{\mathrm{sca}}^{2}+\sigma_{c\left(f\right)}^{2}+\sigma_{e}^{2}}$$

The variance components, $\sigma_{G}^{2},\sigma_{\mathrm{gca}}^{2},\sigma_{\mathrm{sca}}^{2},\sigma_{c\left(f\right)}^{2}$, and $\sigma_{e}^{2}$ were derived similarly to the methods of Mullin and Park (1992). The error variance $\left(\sigma_{e}^{2}\right)$ of the estimates for plant collapse was fixed to a value of 3.29 (Gilmour *et al.* 2009).

### Data availability

Marker genotypes, phenotypes, and pedigrees are available in supplemental data files. File S8, “Whitaker_pedigree_data policy documentation” contains detailed descriptions of File S1, File S2, File S3, File S4, File S5, and File S6. File S1 contains genotypic data for the 2013–2014 QTL discovery population set. File S2 contains genotypic data for the 2013–2014 validation set. File S3 contains genotypic data for the 2014–2015 QTL discovery population set. File S4 contains genotypic data for the 2014–2015 validation set. File S5 contains phenotypic (AUDPC) data, pedigree information (immediate parents), and assigned diplotypes for all germplasm tested. File S6 contains physical locations of SNP probes genome-wide and genetic locations of SNP markers for LG 7D. File S7 is a zip folder containing results from software outputs.

## Results

**Figure 2:**
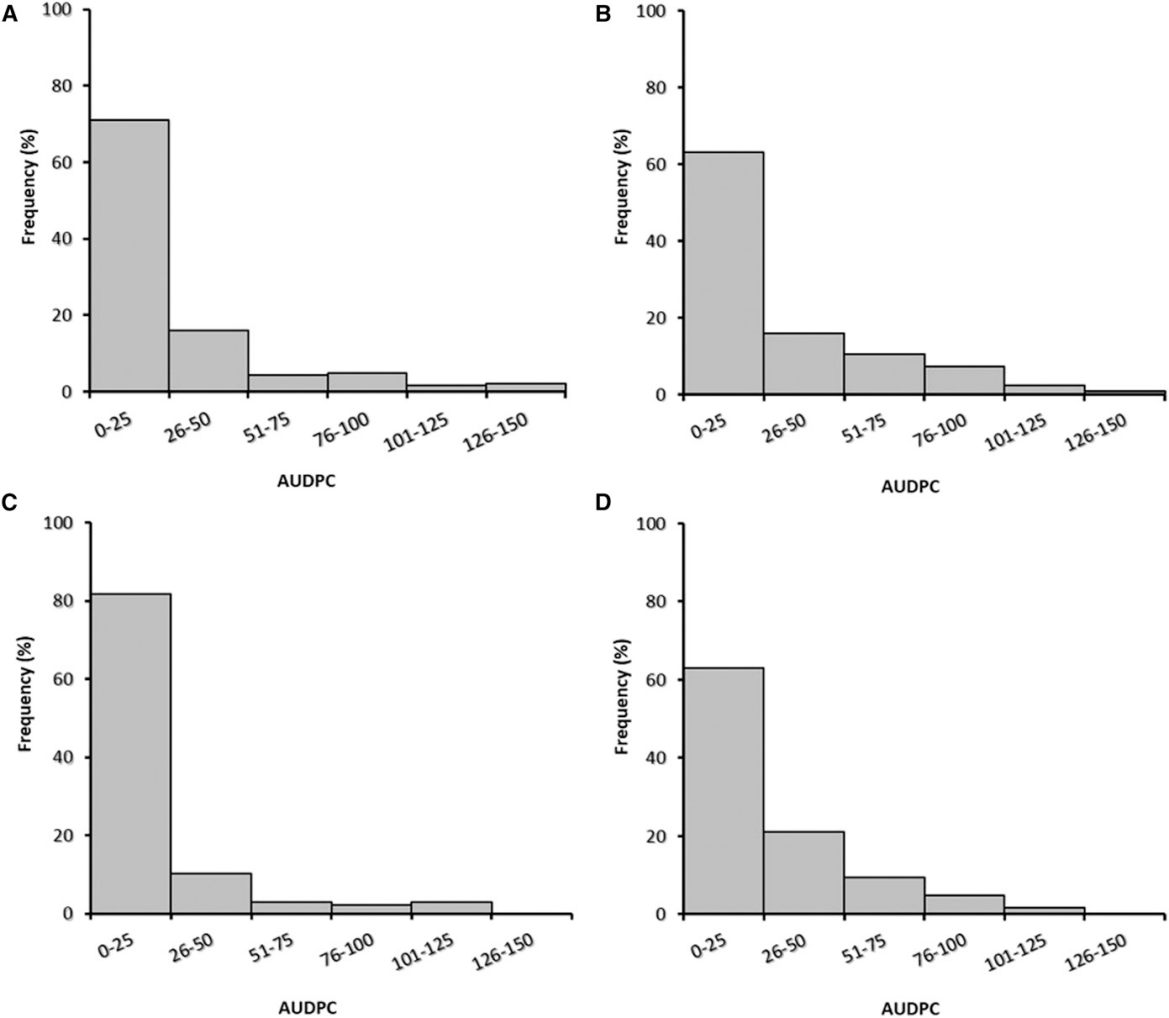
Frequency distribution of area under the disease progress curve (AUDPC) values for plant collapse caused by *Phytophthora cactorum* for quantitative trait locus discovery populations (A and B) and validation sets (C and D) evaluated during the 2013–2014 (left) and the 2014–2015 (right) seasons.

A wide range of phenotypic variability for plant collapse caused by *P. cactorum* was observed during both seasons. Plant collapse rates for the QTL discovery population sets and the validation sets ranged from 0 to 100% in both seasons. The AUDPC values for the discovery populations ranged from 0 to 130 in the 2013–2014 season and from 0 to 131 in the 2014–2015 season. For the most susceptible genotypes, plant collapse began 1–2 wk after inoculation. Of the plants that collapsed, ∼5% were sampled for isolation of pathogens, and *P. cactorum* was recovered from all. Frequency distributions were highly skewed toward low values (resistance) with similar distributions for the validation sets (Figure 2). Full-sib family means ranged from 0 to 60 in 2013–2014 and from 0.5 to 66 in 2014–2015 (Figure S5 and Figure S6).

### Genotyping and linkage mapping

In the 2013–2014 and 2014–2015 discovery population sets, 57,602 and 35,979 SNPs, respectively, were polymorphic. The mapping and curation process resulted in a final map of 3799 SNPs spanning 19.9 Morgan that maximized polymorphism in both discovery population sets. In 2013–2014 the mean MAF was 0.29, with 96% of markers having MAF between 0.1 and 0.5 (Figure S3). In 2014–2015 the mean MAF was 0.28, with 97% of markers having MAF between 0.1 and 0.5 (Figure S4). Of the 3799 SNPs, 92% were categorized as poly-high resolution, presenting all three possible SNP genotype classes (*AA*, *AB*, and *BB*), whereas the remaining 5% lacked one of the homozygous genotype classes (so called no-minor homozygote markers). Distributions of MAF across LGs in the two population sets were quite similar (Figure S3 and Figure S4), with some LGs having relatively higher MAF (2A, 5C) and some with lower MAF (3B, 4A) overall. While the average density of the map was 1.9 SNPs per cM, markers were not evenly distributed on several LGs. The six largest gaps between markers were 27.6 cM (6B), 26.5 cM (7AII), 18.2 cM (7B), 18.0 cM (6D), 15.7 cM (6A), and 15.5 cM (1B). For the remaining LG, the largest gaps ranged from 1.4 to 13.8 cM. Estimates of pairwise LD corrected for relatedness ($r_{v}^{2}$) varied among LGs, ranging from 0.0 (for one of the smallest fragments on one side of a homozygous region with few markers) to 0.17 (Gezan *et al.* 2017). Average $r_{v}^{2}$ genome-wide was 0.04 in 2013–2014 and 0.05 in 2014–2015.

### QTL detection and phenotypic effects

**Figure 3:**
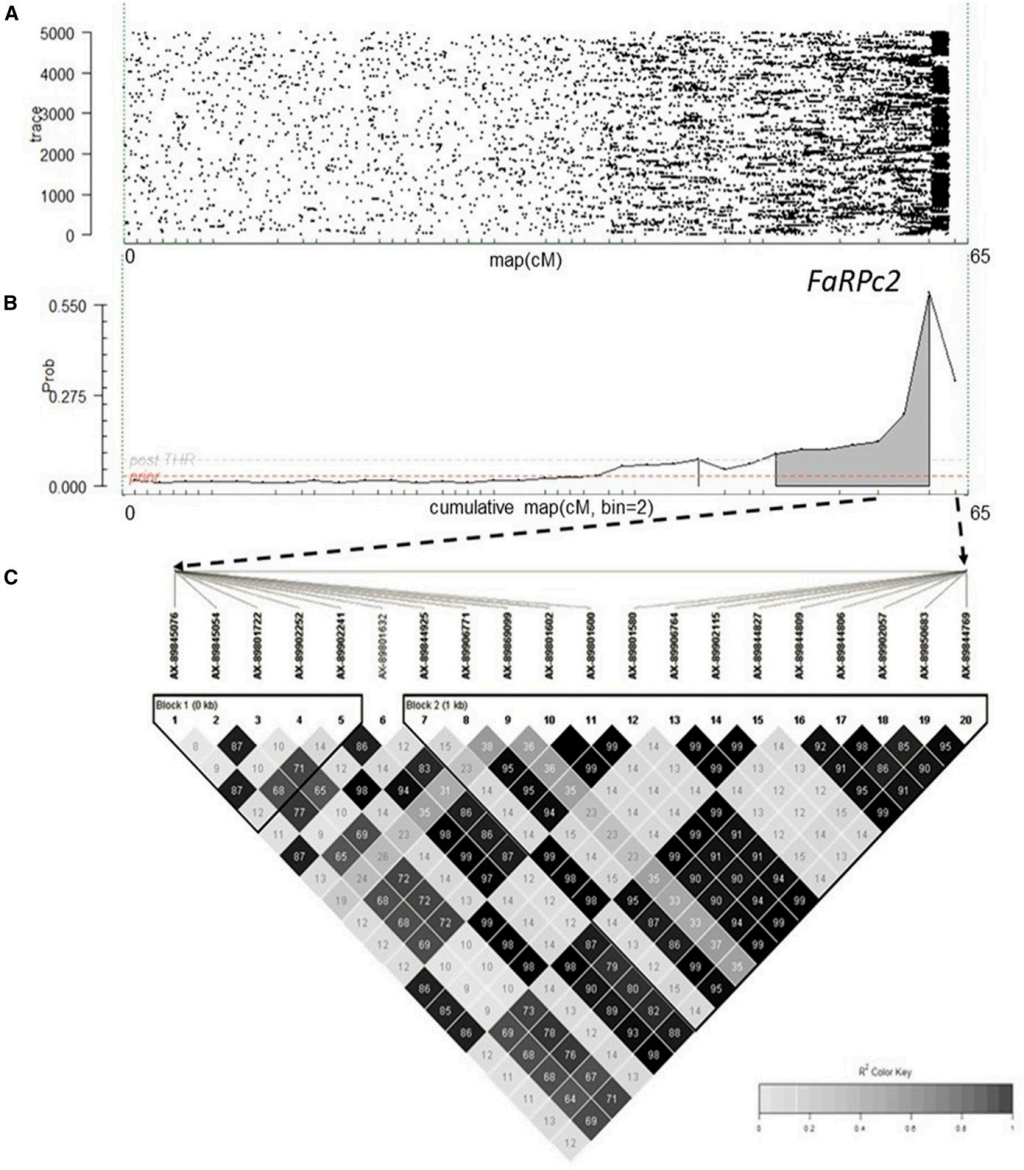
Posterior evidence of a QTL conferring resistance to crown rot caused by *Phytophthora cactorum* on LG 7D. The *x*-axis represents genetic locations in centiMorgans for LG 7D, and the *y*-axis represents (A) traces of QTL and (B) posterior intensities based on an additive genetic model executed by FlexQTL software. These figures are representative of FlexQTL outputs obtained from three independent runs. The black and white colored matrix (C) represents pair-wise linkage disequilibrium (*r*^2^) among SNP markers on LG 7D for unselected seedlings (discovery populations). Triangular boxes represent haploblocks within the *FaRPc2* region calculated using the four-gamete method. SNP probes in bold were used for haplotype construction. LG, linkage group; QTL, quantitative trait locus; SNP, single nucleotide polymorphism.

Genome-wide analysis for the 2013–2014 discovery population showed evidence for a two QTL model (2lnBF_10_ = 6.0–6.8) in independent MCMC simulations using 1 as prior number of QTL. Analyses with prior number of QTL of 3 showed positive evidence for a three QTL model (2lnBF_10_ = 2.0–2.6). However, the presence and location of only one QTL was consistent across all four replicate runs, with decisive evidence (2lnBF_10_ = 19.1–34.4) for a QTL position at the very distal part (63 cM) of LG 7D. Three replicate runs of LG-specific analysis for LG 7D showed similar positive to strong evidence (2lnBF_10_ = 5.6–6.4) for the QTL at the same location (Figure 3, Figure S7, and Figure S8). The QTL peak spanned SNP markers AX-89845076 and AX-89844769, with map distances from 63.2 to 64.6 cM. The PVE by this locus across replicate runs was between 13.7 and 20.2%. Narrow-sense heritability estimates for the genome-wide analysis ranged between 0.26 and 0.27, and for LG 7D did not vary from 0.24 across replicate runs (Table 2).

**Table 2:** Genetic parameters for plant collapse caused by *Phytophthora cactorum* and proportion of phenotypic variance explained by the *FaRPc2* locus for two QTL discovery populations evaluated during the 2013–2014 and 2014–2015 seasons, respectively

	FlexQTL*^a^*							
	Genome-wide		LG 7D		Proc GLM*^b^*		ASReml*^c^*	
Parameter	2013–2014	2014–2015	2013–2014	2014–2015	2013–2014	2014–2015	2013–2014	2014–2015
* H^2^*							0.32 (0.04)	0.38 (0.04)
* h^2^*	0.26–0.27	0.32–0.39	0.24	0.32–0.33			0.27 (0.10)	0.36 (0.12)
* d*^2^							0.00 (0.00)	0.01 (0.07)
* i*^2^							0.05 (0.07)	0.01 (0.09)
PVE (%)	14.8–20.2	20.3–24.2	13.7–16.7	17.8–24.3	25.3	23.7		

QTL, quantitative trait locus; LG, linkage group; PVE, phenotypic variation explained; AUDPC, area under the disease progress curve; SNP, single nucleotide polymorphism.

a Narrow-sense heritability (*h^2^*) and PVE (%) from FlexQTL outputs using AUDPC data for both genome-wide and LG-specific analyses represent the range of values from separate runs with different starting seeds.

b The coefficient of determination (*R*^2^) estimated in SAS was used to determine the phenotypic variance explained (%) by the allele combination at the *FaRPc2* locus. Putative alleles were assigned to SNP haplotypes based on haplotype/diplotype effects.

c ASReml outputs generated from 0.1 plant collapse data from each of three clonal replicates per individual. SEs are shown in parentheses. Parameters: broad-sense heritability (*H^2^*), narrow-sense heritability (*h^2^*), dominance variance (*d*^2^), and epistatic variance (*i*^2^).

Genome-wide QTL analysis results for the 2014–2015 populations were consistent with those for the 2013–2014 populations, with decisive evidence (2lnBF_10_ = 20.5–32.1) for one QTL on LG 7D. The same peak with mode near 63 cM was detected in all replicate runs. The mode for the QTL varied between 62 and 64 cM and always was within the interval for the QTL region of each of the individual replicate analyses (62–65 cM). The PVE for this region ranged from 20.3 to 24.2%, with a narrow-sense heritability estimate range of 0.32–0.39 for genome-wide analysis and 0.32–0.33 for LG 7D only (Table 2).

In summary, consistent evidence for a large-effect QTL for PhCR resistance near 63 cM on LG 7D was obtained in two complex, multifamily population sets, each control-inoculated in a different year. In both seasons, the 2lnBF_10_ for QTL on other LGs (1D, 3B, 5B, 6A, or 6B) were either below five or the positions were never consistent across all four replicate analyses and were not considered for further analyses. We named the QTL on LG 7D as *FaRPc2* (*Fragaria* Resistance to *P. cactorum* locus/gene 2), since the first described resistance locus in *Fragaria* was identified on LG 6 of diploid *F. vesca* (Davik *et al.* 2015).

Heritability estimates from FlexQTL outputs based on AUDPC data showed similar patterns to estimates generated using binomial data from each clonal replicate in ASReml (Table 2). Broad-sense and narrow-sense heritabilities for plant collapse caused by *P. cactorum* were both higher in the second season (*H*^2^ = 0.32 ± 0.04, *h*^2^ = 0.27 ± 0.10 in 2013–2014 and *H*^2^ = 0.38 ± 0.04, *h*^2^ = 0.36 ± 0.12 in 2014–2015). Little nonadditive variance was detected in either population/season (*d*^2^ and *i*^2^, Table 2).

### Haplotype characterization and validation of QTL effects

**Figure 4:**
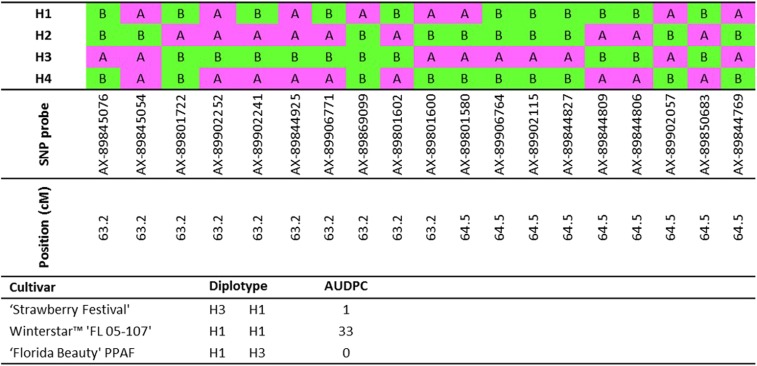
SNP calls and map positions on linkage group 7D for 19 Affymetrix Axiom IStraw90 probes comprising the four most abundant haplotypes in the *FaRPc2* QTL region. Diplotypes and AUDPC values are shown for publicly available University of Florida strawberry cultivars tested in the 2014–2015 validation set. AUDPC, area under the disease progress curve; QTL, quantitative trait locus; SNP, single nucleotide polymorphism.

Nineteen SNP probes in the *FaRPc2* region were selected to span the QTL C.I. This interval contained two haploblocks meeting at roughly the middle of the interval (Figure 3, Figure S8, and Figure S9), and care was taken to choose probes covering both blocks. These SNPs gave rise to fourteen unique haplotypes for the 2013–2014 QTL discovery population set. Four haplotypes (^1^H–H4) accounted for 97% of the haplotypes in this population (Figure 4). The remaining 10 (H5–H14) were recombinants of the four haplotypes, with no individual haplotype occurring more than once. The same four haplotypes (^1^H–H4) accounted for 93% of the total haplotypes in the 2014–2015 discovery population set. Two new haplotypes came from new lineages and accounted for 6% of the total haplotypes. For further comparisons among the two discovery population sets and validation sets, only haplotypes ^1^H–H4 were analyzed. The haplotypes of 189 and 220 individuals from the 2013–2014 to 2014–2015 validation sets, respectively, could be unambiguously assigned.

**Figure 5:**
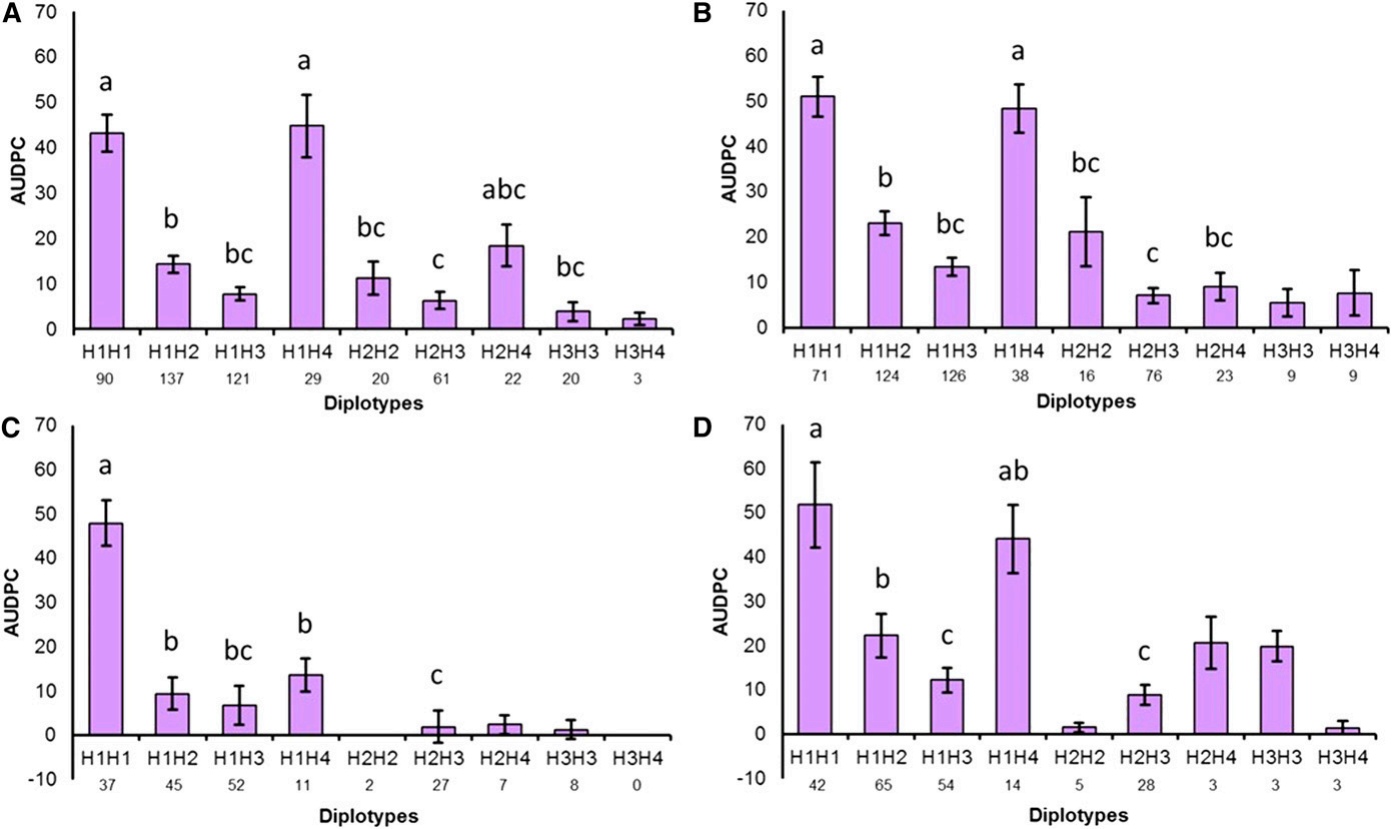
AUDPC effects of diplotypes representing the four most common SNP haplotypes at the *FaRPc2* locus for resistance to *Phytophthora cactorum* for two QTL discovery populations (A and B) and two validation sets (C and D) evaluated during the 2013–2014 (left) and 2014–2015 (right) seasons. Different letters above each bar represent statistically significant differences (*P* < 0.05), as determined by the Steele–Dwass nonparametric multiple comparison test for diplotypes having sample sizes of 10 or greater. Diplotype sample sizes are shown below the diplotype designations. SE bars were generated from a GLM analysis to illustrate variation around the mean diplotype effect. AUDPC, area under the disease progress curve; QTL, quantitative trait locus; SNP, single nucleotide polymorphism.

Estimation of diplotype effects showed that ^1^H|^1^H and ^1^H|H4 had the highest AUDPC (highest susceptibility). The H4|H4 diplotype was not represented by any individual tested. In contrast, diplotypes containing H2 and H3 had negative effects on AUDPC, suggesting that these haplotypes are associated with a resistance allele or alleles (Figure 5A). Diplotype effects in the 2014–2015 discovery population set were very similar to those observed in the previous season (Figure 5B). The H2 and H3 haplotypes both appear to be dominant to the haplotypes associated with susceptibility, since the ^1^H|H3 and H3|H3 diplotype effects were similar in magnitude, as were ^1^H|H2 and H2|H2. In addition, H3 appeared to have a stronger resistance affect than H2, since the AUDPC effect of the ^1^H|H3 diplotype tended to be lower than for the ^1^H|H2 in both seasons. Validation set diplotype effects (Figure 5, C and D) displayed similar patterns to those observed in the discovery population sets, including the stronger resistance effect of H3, though the relative diplotype effects were less consistent, which might be due to smaller sample sizes, particularly for diplotypes not containing the most abundant haplotypes. Haplotypes H2 and H3 largely appeared to arise from different pedigree lineages. For instance, almost all H2 could be traced back to two breeding selections FL 06-134 (Figure S10) and FL 07-196. The pedigrees for these two selections contained some founders that were unique compared to pedigrees containing H3. However, haplotype origins could not be traced further with confidence, since few earlier clones were still available in the germplasm collection.

To further investigate the phenotypic effects of the *FaRPc2* locus on resistance and on production traits, haplotypes of individuals in all population sets were assigned to putative QTL alleles for resistance and susceptibility under a simple biallelic model. Haplotypes H2 and H3 were assigned to the *FaRPc2* QTL allele for resistance, while ^1^H and H4 were assigned to the *farpc2* allele for susceptibility. Based on these assignments, the PVE by QTL-genotypes in the discovery populations were 25.3% in 2013–2014 and 23.7% in 2014–2015 (Figure S11 and Table 2). The PVE by QTL-genotypes in the validation sets were 37.6% in 2013–2014 and 31.5% in 2014–2015. In the 2013–2014 validation set there was a significant (*P* < 0.05) effect of *FaRPc2* QTL-genotypes on production traits SSC, TFN, and TC. However, the effect was negligible and was not consistent with the dosage of the resistant allele. The effect of QTL-genotypes was not significant for any of the production traits for the 2014–2015 validation set.

## Discussion

Representative subsets of families from the UF strawberry breeding population were utilized to identify a major QTL on LG 7D of octoploid strawberry, which we named *FaRPc2*. Pedigree-based analysis in FlexQTL consistently detected the QTL with decisive evidence across two large, genetically complex and distinct (but highly related) discovery populations, each control-inoculated and tested in a different year. Furthermore, the presence and effects of the QTL were validated in separate sets of cultivars and breeding selections via analysis of SNP diplotypes spanning the *FaRPc2* region.

### Heritability and PVE

A multifamily mapping approach combined with haplotype analysis allowed the conclusion that *FaRPc2* is a large-effect locus with dominant QTL–allele interactions and with significant effects across breeding germplasm. Estimates of PVE by *FaRPc2* from FlexQTL outputs were close in magnitude to narrow-sense heritability estimates from FlexQTL outputs (0.24–0.27 in 2013–2014 and 0.32–0.39 in 2014–2015) (Table 2). The similarity in magnitude of the PVE and heritability estimates suggest that the *FaRPc2* locus accounts for most of the genetic variation that exists in the breeding population for resistance to PhCR. This conclusion is strengthened by the similarity of FlexQTL heritability estimates based on AUDPC data with ASReml estimates from a model using plant collapse data from individual clonal replicates (0/1 scoring). Furthermore, the PVE from GLM analyses using haplotype-derived *FaRPc2* genotypes were 25.3% in 2013–2014 and 23.7% in 2014–2015, again quite similar to estimates using other methods (Table 2). Finally, the similarity between genome-wide and LG 7D-specific heritabilities from FlexQTL also rules out the presence of an additional major QTL in genomic regions that were not represented by SNP markers (see *Materials and Methods*).

The proportions of PVE by *FaRPc2* were not obtained for the validation sets via FlexQTL; however, those estimated by GLM from the *FaRPc2* QTL-genotype analysis were higher (31.5–37.6%) than those obtained for the discovery populations using either approach (Figure S11 and Table 2). The QTL discovery populations included only three clonal replicates per seedling individual, maximizing the number of seedlings tested to better represent the breadth of diversity in the breeding program for QTL detection. Conversely, the QTL validation experimental design allowed up to 20 runner plants to be tested per individual to more accurately ascertain the magnitude of genetic effects at the *FaRPc2* locus. Thus, the true effect of the *FaRPc2* locus on plant collapse caused by *P. cactorum* is probably closer to 35% of the phenotypic variation.

Since resistance could be genetically negatively correlated with other traits of horticultural interest, six production traits were measured in adjacent, noninoculated trials of the same validation sets. The effects of *FaRPc2* QTL-genotypes in the validation sets on the six production traits were either small and inconsistent with QTL allele dosage, or nonsignificant. These results are consistent with the conclusion that breeding for resistance using *FaRPc2* should not negatively influence these other important traits.

### P. cactorum resistance loci in other studies and the naming of FaRPc2

The *Rpc-1* locus, a major locus for resistance to *P. cactorum*, was recently described in diploid *F. vesca* (Davik *et al.* 2015). Evaluation of an F2 population obtained by selfing seedlings from a cross between resistant “Bukammen” × susceptible “Haugastøl 3” revealed a QTL located at the proximal end of LG 6, having a PVE of 72.4%. Further evaluation suggested that resistance at this locus is controlled by a single dominant gene (Davik *et al.* 2015). The mapping approaches of Davik *et al.* (2015) and our study locate markers to the physical map of the *F. vesca* pseudochromosome assembly v2.0 (Bassil *et al.* 2015, Davik *et al.* 2015), suggesting that LG 6 as described in Davik *et al.* (2015) corresponds to LG 6A in our map. However, the QTL described in our study was not only located on a different LG (7D), but also on a subgenome having the most sequence divergence from the *F. vesca* subgenome (Van Dijk *et al.* 2014). Hence, it appears that multiple unique loci conferring resistance to *P. cactorum* may exist within *Fragaria*. As this locus appears to be distinct from *Rpc-1*, and as it was the second locus found, we named it *FaRPc2* according to the naming convention used for the *FaFAD1* (Chambers *et al.* 2014) and *FaRXf1* (Roach *et al.* 2016) loci.

### QTL genotyping and experimental variation

Each of the various diplotypes containing haplotypes ^1^H–H4 showed experimental variation in AUDPC values (Figure 5). Of the ^1^H|^1^H and ^1^H|H4 individuals that are expected to be susceptible, 18.6 and 14.6%, respectively, did not show any plant collapse (AUDPC = 0). This reflects the high experimental variation typically encountered when phenotyping traits via plant collapse ratings, which is consistent with the observed low to moderate heritability estimates (0.24–0.36) in this study (Table 2). Indeed, we have observed that some highly susceptible individuals escape infection in some trials and years. Therefore, QTL genotyping of single individuals based on phenotypic (AUDPC) values will not be very robust compared to QTL genotyping based on marker haplotypes and their germplasm-wide assessed/deduced correlations with QTL effects. This is especially true because of the few number of prevalent haplotypes (4) and the considerable number of polymorphic SNP markers (19) on which they are based. This combination makes it highly probable that an identity-by-state (IBS) QTL genotyping approach could give meaningful results when compared to a more complex identity-by-descent (IBD) approach. Indeed, a comparison of FlexQTL-generated genotype probabilities (based on IBD) with SNP diplotypes (IBS) showed that the diplotypes had the best concordance with the phenotypic data. For about one quarter of the genotypes, FlexQTL-generated genotype predictions did not match the diplotypes. It is likely that the brief ancestral pedigree (only two generations previous to the discovery population sets) and the experimental variability discussed above limited the ability of FlexQTL to accurately phase and predict QTL alleles for certain individuals.

### Haplotype effects and QTL alleles

Haplotype analyses revealed a high prevalence of four *FaRPc2* SNP haplotypes (^1^H-H4) across both discovery population sets and both validation sets. The lower AUDPC mean values of individuals with diplotypes containing H2 and/or H3 suggested that these haplotypes may be associated with a functional resistance allele or alleles. In contrast, haplotypes ^1^H and H4 were associated with high AUDPC values, suggesting that they are associated with susceptible alleles. Further analysis of diplotypes showed that H2 and H3 were dominant over ^1^H and H4.

The high prevalence of a few haplotypes underlines the narrow genetic base of the investigated germplasm and may also ease the development of high-throughput diagnostic marker assays. The H2 and H3 haplotypes each have a haplotype-specific SNP allele for at least one SNP probe. For H2, this is allele *A* of AX-89801722 and for H3 this is allele *A* of the tightly linked probes AX-89906764, AX-89902115, and AX-89844827. Each of these four markers segregated in “Holiday” × “Korona,” H2 in “Korona” and H3 in “Holiday.” The favorable SNP allele for H2 must have been inherited from “Senga Sengana” through “Induka,” and the favorable SNP allele for H3 might have derived from “Fairfax,” a great-great grand-parent of “Holiday” that occurs in the parentage of many USA-bred cultivars. It is important to note that H3 showed a stronger resistance effect than H2 (Figure 5). These two haplotypes are almost always inherited through separate lineages in the discovery populations, though their origins could not be traced back to early founders (Figure S10). Thus, differences in effects and lineage provide evidence for the possibility of two distinct functional resistance alleles that may be associated with H2 and H3, though their origins are currently unknown.

The high degree of relatedness in the population and the low number of resistance-associated haplotypes is an advantage for assessing the magnitude and variability of the effects of *FaRPc2* across elite breeding germplasm. On the other hand, a major limitation of the population is that it may represent as few as two sources of resistance, which cannot be traced very well beyond the population. Because maintenance of strawberry clones is very expensive, strawberry breeders typically maintain only a fraction of ancestral genotypes. Yet retaining at least the majority of the important ancestral clones for 7 to 10 previous generations could have significantly improved the utility of the multiparental population sets in terms of tracing ancestral sources as well as improved phasing of alleles in FlexQTL. However, this would not have solved the limitation of the reduced diversity of this elite germplasm when compared to the broader diversity present in breeding programs and gene banks worldwide. It is quite likely that additional loci and/or additional alleles at *FaRPc2* contribute to resistance to *P. cactorum* in other breeding programs and in wild germplasm collections. Alternative sources of resistance could become quite important if *FaRPc2* proves not to be durable in the future.

### Potential resistance mechanisms

Infection mechanisms of pathogens in the genus *Phytophthora* involve a series of processes starting from recognition of the potential host to successful colonization and multiplication inside the host (Hardham 2001). Thus, it is possible that individuals carrying only susceptible alleles at the *FaRPc2* locus lack a protein that allows early recognition of the pathogen or that helps in defense during early colonization. For instance, the *RPc-1* locus for resistance to *P. cactorum* in *F. vesca* colocalizes with at least 69 genes putatively involved in disease resistance (Davik *et al.* 2015). Some of these genes have putative pathogen recognition functions, affect plant immunity, or regulate signaling pathways during pathogen infection. Furthermore, *Phytophthora* spp. produce phytotoxins that are highly conserved within the genus (Pernollet *et al.* 1993), and some of these toxins have been associated with disease development in tomato and apple. In the *P. cactorum*–strawberry interaction, at least one phytotoxin has been described. The PcF protein from *P. cactorum*, an elicitor factor, triggers a hypersensitivity response in strawberry leaves (Orsomando *et al.* 2001). However, the chemical compounds and other responses involved in crown and root infection are unknown. Likewise, it is unknown which functional gene or genes reside at the *FaRPc2* locus and the effect of these on disease resistance. Since even some individuals carrying resistant alleles showed plant collapse at some point during the season in our study as well as in other studies (Rijbroek *et al.* 1997), it is likely that genes for resistance in this region act to slow pathogen infection and colonization processes, allowing plants to thrive longer during the production season, or reduce the probability for becoming infected rather than preventing infection or plant collapse completely, similar to the strawberry *Rpf1* locus for resistance to the related strawberry pathogen *P. fragariae* (Van de Weg 1997c, Van de Weg *et al.* 1997).

### Conclusions

A pedigree-based QTL detection approach in complex, multiparental population sets allowed the detection of a large-effect QTL for resistance to crown rot caused by *P. cactorum*, which we name *FaRPc2*. These population sets also allowed the estimation and validation of *FaRPc2* resistance allele effects across many genetic backgrounds. Four distinct SNP haplotypes at this locus were predominant, two of which were associated with decreased mortality (H2 and H3). Haplotype effects were quite similar across populations and years, providing reliable evidence for QTL presence and effects. Differences in magnitude of resistant effects for H2 and H3 raise the possibility of multiple functional resistance alleles in the breeding population. The information generated will facilitate breeding strategies to increase the frequency of resistant QTL-alleles in strawberry breeding germplasm and the release of *P. cactorum*-resistant cultivars. Fine-mapping of the *FaRPc2* locus is underway toward the development of closely-linked, high-throughput markers for marker-assisted selection and toward functional characterization of the locus.

## Supplementary Material

Supplemental material is available online at http://www.g3journal.org/lookup/suppl/doi:10.1534/g3.117.042119/-/DC1.
