# Supplementary material for: Pedigree-Based Analysis in a Multiparental Population of Octoploid Strawberry Reveals QTL Alleles Conferring Resistance to Phytophthora cactorum
Source: G3 (Bethesda). 2017 Jun 5;7(6):1707–19. doi: 10.1534/g3.117.042119 (PMC5473751; doi:10.1534/g3.117.042119)
Supplement: Supplementary file 1 [file 1707FigureS1.pdf]

2013-14 discovery population

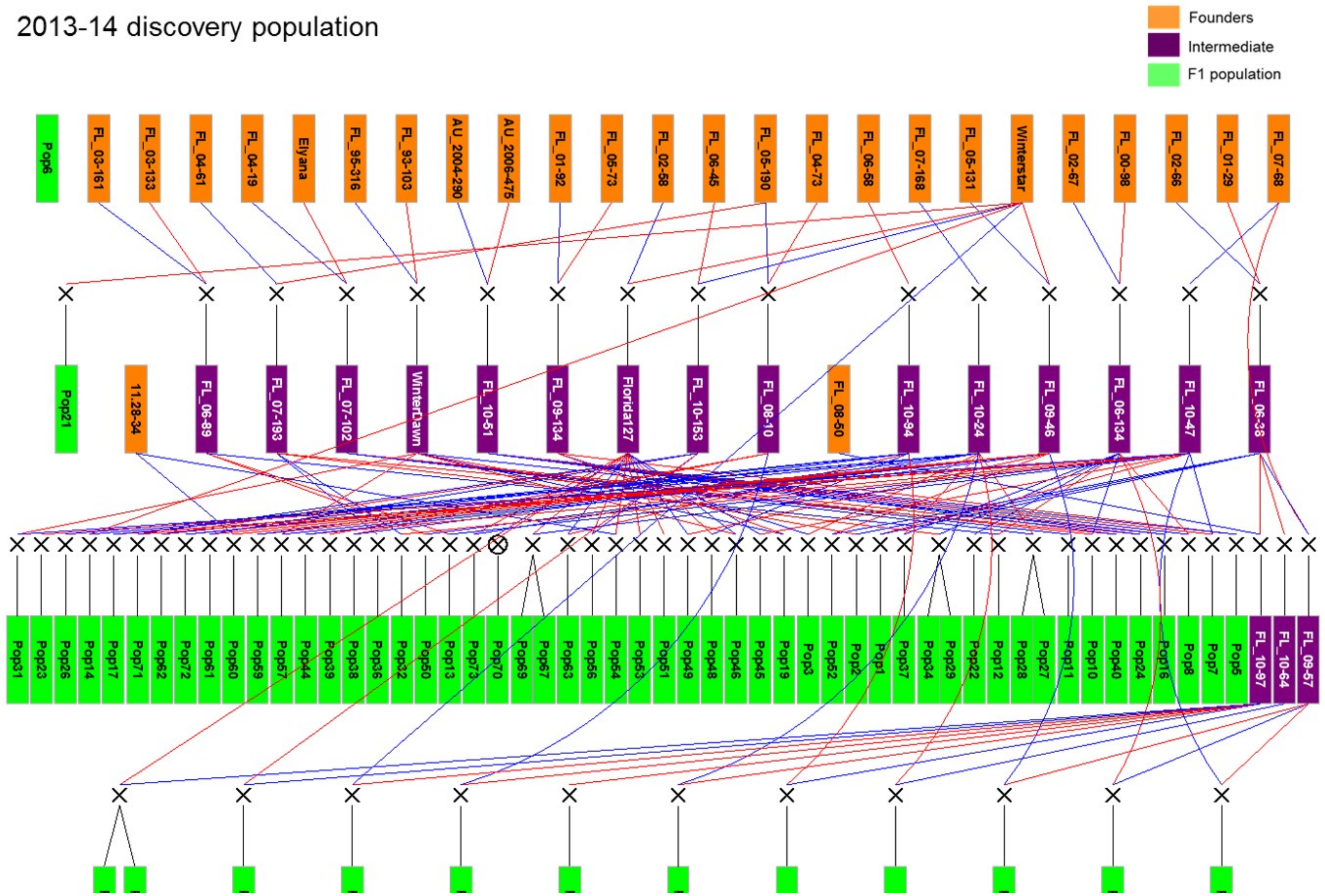

Supplementary Figure S1 Overview of pedigree connectivity of a multiparental QTL discovery population set in 2013-14. Orange = founders with unknown parentage; purple = intermediate parents and selections; and green = seedling populations phenotyped for resistance to Phytophthora crown rot
