# Supplementary material for: Pedigree-Based Analysis in a Multiparental Population of Octoploid Strawberry Reveals QTL Alleles Conferring Resistance to Phytophthora cactorum
Source: G3 (Bethesda). 2017 Jun 5;7(6):1707–19. doi: 10.1534/g3.117.042119 (PMC5473751; doi:10.1534/g3.117.042119)
Supplement: Supplementary file 2 [file 1707FigureS2.pdf]

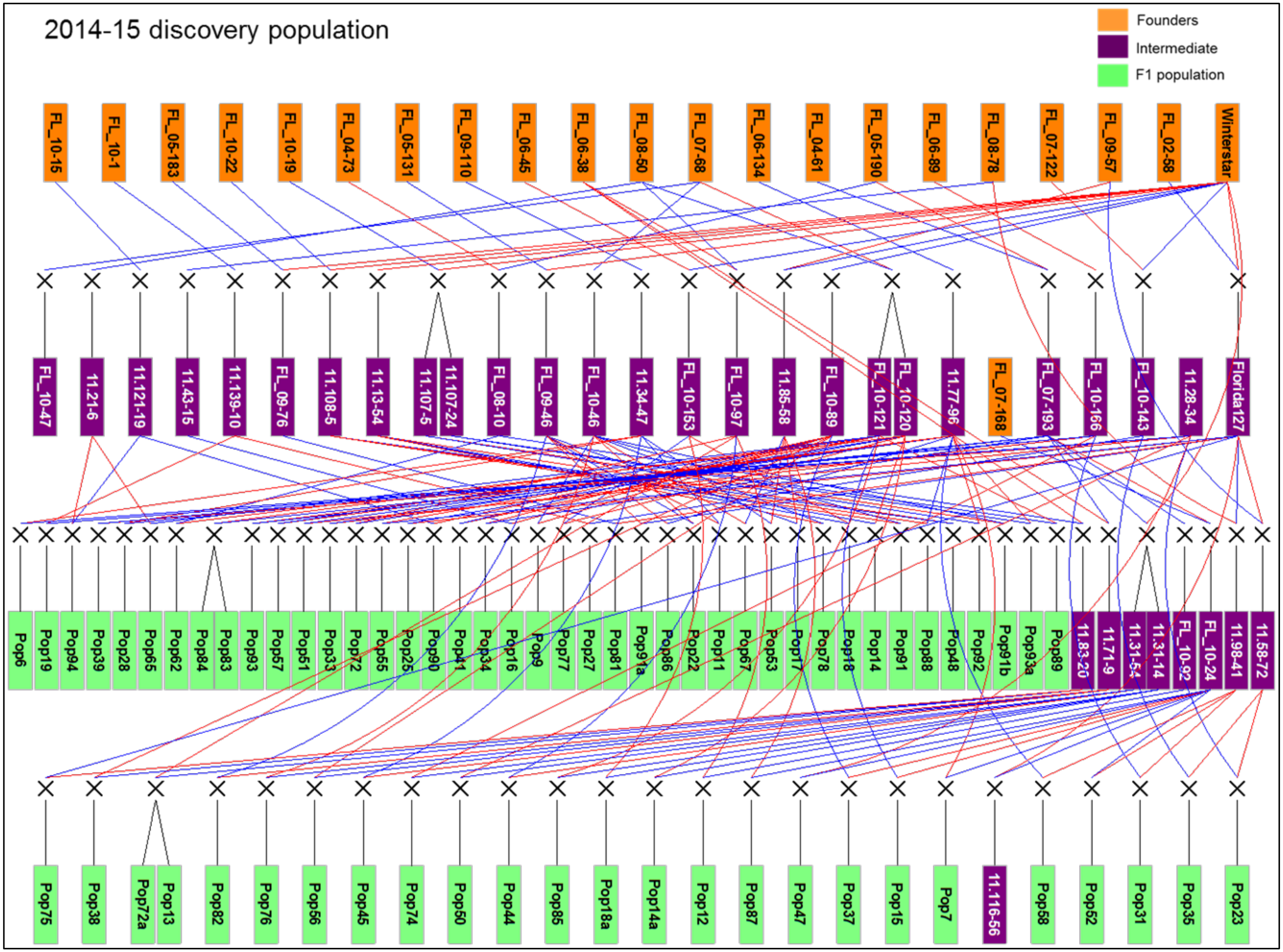
2014-15 discovery population
Founders
Intermediate
F1 population
FL_10-15
FL_10-1
FL_05-183
FL_10-22
FL_10-19
FL_04-73
FL_05-131
FL_09-110
FL_06-45
FL_06-38
FL_08-50
FL_07-68
FL_06-134
FL_04-61
FL_05-190
FL_06-89
FL_08-78
FL_07-122
FL_09-57
FL_02-58
Winterstar
FL_10-47
11.21-6
11.121-19
11.43-15
11.139-10
FL_09-76
11.108-5
11.13-54
11.107-5
11.107-24
FL_08-10
FL_09-46
FL_10-46
11.34-47
FL_10-153
FL_10-97
11.85-58
FL_10-89
FL_10-121
FL_10-120
11.77-96
FL_07-168
FL_07-193
FL_10-166
FL_10-143
11.28-34
Florida127
Pop6
Pop19
Pop94
Pop39
Pop28
Pop65
Pop62
Pop84
Pop83
Pop93
Pop57
Pop51
Pop33
Pop72
Pop55
Pop25
Pop90
Pop41
Pop34
Pop16
Pop9
Pop77
Pop27
Pop81
Pop91a
Pop86
Pop22
Pop11
Pop67
Pop53
Pop17
Pop78
Pop18
Pop14
Pop91
Pop88
Pop48
Pop92
Pop91b
Pop93a
Pop89
11.83-20
11.71-9
11.31-54
11.31-14
FL_10-92
FL_10-24
11.98-41
11.58-72
Pop75
Pop38
Pop72a
Pop13
Pop82
Pop76
Pop56
Pop45
Pop74
Pop50
Pop44
Pop85
Pop18a
Pop14a
Pop12
Pop87
Pop47
Pop37
Pop15
Pop7
11.116-56
Pop58
Pop52
Pop31
Pop35
Pop23

Supplementary Figure S2 Overview of pedigree connectivity of a multiparental QTL discovery population set in 2014-15. Orange = founders with unknown parentage; purple = intermediate parents and selections; and green = seedling populations phenotyped for resistance to Phytophthora crown rot
