# Supplementary material for: Pedigree-Based Analysis in a Multiparental Population of Octoploid Strawberry Reveals QTL Alleles Conferring Resistance to Phytophthora cactorum
Source: G3 (Bethesda). 2017 Jun 5;7(6):1707–19. doi: 10.1534/g3.117.042119 (PMC5473751; doi:10.1534/g3.117.042119)
Supplement: Supplementary file 4 [file 1707FigureS4.pdf]

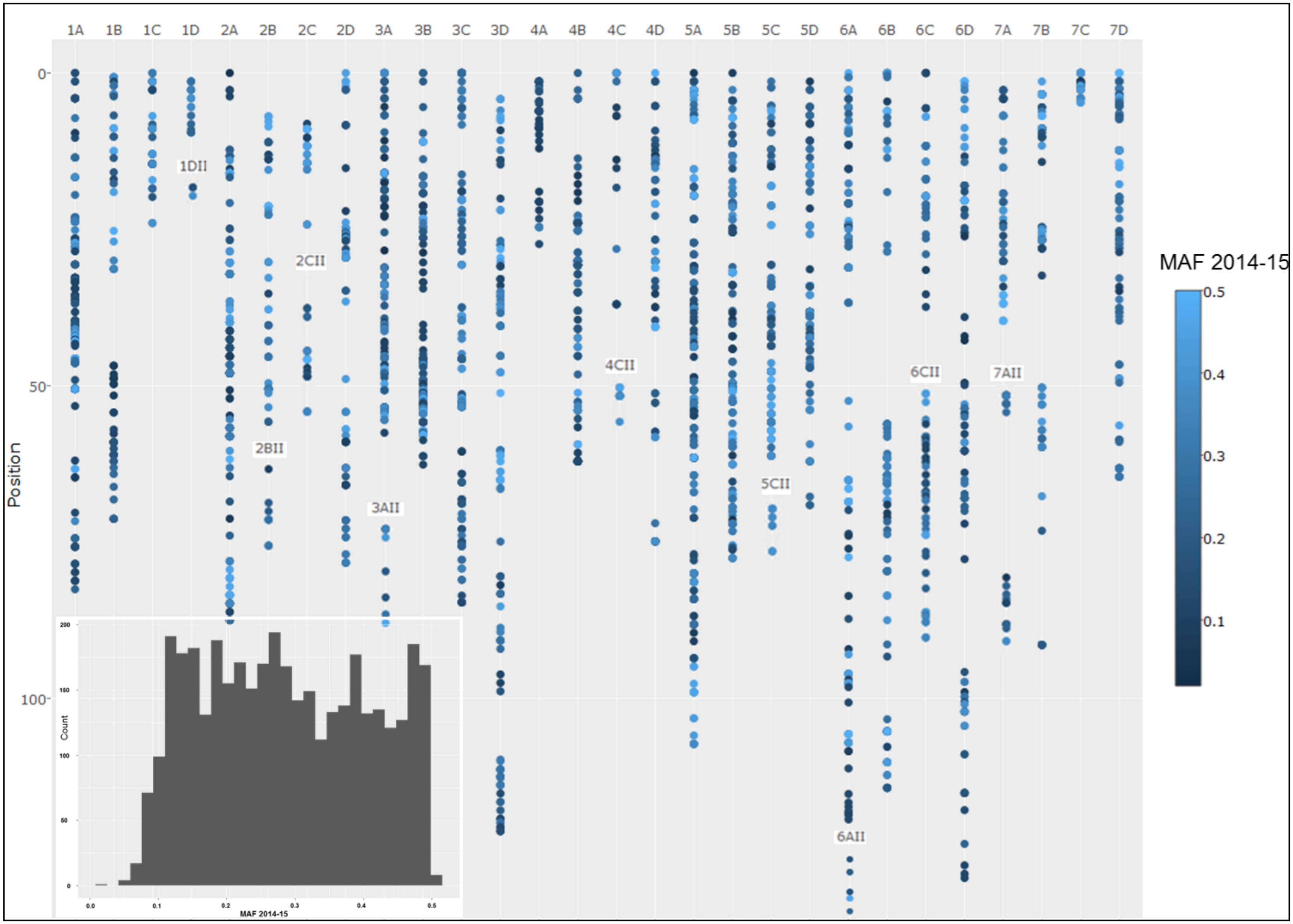

1A 1B 1C 1D 2A 2B 2C 2D 3A 3B 3C 3D 4A 4B 4C 4D 5A 5B 5C 5D 6A 6B 6C 6D 7A 7B 7C 7D
0
50
100
Position
1DII
2CII
2BII
3AII
4CII
5CII
6AII
6CII
7AII
MAF 2014-15
0.5
0.4
0.3
0.2
0.1
Count
MAF 2014-15

Supplementary Figure S4 Distribution of markers and their minor allele frequencies for the linkage map and multiparental population set used for QTL discovery in 2014-15
