# Supplementary material for: Pedigree-Based Analysis in a Multiparental Population of Octoploid Strawberry Reveals QTL Alleles Conferring Resistance to Phytophthora cactorum
Source: G3 (Bethesda). 2017 Jun 5;7(6):1707–19. doi: 10.1534/g3.117.042119 (PMC5473751; doi:10.1534/g3.117.042119)
Supplement: Supplementary file 5 [file 1707FigureS5.pdf]

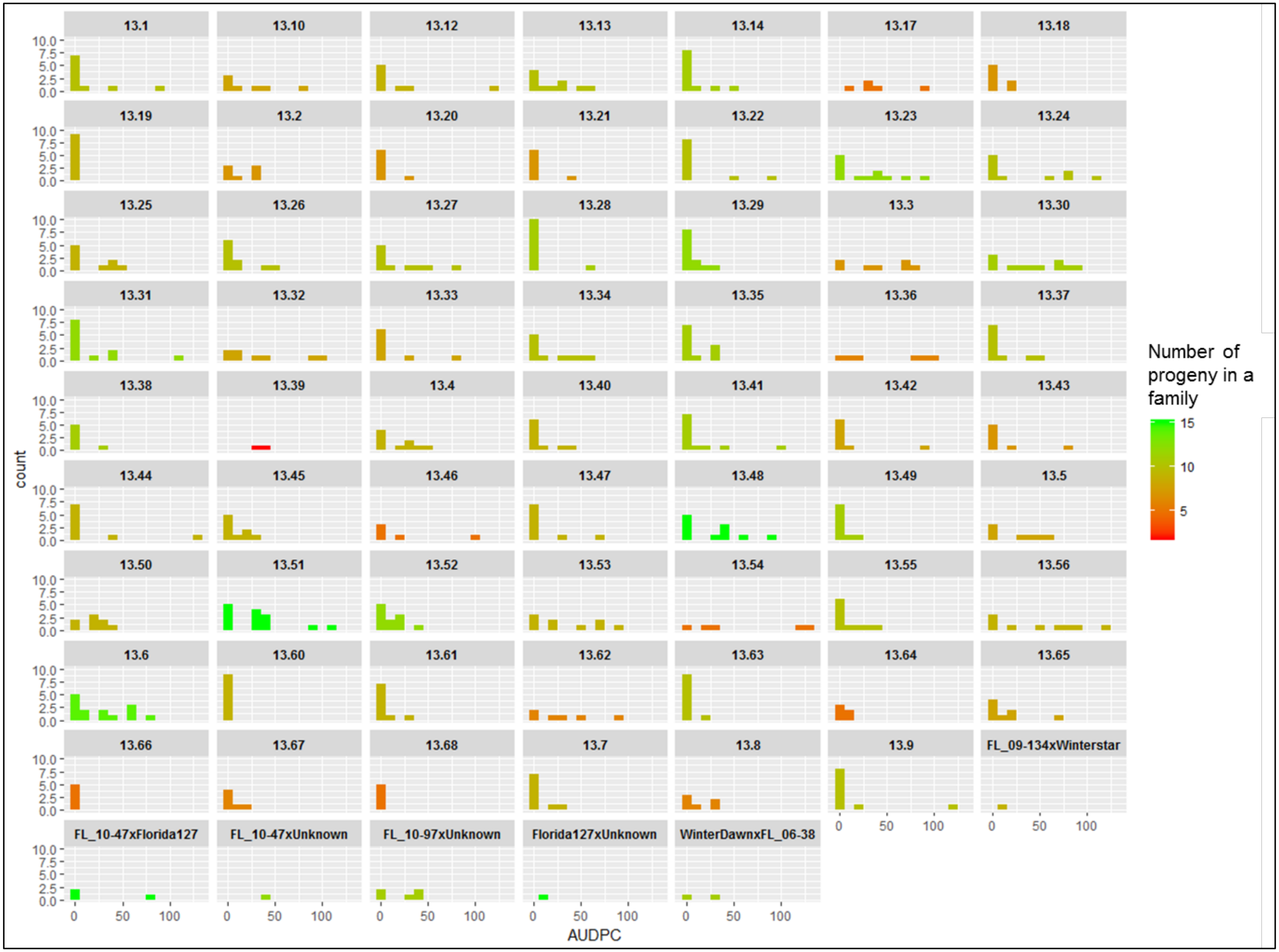
13.1
13.10
13.12
13.13
13.14
13.17
13.18
13.19
13.2
13.20
13.21
13.22
13.23
13.24
13.25
13.26
13.27
13.28
13.29
13.3
13.30
13.31
13.32
13.33
13.34
13.35
13.36
13.37
13.38
13.39
13.4
13.40
13.41
13.42
13.43
13.44
13.45
13.46
13.47
13.48
13.49
13.5
13.50
13.51
13.52
13.53
13.54
13.55
13.56
13.6
13.60
13.61
13.62
13.63
13.64
13.65
13.66
13.67
13.68
13.7
13.8
13.9
FL_09-134xWinterstar
FL_10-47xFlorida127
FL_10-47xUnknown
FL_10-97xUnknown
Florida127xUnknown
WinterDawnxFL_06-38
count
AUDPC
Number of progeny in a family
15
10
5

Supplementary Figure S5 AUDPC distributions in response to inoculation with *Phytophthora cactorum* for full-sib families in the 2013-14 QTL discovery population set. Families without preassigned family numbers were labeled with the female and male parents
