# Supplementary material for: Pedigree-Based Analysis in a Multiparental Population of Octoploid Strawberry Reveals QTL Alleles Conferring Resistance to Phytophthora cactorum
Source: G3 (Bethesda). 2017 Jun 5;7(6):1707–19. doi: 10.1534/g3.117.042119 (PMC5473751; doi:10.1534/g3.117.042119)
Supplement: Supplementary file 6 [file 1707FigureS6.pdf]

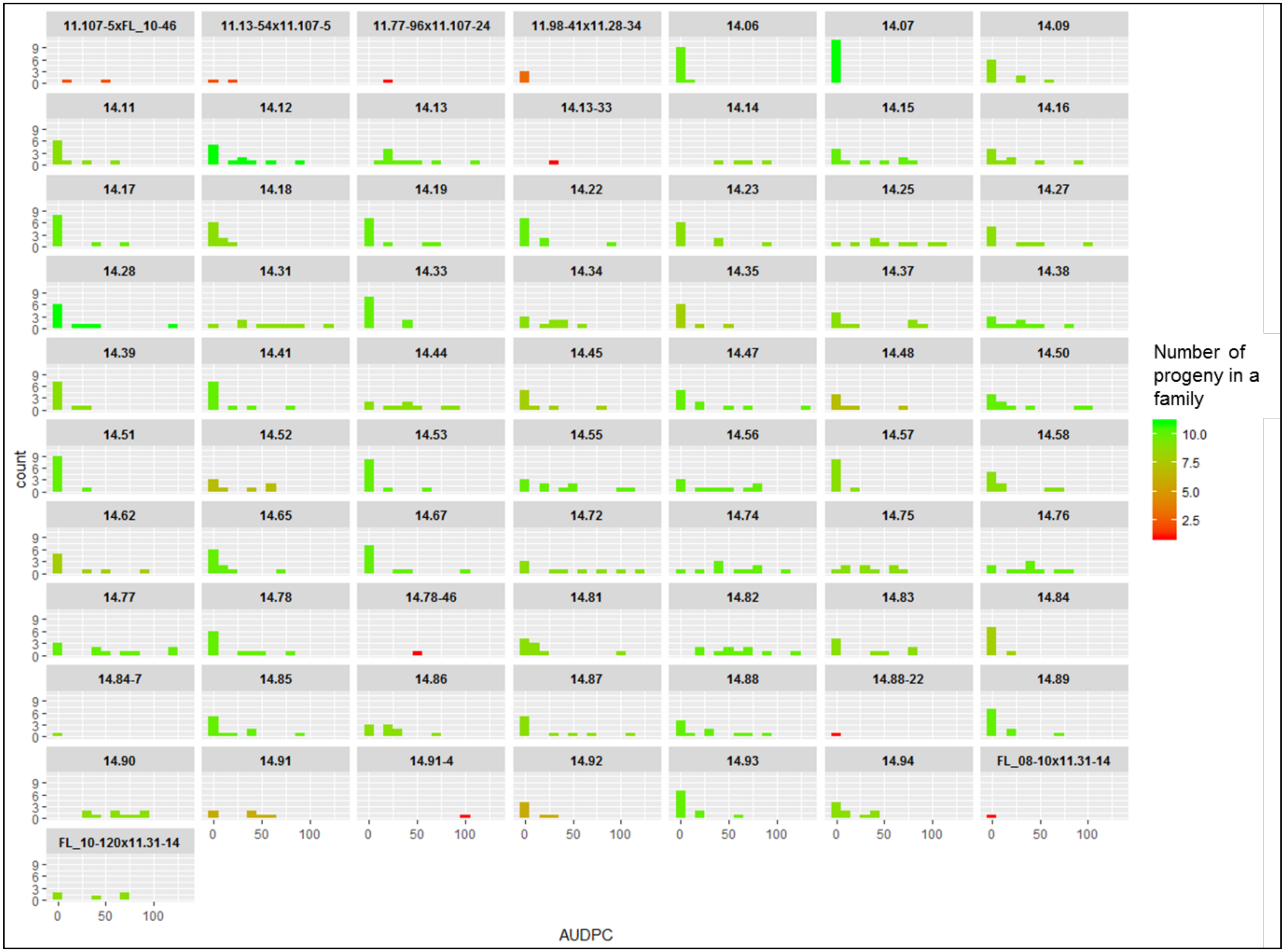
11.107-5xFL_10-46
11.13-54x11.107-5
11.77-96x11.107-24
11.98-41x11.28-34
14.06
14.07
14.09
14.11
14.12
14.13
14.13-33
14.14
14.15
14.16
14.17
14.18
14.19
14.22
14.23
14.25
14.27
14.28
14.31
14.33
14.34
14.35
14.37
14.38
14.39
14.41
14.44
14.45
14.47
14.48
14.50
14.51
14.52
14.53
14.55
14.56
14.57
14.58
14.62
14.65
14.67
14.72
14.74
14.75
14.76
14.77
14.78
14.78-46
14.81
14.82
14.83
14.84
14.84-7
14.85
14.86
14.87
14.88
14.88-22
14.89
14.90
14.91
14.91-4
14.92
14.93
14.94
FL_08-10x11.31-14
FL_10-120x11.31-14
count
AUDPC
Number of progeny in a family
10.0
7.5
5.0
2.5

Supplementary Figure S6 AUDPC distributions in response to inoculation with *Phytophthora cactorum* for full-sib families in the 2014-15 QTL discovery population set. Families without preassigned family numbers were labeled with female and male parents
