# Supplementary material for: Pedigree-Based Analysis in a Multiparental Population of Octoploid Strawberry Reveals QTL Alleles Conferring Resistance to Phytophthora cactorum
Source: G3 (Bethesda). 2017 Jun 5;7(6):1707–19. doi: 10.1534/g3.117.042119 (PMC5473751; doi:10.1534/g3.117.042119)
Supplement: Supplementary file 7 [file 1707FigureS7.pdf]

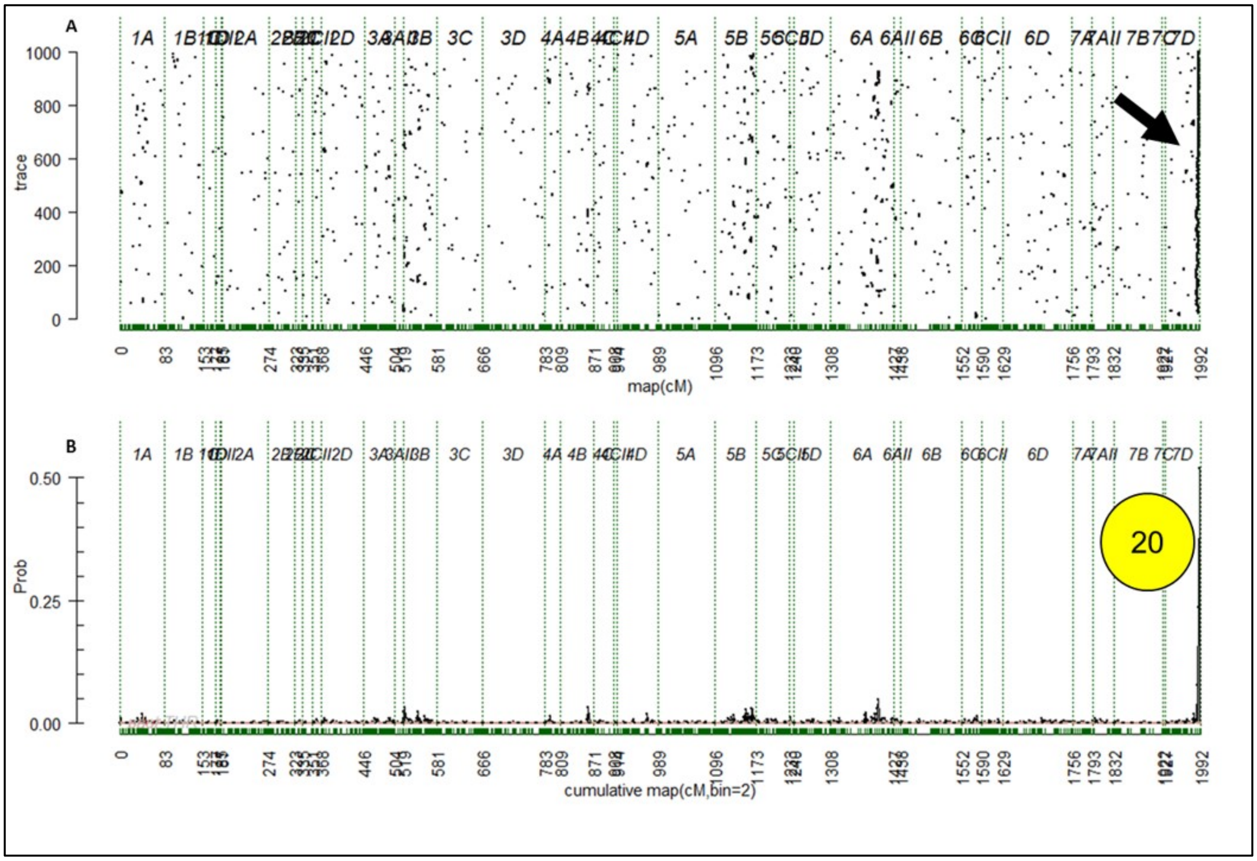

Supplementary Figure S7 Representative genome-wide QTL evidence output of FlexQTL^{TM} for resistance to *Phytophthora cactorum*. The X-axis represents cumulative genetic distance, and the Y-axis represents traces of QTL models (A) and posterior intensity (B). The number in the yellow is the Bayes Factor, a measure of decisive evidence (BF >10), for the QTL for a single run. These figures are representative of FlexQTL™ outputs obtained from four independent runs
