# Supplementary material for: Pedigree-Based Analysis in a Multiparental Population of Octoploid Strawberry Reveals QTL Alleles Conferring Resistance to Phytophthora cactorum
Source: G3 (Bethesda). 2017 Jun 5;7(6):1707–19. doi: 10.1534/g3.117.042119 (PMC5473751; doi:10.1534/g3.117.042119)
Supplement: Supplementary file 8 [file 1707FigureS8.pdf]

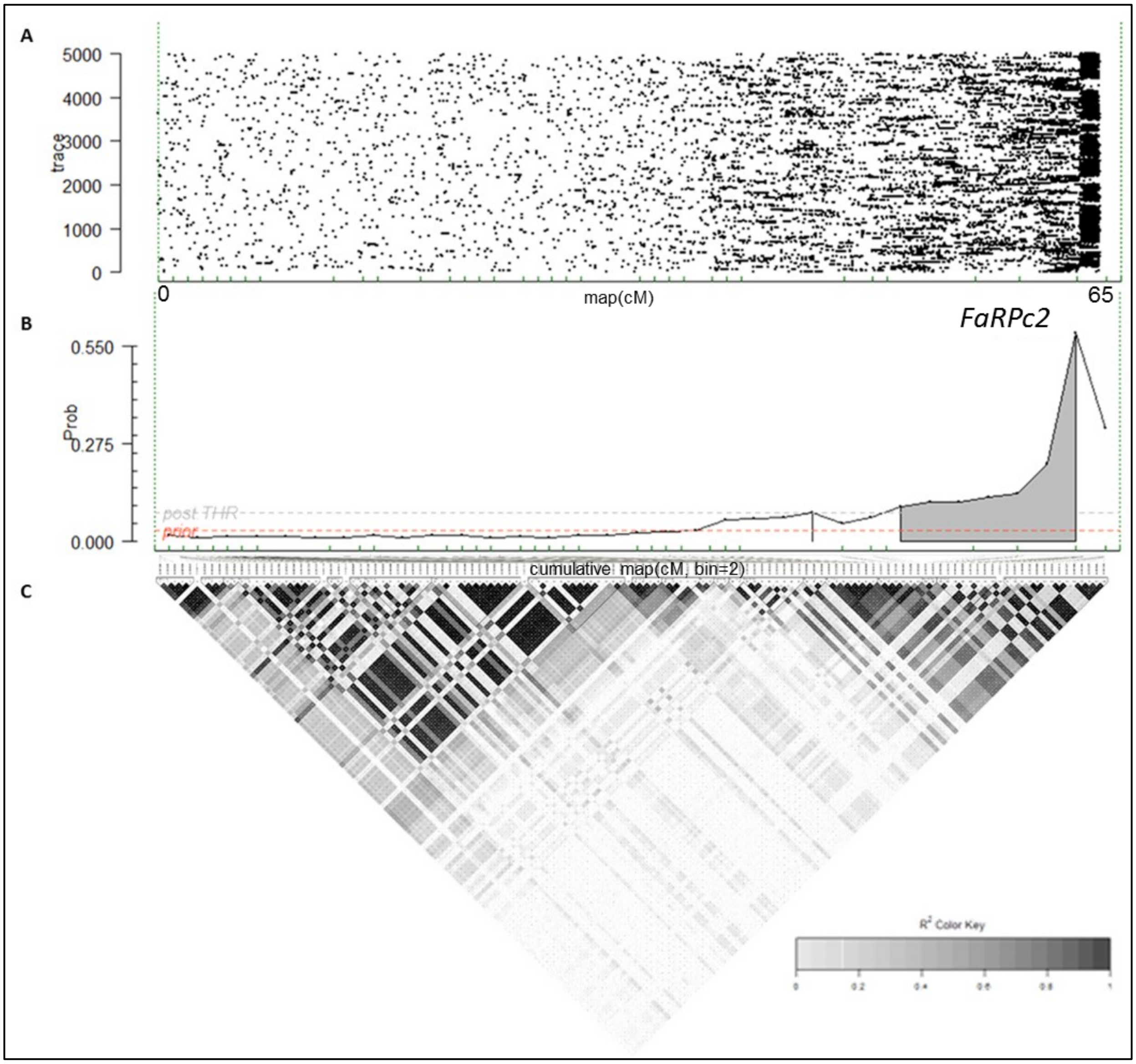

Supplementary Figure S8 Posterior evidence of a QTL conferring resistance to crown rot caused by *Phytophthora cactorum* on LG 7D. The X-axis represents genetic locations in centiMorgans, and the Y-axis represents (A) traces of QTLs and (B) posterior intensities based on an additive genetic model executed by FlexQTL™ software. These figures are representative of FlexQTL™ outputs obtained from three independent runs. The black and white colored matrix (C) represents pair-wise linkage disequilibrium (r^2) among SNP markers on LG 7D for unselected seedlings (discovery populations). The r^2 values range from 0 (white) to 1 (black). The triangular boxes represent haploblocks along LG 7D calculated using the four-gamete method in Haploview software.
