# Supplementary material for: Pedigree-Based Analysis in a Multiparental Population of Octoploid Strawberry Reveals QTL Alleles Conferring Resistance to Phytophthora cactorum
Source: G3 (Bethesda). 2017 Jun 5;7(6):1707–19. doi: 10.1534/g3.117.042119 (PMC5473751; doi:10.1534/g3.117.042119)
Supplement: Supplementary file 9 [file 1707FigureS9.pdf]

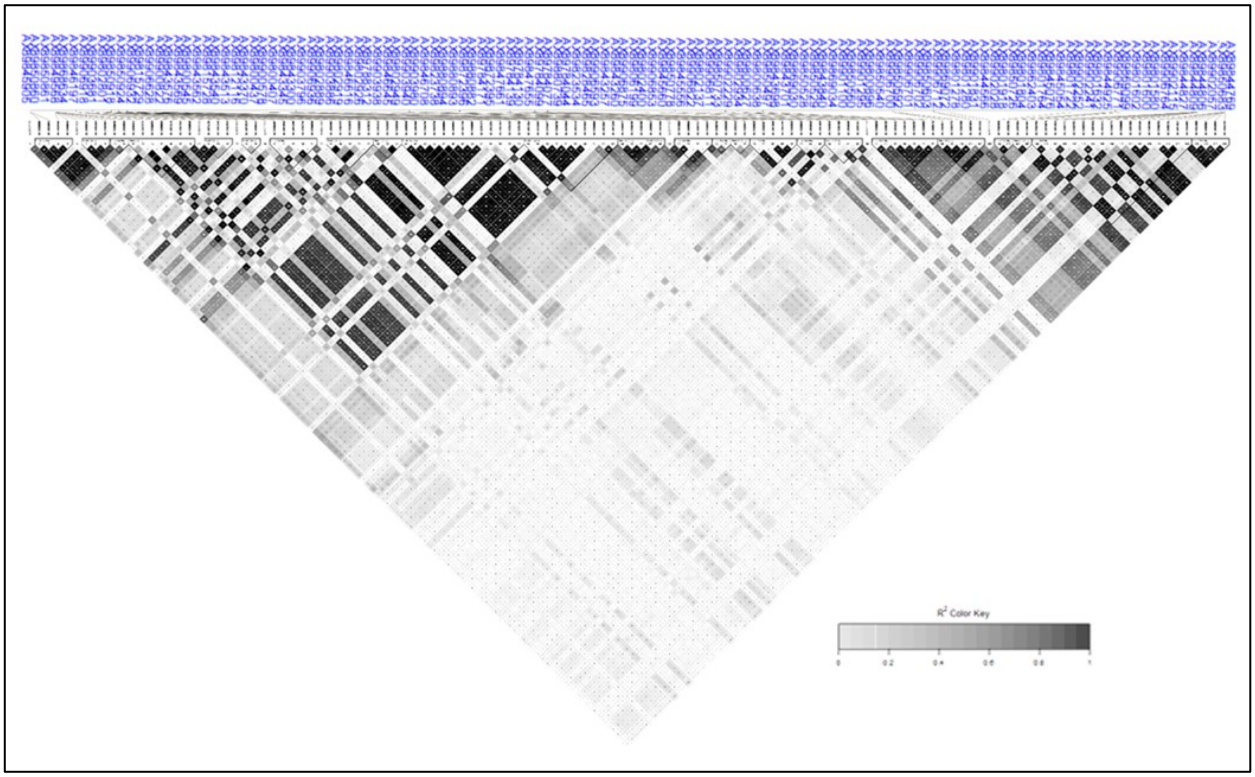

Supplementary Figure S9 Pair-wise linkage disequilibrium (r^2) among SNP markers on LG 7D using selected individuals (elite genotypes) from the validation sets. The r^2 values range from 0 (white) to 1 (black). The triangular boxes represent haploblocks along LG 7D calculated with Haploview software using the four-gamete method.
