# Supplementary material for: Pedigree-Based Analysis in a Multiparental Population of Octoploid Strawberry Reveals QTL Alleles Conferring Resistance to Phytophthora cactorum
Source: G3 (Bethesda). 2017 Jun 5;7(6):1707–19. doi: 10.1534/g3.117.042119 (PMC5473751; doi:10.1534/g3.117.042119)
Supplement: Supplementary file 10 [file 1707FigureS10.pdf]

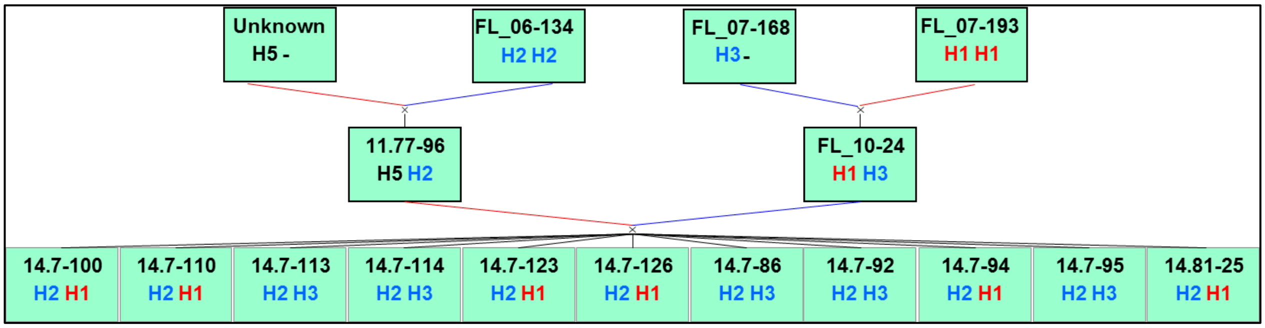

Supplementary Figure S10 Tracing the inheritance of resistant haplotypes H2 and H3 and their inheritance in a pedigree from the UF strawberry breeding program. SNP marker data is not available for selections prior to 2006, thus the origins of the haplotypes cannot be traced further with confidence
