# Supplementary material for: Pedigree-Based Analysis in a Multiparental Population of Octoploid Strawberry Reveals QTL Alleles Conferring Resistance to Phytophthora cactorum
Source: G3 (Bethesda). 2017 Jun 5;7(6):1707–19. doi: 10.1534/g3.117.042119 (PMC5473751; doi:10.1534/g3.117.042119)
Supplement: Supplementary file 11 [file 1707FigureS11.pdf]

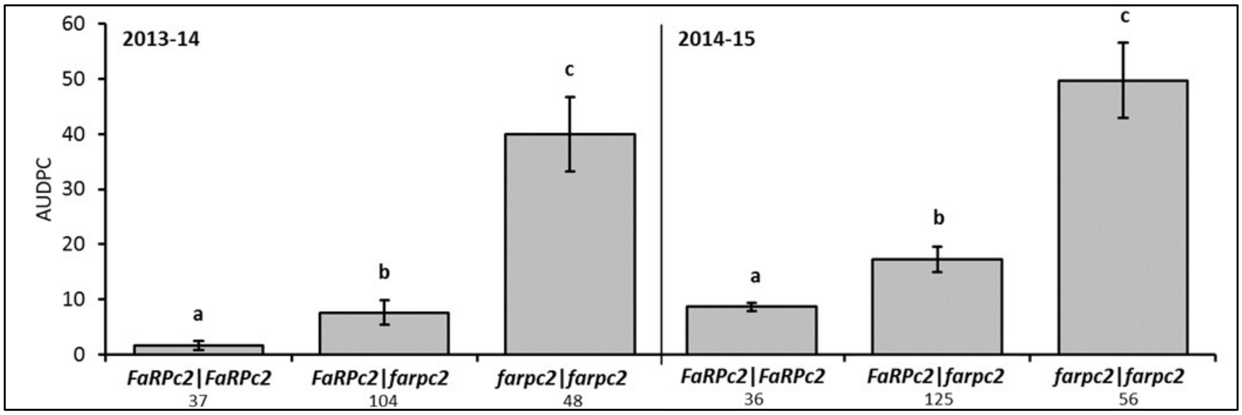

Supplementary Figure S11 Mean area under the disease progress curve (AUDPC) effects for three QTL genotypes of the *FaRPc2* locus under a simple, bi-allelic QTL model for resistance to *Phytophthora cactorum* for QTL validation sets evaluated during the 2013-14 and 2014-15 seasons. Different letters next to each bar within seasons represent statistically significant differences (P<0.05), as determined by the Steele-Dwass non-parametric multiple comparison test. Standard error bars were generated from a GLM analysis to illustrate variation around the means. The number of individuals having each QTL genotype are shown below the genotype designations
