# Supplementary material for: Pedigree-Based Analysis in a Multiparental Population of Octoploid Strawberry Reveals QTL Alleles Conferring Resistance to Phytophthora cactorum
Source: G3 (Bethesda). 2017 Jun 5;7(6):1707–19. doi: 10.1534/g3.117.042119 (PMC5473751; doi:10.1534/g3.117.042119)
Supplement: Supplementary file 18 [file 1707FileS7.zip › File S7/2 SAS-analysis/Diplotype effect analysis/output/2013-14_Discocery-RESULTS.docx]

| The SAS System |
| --- |

The NPAR1WAY Procedure

| **Wilcoxon Scores (Rank Sums) for Variable AUDPC Classified by Variable Diplot** | | | | | |
| --- | --- | --- | --- | --- | --- |
| **Diplot** | **N** | **Sum of Scores** | **Expected Under H0** | **Std Dev Under H0** | **Mean Score** |
| **H1H1** | 90 | 31842.00 | 22680.0 | 1169.80211 | 353.800000 |
| **H1H2** | 137 | 33528.00 | 34524.0 | 1358.67926 | 244.729927 |
| **H1H3** | 121 | 24840.50 | 30492.0 | 1304.48919 | 205.293388 |
| **H1H4** | 29 | 10634.50 | 7308.0 | 711.38432 | 366.706897 |
| **H2H2** | 20 | 4455.50 | 5040.0 | 596.35475 | 222.775000 |
| **H2H3** | 61 | 11189.50 | 15372.0 | 996.30468 | 183.434426 |
| **H2H4** | 22 | 5988.50 | 5544.0 | 624.16584 | 272.204545 |
| **H3H3** | 20 | 3609.00 | 5040.0 | 596.35475 | 180.450000 |
| **H3H4** | 3 | 668.50 | 756.0 | 234.99669 | 222.833333 |
| **Average scores were used for ties.** | | | | | |

| **Kruskal-Wallis Test** | |
| --- | --- |
| **Chi-Square** | 108.1768 |
| **DF** | 8 |
| **Pr > Chi-Square** | <.0001 |


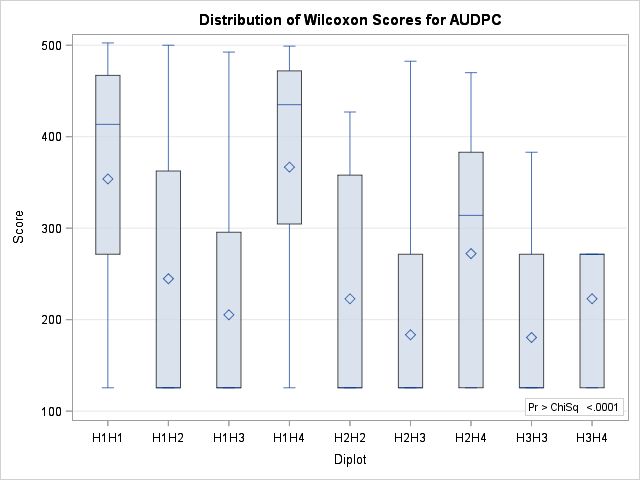


| The SAS System |
| --- |

The NPAR1WAY Procedure

| **Pairwise Two-Sided Multiple Comparison Analysis** | | | |
| --- | --- | --- | --- |
| **Dwass, Steel, Critchlow-Fligner Method** | | | |
| **Variable: AUDPC** | | | |
| **Diplot** | **Wilcoxon Z** | **DSCF Value** | **Pr > DSCF** |
| **H1H1 vs. H1H2** | 6.0315 | 8.5299 | <.0001 |
| **H1H1 vs. H1H3** | 7.5497 | 10.6770 | <.0001 |
| **H1H1 vs. H1H4** | -0.3919 | 0.5542 | 1.0000 |
| **H1H1 vs. H2H2** | 3.6812 | 5.2061 | 0.0072 |
| **H1H1 vs. H2H3** | 6.8241 | 9.6507 | <.0001 |
| **H1H1 vs. H2H4** | 2.7710 | 3.9188 | 0.1240 |
| **H1H1 vs. H3H3** | 4.6350 | 6.5548 | 0.0001 |
| **H1H1 vs. H3H4** | 1.7720 | 2.5060 | 0.7009 |
| **H1H2 vs. H1H3** | 2.6141 | 3.6969 | 0.1805 |
| **H1H2 vs. H1H4** | -4.5248 | 6.3991 | 0.0002 |
| **H1H2 vs. H2H2** | 0.6946 | 0.9823 | 0.9989 |
| **H1H2 vs. H2H3** | 3.2246 | 4.5603 | 0.0343 |
| **H1H2 vs. H2H4** | -0.9431 | 1.3337 | 0.9905 |
| **H1H2 vs. H3H3** | 2.1680 | 3.0660 | 0.4268 |
| **H1H2 vs. H3H4** | 0.3693 | 0.5223 | 1.0000 |
| **H1H3 vs. H1H4** | -5.7165 | 8.0844 | <.0001 |
| **H1H3 vs. H2H2** | -0.6052 | 0.8558 | 0.9996 |
| **H1H3 vs. H2H3** | 1.4057 | 1.9880 | 0.8961 |
| **H1H3 vs. H2H4** | -2.4676 | 3.4897 | 0.2479 |
| **H1H3 vs. H3H3** | 0.9234 | 1.3059 | 0.9917 |
| **H1H3 vs. H3H4** | -0.3712 | 0.5250 | 1.0000 |
| **H1H4 vs. H2H2** | 3.5125 | 4.9674 | 0.0132 |
| **H1H4 vs. H2H3** | 5.6929 | 8.0509 | <.0001 |
| **H1H4 vs. H2H4** | 2.6430 | 3.7377 | 0.1689 |
| **H1H4 vs. H3H3** | 4.3433 | 6.1424 | 0.0005 |
| **H1H4 vs. H3H4** | 1.8830 | 2.6630 | 0.6256 |
| **H2H2 vs. H2H3** | 1.3506 | 1.9100 | 0.9159 |
| **H2H2 vs. H2H4** | -1.1848 | 1.6756 | 0.9599 |
| **H2H2 vs. H3H3** | 1.0176 | 1.4391 | 0.9843 |
| **H2H2 vs. H3H4** | -0.1010 | 0.1428 | 1.0000 |
| **H2H3 vs. H2H4** | -2.9884 | 4.2263 | 0.0694 |
| **H2H3 vs. H3H3** | -0.0980 | 0.1386 | 1.0000 |
| **H2H3 vs. H3H4** | -1.0425 | 1.4744 | 0.9817 |
| **H2H4 vs. H3H3** | 2.3716 | 3.3539 | 0.2997 |
| **H2H4 vs. H3H4** | 0.8216 | 1.1619 | 0.9963 |
| **H3H3 vs. H3H4** | -0.9781 | 1.3833 | 0.9879 |


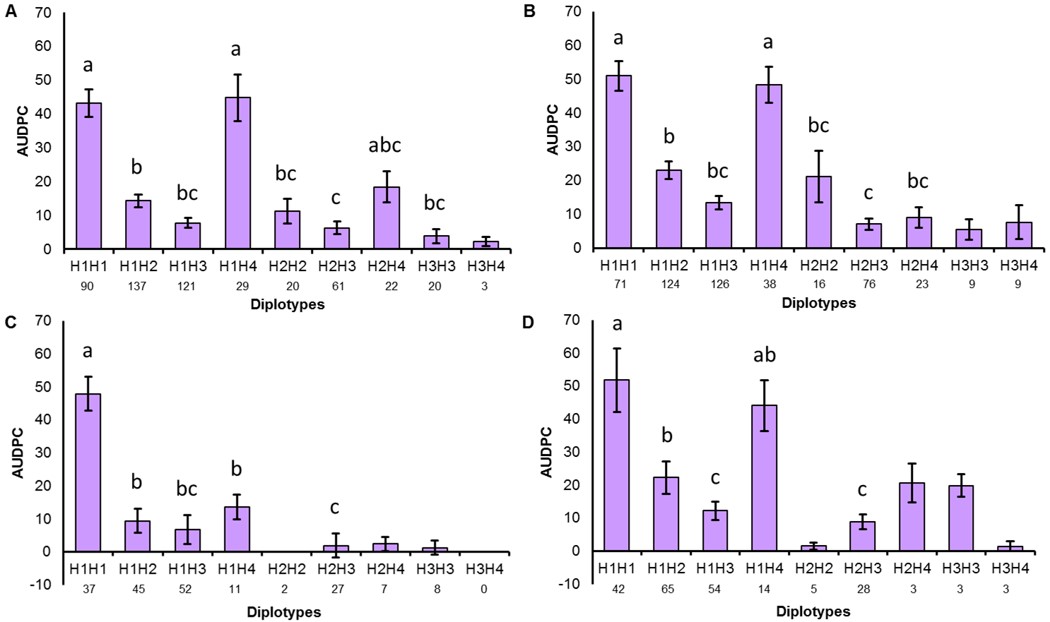


Figure 5 updated.
