# Supplementary material for: Pedigree-Based Analysis in a Multiparental Population of Octoploid Strawberry Reveals QTL Alleles Conferring Resistance to Phytophthora cactorum
Source: G3 (Bethesda). 2017 Jun 5;7(6):1707–19. doi: 10.1534/g3.117.042119 (PMC5473751; doi:10.1534/g3.117.042119)
Supplement: Supplementary file 18 [file 1707FileS7.zip › File S7/2 SAS-analysis/Diplotype effect analysis/output/2013-14_Validation-RESULTS.docx]

| The SAS System |
| --- |

The NPAR1WAY Procedure

| **Wilcoxon Scores (Rank Sums) for Variable AUDPC Classified by Variable Diplot** | | | | | |
| --- | --- | --- | --- | --- | --- |
| **Diplot** | **N** | **Sum of Scores** | **Expected Under H0** | **Std Dev Under H0** | **Mean Score** |
| **H1H1** | 37 | 5794.00 | 3515.0 | 288.541566 | 156.594595 |
| **H1H2** | 45 | 4221.00 | 4275.0 | 309.722839 | 93.800000 |
| **H1H3** | 52 | 4276.50 | 4940.0 | 324.748897 | 82.240385 |
| **H1H4** | 11 | 1189.50 | 1045.0 | 170.251941 | 108.136364 |
| **H2H3** | 27 | 1498.50 | 2565.0 | 254.463278 | 55.500000 |
| **H2H4** | 7 | 462.00 | 665.0 | 137.331588 | 66.000000 |
| **H3H3** | 8 | 436.50 | 760.0 | 146.409754 | 54.562500 |
| **H2H2** | 2 | 77.00 | 190.0 | 74.408325 | 38.500000 |
| **Average scores were used for ties.** | | | | | |

| **Kruskal-Wallis Test** | |
| --- | --- |
| **Chi-Square** | 78.0165 |
| **DF** | 7 |
| **Pr > Chi-Square** | <.0001 |


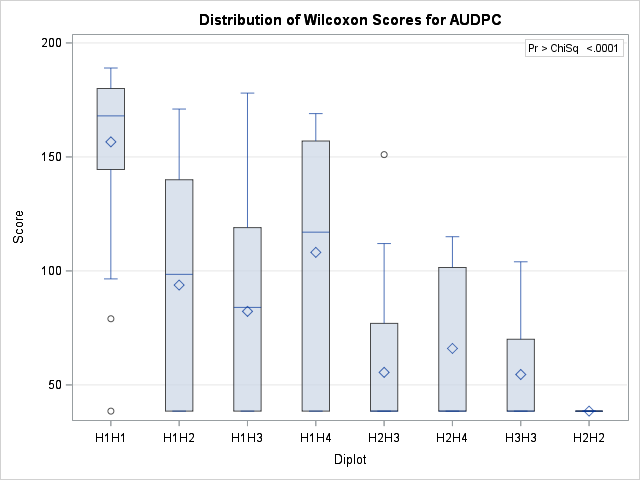


| The SAS System |
| --- |

The NPAR1WAY Procedure

| **Pairwise Two-Sided Multiple Comparison Analysis** | | | |
| --- | --- | --- | --- |
| **Dwass, Steel, Critchlow-Fligner Method** | | | |
| **Variable: AUDPC** | | | |
| **Diplot** | **Wilcoxon Z** | **DSCF Value** | **Pr > DSCF** |
| **H1H1 vs. H1H2** | 5.7240 | 8.0949 | <.0001 |
| **H1H1 vs. H1H3** | 6.5537 | 9.2683 | <.0001 |
| **H1H1 vs. H1H4** | 3.2022 | 4.5286 | 0.0297 |
| **H1H1 vs. H2H3** | 6.4151 | 9.0723 | <.0001 |
| **H1H1 vs. H2H4** | 3.8373 | 5.4267 | 0.0031 |
| **H1H1 vs. H3H3** | 4.1343 | 5.8467 | 0.0009 |
| **H1H1 vs. H2H2** | 2.2928 | 3.2424 | 0.2974 |
| **H1H2 vs. H1H3** | 1.3203 | 1.8672 | 0.8915 |
| **H1H2 vs. H1H4** | -0.9921 | 1.4030 | 0.9757 |
| **H1H2 vs. H2H3** | 3.3342 | 4.7153 | 0.0194 |
| **H1H2 vs. H2H4** | 1.4840 | 2.0987 | 0.8163 |
| **H1H2 vs. H3H3** | 2.0741 | 2.9332 | 0.4318 |
| **H1H2 vs. H2H2** | 1.5270 | 2.1594 | 0.7931 |
| **H1H3 vs. H1H4** | -1.6336 | 2.3102 | 0.7299 |
| **H1H3 vs. H2H3** | 2.7312 | 3.8625 | 0.1132 |
| **H1H3 vs. H2H4** | 0.8628 | 1.2201 | 0.9892 |
| **H1H3 vs. H3H3** | 1.6854 | 2.3835 | 0.6969 |
| **H1H3 vs. H2H2** | 1.3995 | 1.9791 | 0.8579 |
| **H1H4 vs. H2H3** | 3.1554 | 4.4624 | 0.0344 |
| **H1H4 vs. H2H4** | 1.8194 | 2.5730 | 0.6067 |
| **H1H4 vs. H3H3** | 2.3570 | 3.3332 | 0.2629 |
| **H1H4 vs. H2H2** | 1.6243 | 2.2972 | 0.7357 |
| **H2H3 vs. H2H4** | -0.8992 | 1.2716 | 0.9862 |
| **H2H3 vs. H3H3** | 0.1023 | 0.1446 | 1.0000 |
| **H2H3 vs. H2H2** | 0.8024 | 1.1347 | 0.9930 |
| **H2H4 vs. H3H3** | 0.6209 | 0.8780 | 0.9986 |
| **H2H4 vs. H2H2** | 1.0436 | 1.4759 | 0.9677 |
| **H3H3 vs. H2H2** | 0.7454 | 1.0541 | 0.9956 |


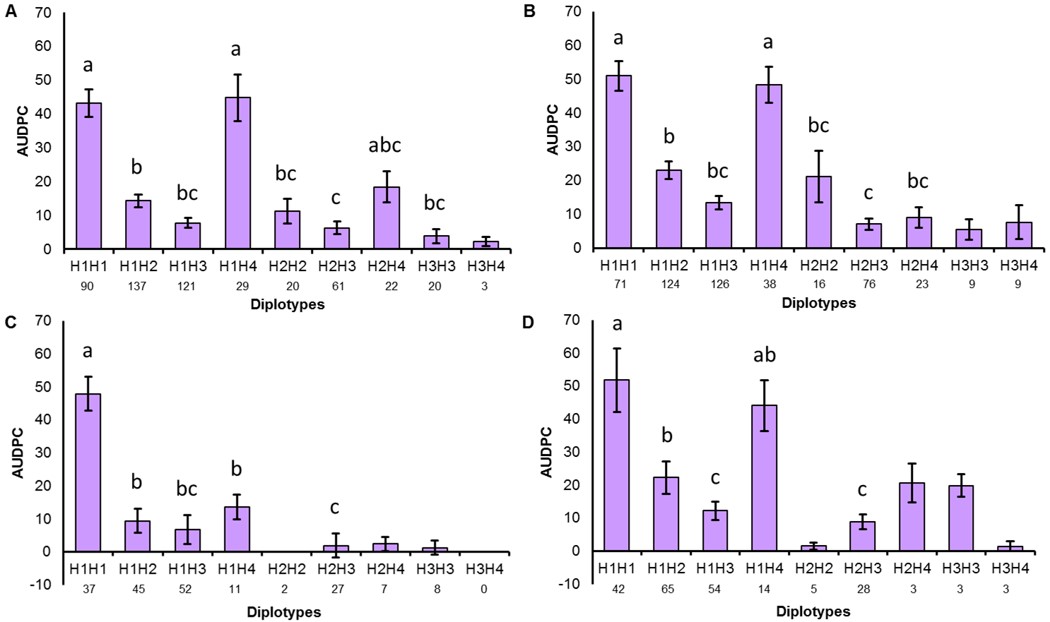


Figure 5 updated.
