# Supplementary material for: Pedigree-Based Analysis in a Multiparental Population of Octoploid Strawberry Reveals QTL Alleles Conferring Resistance to Phytophthora cactorum
Source: G3 (Bethesda). 2017 Jun 5;7(6):1707–19. doi: 10.1534/g3.117.042119 (PMC5473751; doi:10.1534/g3.117.042119)
Supplement: Supplementary file 18 [file 1707FileS7.zip › File S7/2 SAS-analysis/Diplotype effect analysis/output/2014-15_Discovery-RESULTS.docx]

| The SAS System |
| --- |

The NPAR1WAY Procedure

| **Wilcoxon Scores (Rank Sums) for Variable AUDPC Classified by Variable Diplot** | | | | | |
| --- | --- | --- | --- | --- | --- |
| **Diplot** | **N** | **Sum of Scores** | **Expected Under H0** | **Std Dev Under H0** | **Mean Score** |
| **H1H1** | 71 | 25592.00 | 17501.50 | 1044.34118 | 360.450704 |
| **H1H2** | 124 | 30709.50 | 30566.00 | 1290.34824 | 247.657258 |
| **H1H3** | 126 | 26416.50 | 31059.00 | 1297.17329 | 209.654762 |
| **H1H4** | 38 | 13696.00 | 9367.00 | 793.39947 | 360.421053 |
| **H2H2** | 16 | 3735.00 | 3944.00 | 527.15175 | 233.437500 |
| **H2H3** | 76 | 13280.00 | 18734.00 | 1074.05283 | 174.736842 |
| **H2H4** | 23 | 4497.00 | 5669.50 | 627.36823 | 195.521739 |
| **H3H3** | 9 | 1581.00 | 2218.50 | 398.26029 | 175.666667 |
| **H3H4** | 9 | 1771.00 | 2218.50 | 398.26029 | 196.777778 |
| **Average scores were used for ties.** | | | | | |

| **Kruskal-Wallis Test** | |
| --- | --- |
| **Chi-Square** | 117.4033 |
| **DF** | 8 |
| **Pr > Chi-Square** | <.0001 |


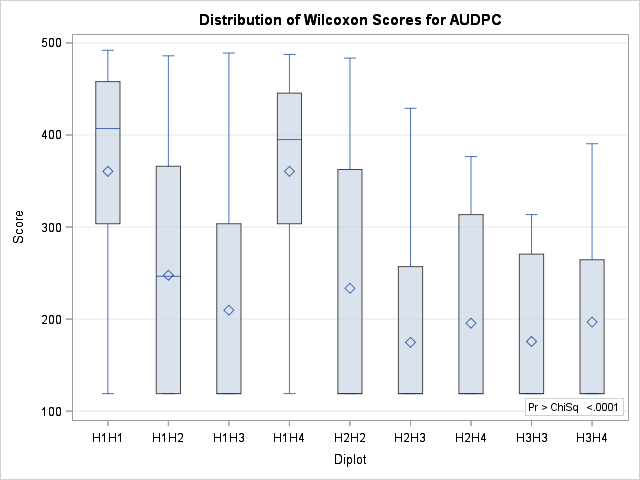


| The SAS System |
| --- |

The NPAR1WAY Procedure

| **Pairwise Two-Sided Multiple Comparison Analysis** | | | |
| --- | --- | --- | --- |
| **Dwass, Steel, Critchlow-Fligner Method** | | | |
| **Variable: AUDPC** | | | |
| **Diplot** | **Wilcoxon Z** | **DSCF Value** | **Pr > DSCF** |
| **H1H1 vs. H1H2** | 5.4415 | 7.6954 | <.0001 |
| **H1H1 vs. H1H3** | 7.4999 | 10.6065 | <.0001 |
| **H1H1 vs. H1H4** | 0.2770 | 0.3917 | 1.0000 |
| **H1H1 vs. H2H2** | 3.0786 | 4.3537 | 0.0535 |
| **H1H1 vs. H2H3** | 7.9434 | 11.2337 | <.0001 |
| **H1H1 vs. H2H4** | 4.9449 | 6.9932 | <.0001 |
| **H1H1 vs. H3H3** | 3.6693 | 5.1892 | 0.0075 |
| **H1H1 vs. H3H4** | 3.4375 | 4.8614 | 0.0171 |
| **H1H2 vs. H1H3** | 2.3252 | 3.2883 | 0.3267 |
| **H1H2 vs. H1H4** | -4.4655 | 6.3151 | 0.0003 |
| **H1H2 vs. H2H2** | 0.3770 | 0.5332 | 1.0000 |
| **H1H2 vs. H2H3** | 3.8878 | 5.4982 | 0.0032 |
| **H1H2 vs. H2H4** | 1.7213 | 2.4343 | 0.7335 |
| **H1H2 vs. H3H3** | 1.5825 | 2.2380 | 0.8147 |
| **H1H2 vs. H3H4** | 1.0962 | 1.5503 | 0.9749 |
| **H1H3 vs. H1H4** | -6.1745 | 8.7320 | <.0001 |
| **H1H3 vs. H2H2** | -0.6857 | 0.9698 | 0.9990 |
| **H1H3 vs. H2H3** | 2.1683 | 3.0664 | 0.4266 |
| **H1H3 vs. H2H4** | 0.4491 | 0.6352 | 1.0000 |
| **H1H3 vs. H3H3** | 0.8188 | 1.1580 | 0.9964 |
| **H1H3 vs. H3H4** | 0.3240 | 0.4582 | 1.0000 |
| **H1H4 vs. H2H2** | 3.1064 | 4.3931 | 0.0493 |
| **H1H4 vs. H2H3** | 6.9529 | 9.8328 | <.0001 |
| **H1H4 vs. H2H4** | 4.6779 | 6.6156 | 0.0001 |
| **H1H4 vs. H3H3** | 3.4839 | 4.9269 | 0.0146 |
| **H1H4 vs. H3H4** | 3.1116 | 4.4005 | 0.0486 |
| **H2H2 vs. H2H3** | 1.7455 | 2.4685 | 0.7181 |
| **H2H2 vs. H2H4** | 0.8966 | 1.2680 | 0.9932 |
| **H2H2 vs. H3H3** | 1.0865 | 1.5366 | 0.9763 |
| **H2H2 vs. H3H4** | 0.5298 | 0.7493 | 0.9998 |
| **H2H3 vs. H2H4** | -0.9322 | 1.3184 | 0.9912 |
| **H2H3 vs. H3H3** | -0.1619 | 0.2290 | 1.0000 |
| **H2H3 vs. H3H4** | -0.7894 | 1.1164 | 0.9972 |
| **H2H4 vs. H3H3** | 0.5305 | 0.7502 | 0.9998 |
| **H2H4 vs. H3H4** | -0.0943 | 0.1333 | 1.0000 |
| **H3H3 vs. H3H4** | -0.2010 | 0.2843 | 1.0000 |
