# Supplementary material for: Pedigree-Based Analysis in a Multiparental Population of Octoploid Strawberry Reveals QTL Alleles Conferring Resistance to Phytophthora cactorum
Source: G3 (Bethesda). 2017 Jun 5;7(6):1707–19. doi: 10.1534/g3.117.042119 (PMC5473751; doi:10.1534/g3.117.042119)
Supplement: Supplementary file 18 [file 1707FileS7.zip › File S7/2 SAS-analysis/Diplotype effect analysis/output/2014-15_Validation-RESULTS.docx]

| The SAS System |
| --- |

The NPAR1WAY Procedure

| **Wilcoxon Scores (Rank Sums) for Variable AUDPC Classified by Variable Diplot** | | | | | |
| --- | --- | --- | --- | --- | --- |
| **Diplot** | **N** | **Sum of Scores** | **Expected Under H0** | **Std Dev Under H0** | **Mean Score** |
| **H2H2** | 5 | 160.00 | 545.0 | 138.748305 | 32.000000 |
| **H2H3** | 28 | 1922.50 | 3052.0 | 310.016402 | 68.660714 |
| **H3H3** | 3 | 207.00 | 327.0 | 107.979738 | 69.000000 |
| **H1H2** | 65 | 7483.00 | 7085.0 | 423.597341 | 115.123077 |
| **H1H3** | 54 | 4169.00 | 5886.0 | 399.820848 | 77.203704 |
| **H1H4** | 14 | 2198.00 | 1526.0 | 227.188733 | 157.000000 |
| **H3H4** | 3 | 115.00 | 327.0 | 107.979738 | 38.333333 |
| **H2H4** | 3 | 363.00 | 327.0 | 107.979738 | 121.000000 |
| **H1H1** | 42 | 7035.50 | 4578.0 | 365.357833 | 167.511905 |
| **Average scores were used for ties.** | | | | | |

| **Kruskal-Wallis Test** | |
| --- | --- |
| **Chi-Square** | 83.3544 |
| **DF** | 8 |
| **Pr > Chi-Square** | <.0001 |


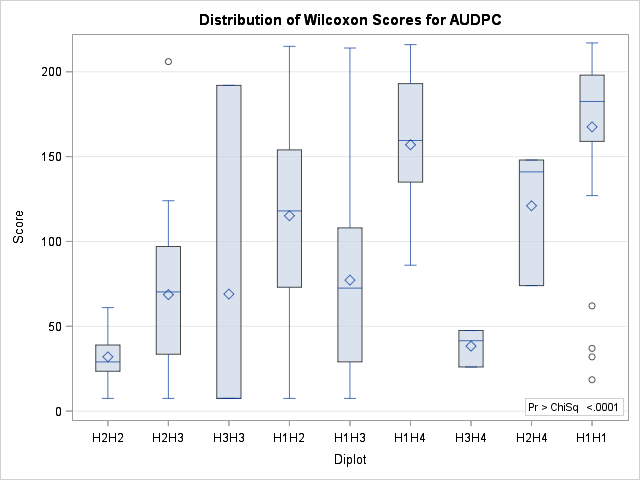


| The SAS System |
| --- |

The NPAR1WAY Procedure

| **Pairwise Two-Sided Multiple Comparison Analysis** | | | |
| --- | --- | --- | --- |
| **Dwass, Steel, Critchlow-Fligner Method** | | | |
| **Variable: AUDPC** | | | |
| **Diplot** | **Wilcoxon Z** | **DSCF Value** | **Pr > DSCF** |
| **H2H2 vs. H2H3** | -1.9097 | 2.7008 | 0.6070 |
| **H2H2 vs. H3H3** | 0.4583 | 0.6481 | 0.9999 |
| **H2H2 vs. H1H2** | -3.1131 | 4.4026 | 0.0483 |
| **H2H2 vs. H1H3** | -1.9066 | 2.6963 | 0.6092 |
| **H2H2 vs. H1H4** | -3.2404 | 4.5826 | 0.0327 |
| **H2H2 vs. H3H4** | -0.7454 | 1.0541 | 0.9981 |
| **H2H2 vs. H2H4** | -2.2361 | 3.1623 | 0.3820 |
| **H2H2 vs. H1H1** | -3.3468 | 4.7331 | 0.0232 |
| **H2H3 vs. H3H3** | 0.8034 | 1.1362 | 0.9968 |
| **H2H3 vs. H1H2** | -3.9997 | 5.6564 | 0.0021 |
| **H2H3 vs. H1H3** | -0.5185 | 0.7333 | 0.9999 |
| **H2H3 vs. H1H4** | -4.4299 | 6.2648 | 0.0003 |
| **H2H3 vs. H3H4** | 1.3036 | 1.8435 | 0.9306 |
| **H2H3 vs. H2H4** | -1.8047 | 2.5523 | 0.6792 |
| **H2H3 vs. H1H1** | -5.7007 | 8.0620 | <.0001 |
| **H3H3 vs. H1H2** | -0.9708 | 1.3729 | 0.9885 |
| **H3H3 vs. H1H3** | -0.8586 | 1.2142 | 0.9949 |
| **H3H3 vs. H1H4** | -1.3867 | 1.9611 | 0.9032 |
| **H3H3 vs. H3H4** | -0.6642 | 0.9393 | 0.9992 |
| **H3H3 vs. H2H4** | -0.6642 | 0.9393 | 0.9992 |
| **H3H3 vs. H1H1** | -1.6381 | 2.3166 | 0.7838 |
| **H1H2 vs. H1H3** | 3.8304 | 5.4170 | 0.0041 |
| **H1H2 vs. H1H4** | -2.6320 | 3.7223 | 0.1732 |
| **H1H2 vs. H3H4** | 2.4340 | 3.4422 | 0.2653 |
| **H1H2 vs. H2H4** | -0.2837 | 0.4012 | 1.0000 |
| **H1H2 vs. H1H1** | -5.3143 | 7.5156 | <.0001 |
| **H1H3 vs. H1H4** | -4.3086 | 6.0933 | 0.0006 |
| **H1H3 vs. H3H4** | 1.1977 | 1.6939 | 0.9572 |
| **H1H3 vs. H2H4** | -1.5016 | 2.1236 | 0.8553 |
| **H1H3 vs. H1H1** | -6.3078 | 8.9206 | <.0001 |
| **H1H4 vs. H3H4** | 2.6458 | 3.7417 | 0.1678 |
| **H1H4 vs. H2H4** | 1.5119 | 2.1381 | 0.8504 |
| **H1H4 vs. H1H1** | -1.1732 | 1.6591 | 0.9622 |
| **H3H4 vs. H2H4** | -1.9640 | 2.7775 | 0.5688 |
| **H3H4 vs. H1H1** | -2.5481 | 3.6035 | 0.2091 |
| **H2H4 vs. H1H1** | -2.0021 | 2.8314 | 0.5418 |
