# Supplementary material for: Pedigree-Based Analysis in a Multiparental Population of Octoploid Strawberry Reveals QTL Alleles Conferring Resistance to Phytophthora cactorum
Source: G3 (Bethesda). 2017 Jun 5;7(6):1707–19. doi: 10.1534/g3.117.042119 (PMC5473751; doi:10.1534/g3.117.042119)
Supplement: Supplementary file 18 [file 1707FileS7.zip › File S7/2 SAS-analysis/QTL-genotype effect analysis/output/2013-14 Discovery - QTL-genotype analysis SAS results.docx]

| The SAS System |
| --- |

The GLM Procedure

| **Class Level Information** | | |
| --- | --- | --- |
| **Class** | **Levels** | **Values** |
| **ID** | 488 | 13.1-146 13.1-16 13.1-161 13.1-18 13.1-187 13.1-25 13.1-48 13.1-49 13.1-73 13.1-74 13.10-12 13.10-24 13.10-3 13.10-45 13.12-1 13.12-42 13.12-48 13.12-61 13.12-65 13.12-71 13.12-75 13.12-79 13.13-23 13.13-36 13.13-56 13.13-68 13.13-71 13.13-83 13.14-10 13.14-11 13.14-19 13.14-23 13.14-28 13.14-31 13.14-33 13.14-34 13.14-36 13.14-62 13.14-7 13.17-12 13.17-16 13.17-17 13.17-60 13.17-65 13.17-66 13.17-72 13.17-78 13.18-20 13.18-37 13.18-49 13.18-90 13.19-1 13.19-16 13.19-17 13.19-37 13.19-56 13.19-63 13.19-64 13.19-66 13.19-7 13.2-1 13.2-109 13.2-118 13.2-18 13.2-25 13.2-4 13.2-68 13.2-84 13.2-94 13.2-97 13.20-10 13.20-15 13.20-16 13.20-19 13.20-24 13.20-31 13.20-78 13.20-88 13.21-11 13.21-30 13.21-32 13.21-63 13.21-68 13.21-73 13.21-90 13.22-15 13.22-24 13.22-28 13.22-29 13.22-30 13.22-31 13.22-55 13.22-6 13.23-18 13.23-21 13.23-3 13.23-35 13.23-38 13.23-52 13.23-6 13.23-69 13.23-71 13.23-75 13.23-85 13.23-9 13.24-12 13.24-13 13.24-16 13.24-18 13.24-53 13.24-61 13.24-66 13.25-33 13.25-57 13.25-60 13.25-63 13.25-65 13.25-66 13.25-68 13.25-70 13.25-71 13.26-11 13.26-14 13.26-17 13.26-3 13.26-39 13.26-47 13.26-55 13.26-65 13.27-10 13.27-12 13.27-16 13.27-17 13.27-18 13.27-19 13.27-20 13.27-9 13.27-98 13.28-10 13.28-11 13.28-17 13.28-18 13.28-36 13.28-42 13.28-43 13.28-44 13.28-45 13.28-49 13.28-56 13.29-10 13.29-13 13.29-14 13.29-16 13.29-18 13.29-4 13.29-48 13.29-88 13.3-1 13.3-104 13.3-106 13.3-126 13.3-19 13.3-22 13.3-65 13.3-68 13.3-84 13.30-11 13.30-14 13.30-18 13.30-22 13.30-5 13.30-65 13.30-7 13.30-72 13.30-78 13.30-86 13.31-1 13.31-10 13.31-11 13.31-18 13.31-2 13.31-44 13.31-55 13.31-6 13.31-77 13.31-82 13.31-9 13.32-1 13.32-16 13.32-17 13.32-2 13.32-20 13.32-6 13.32-7 13.32-9 13.33-11 13.33-12 13.33-13 13.33-14 13.33-31 13.33-4 13.33-42 13.34-16 13.34-17 13.34-18 13.34-25 13.34-35 13.34-41 13.34-44 13.34-55 13.34-64 13.34-66 13.35-1 13.35-10 13.35-11 13.35-16 13.35-18 13.35-2 13.35-23 13.35-25 13.35-37 13.35-98 13.36-19 13.36-22 13.36-46 13.36-56 13.36-57 13.36-67 13.36-86 13.37-15 13.37-19 13.37-20 13.37-21 13.37-25 13.37-30 13.37-39 13.37-51 13.38-11 13.38-12 13.38-15 13.38-17 13.38-18 13.38-36 13.38-59 13.38-63 13.38-68 13.39-12 13.39-14 13.4-1 13.4-100 13.4-107 13.4-115 13.4-120 13.4-131 13.4-62 13.4-88 13.4-9 13.40-10 13.40-24 13.40-61 13.40-71 13.40-76 13.41-10 13.41-11 13.41-12 13.41-13 13.41-61 13.41-93 13.41-96 13.42-11 13.42-12 13.42-39 13.42-75 13.42-77 13.42-91 13.42-98 13.43-16 13.43-42 13.43-63 13.43-66 13.43-67 13.43-91 13.43-95 13.44-10 13.44-11 13.44-14 13.44-16 13.44-25 13.44-51 13.44-8 13.44-9 13.45-30 13.45-54 13.45-60 13.45-65 13.45-71 13.45-75 13.45-86 13.45-96 13.45-97 13.46-17 13.46-42 13.46-43 13.46-85 13.47-11 13.47-23 13.47-3 13.47-4 13.47-45 13.47-83 13.47-87 13.48-10 13.48-12 13.48-19 13.48-44 13.48-49 13.48-53 13.48-54 13.48-70 13.48-88 13.49-11 13.49-12 13.49-31 13.49-48 13.49-49 13.49-61 13.49-67 13.49-84 13.49-90 13.5-124 13.5-125 13.5-19 13.5-21 13.5-36 13.5-56 13.5-59 13.5-63 13.50-13 13.50-14 13.50-57 13.50-59 13.50-67 13.50-74 13.50-76 13.50-79 13.50-99 13.51-12 13.51-22 13.51-23 13.51-25 13.51-34 13.51-38 13.51-57 13.51-73 13.51-8 13.52-13 13.52-15 13.52-18 13.52-27 13.52-5 13.52-50 13.52-51 13.52-57 13.52-60 13.52-7 13.52-9 13.53-13 13.53-15 13.53-23 13.53-24 13.53-27 13.53-28 13.53-4 13.53-43 13.53-87 13.54-13 13.54-16 13.54-18 13.54-25 13.54-54 13.54-57 13.55-10 13.55-12 13.55-16 13.55-37 13.55-40 13.55-43 13.55-50 13.55-83 13.55-84 13.55-94 13.56-24 13.56-29 13.56-32 13.56-35 13.56-40 13.56-53 13.56-59 13.56-61 13.56-64 13.56-69 13.6-113 13.6-185 13.6-186 13.6-48 13.6-58 13.6-69 13.6-75 13.6-8 13.6-80 13.6-97 13.60-65 13.60-79 13.61-54 13.61-56 13.61-59 13.61-65 13.61-71 13.61-75 13.61-79 13.62-10 13.62-11 13.62-12 13.62-15 13.62-44 13.62-51 13.63-12 13.63-13 13.63-38 13.63-44 13.63-47 13.63-49 13.63-51 13.63-53 13.63-56 13.64-11 13.64-63 13.64-70 13.64-99 13.65-16 13.65-20 13.65-26 13.65-32 13.65-40 13.65-46 13.65-61 13.65-69 13.66-14 13.66-30 13.66-59 13.66-70 13.66-84 13.67-10 13.67-15 13.67-37 13.67-4 13.67-46 13.67-7 13.68-23 13.68-24 13.68-25 13.68-47 13.68-9 13.7-125 13.7-13 13.7-156 13.7-162 13.7-2 13.7-20 13.7-3 13.7-45 13.7-70 13.8-1 13.8-103 13.8-21 13.8-46 13.8-63 13.8-83 13.9-1 13.9-12 13.9-14 13.9-17 13.9-2 13.9-33 13.9-41 13.9-42 13.9-5 13.9-7 |
| **Female** | 22 | Dummy10F Dummy24 Dummy40 Dummy46 Dummy62 FL_06-134 FL_06-89 FL_07-102 FL_07-193 FL_08-10 FL_09-134 FL_09-46 FL_09-57 FL_10-153 FL_10-24 FL_10-47 FL_10-51 FL_10-94 FL_10-97 Florida12 WinterDaw Wintersta |
| **Male** | 30 | 11.28-34 Dummy10M Dummy13 Dummy18 Dummy35 Dummy38 Dummy39 Dummy42 Dummy54 Dummy56 Dummy67 FL_06-134 FL_06-38 FL_06-89 FL_07-102 FL_07-193 FL_08-10 FL_09-134 FL_09-46 FL_09-57 FL_10-153 FL_10-24 FL_10-47 FL_10-51 FL_10-64 FL_10-94 FL_10-97 Florida12 WinterDaw Wintersta |
| **Alle1** | 2 | Pc2 pc2 |
| **Alle2** | 2 | Pc2 pc2 |
| **Diplo** | 3 | Pc2_Pc2 Pc2_pc2 pc2_pc2 |
| **AUDPC** | 110 | 0 7 14 21 28 30 37 44 51 53 58 65 66 67 88 89 111 118 3.5 8.2 9.3 10.5 15.2 16.3 17.5 19.8 23.3 24.2 25.5 25.7 26.5 26.8 28.8 30.3 31.2 33.5 35.8 38.2 39.3 40.5 40.8 41.7 42.8 47.5 48.5 48.7 50.7 52.2 56.5 60.2 63.8 67.3 70.5 72.8 77.5 78.7 84.5 85.7 91.2 91.5 92.7 102.8 119.2 128.5 46.75 60.75 64.25 103.1666667 112.1666667 116.8333333 12.83333333 122.6666667 15.16666667 19.83333333 23.33333333 24.16666667 25.66666667 27.66666667 28.83333333 31.16666667 32.33333333 34.66666667 35.83333333 37.33333333 38.16666667 39.33333333 40.83333333 41.66666667 41.83333333 42.83333333 45.16666667 46.16666667 46.33333333 5.833333333 53.33333333 55.33333333 65.83333333 68.16666667 71.66666667 72.83333333 76.33333333 79.83333333 8.166666667 82.16666667 83.33333333 86.83333333 89.16666667 90.33333333 92.66666667 97.33333333 |
| **Outl** | 2 | 0 1 |

| **Number of Observations Read** | 517 |
| --- | --- |
| **Number of Observations Used** | 517 |

| The SAS System |
| --- |

The GLM Procedure

Dependent Variable: AUDPC

| **Source** | **DF** | **Sum of Squares** | **Mean Square** | **F Value** | **Pr > F** |
| --- | --- | --- | --- | --- | --- |
| **Model** | 2 | 101823.2102 | 50911.6051 | 87.11 | <.0001 |
| **Error** | 514 | 300414.8850 | 584.4648 |  |  |
| **Corrected Total** | 516 | 402238.0952 |  |  |  |

| **R-Square** | **Coeff Var** | **Root MSE** | **AUDPC Mean** |
| --- | --- | --- | --- |
| 0.253142 | 132.1358 | 24.17571 | 18.29610 |

| **Source** | **DF** | **Type I SS** | **Mean Square** | **F Value** | **Pr > F** |
| --- | --- | --- | --- | --- | --- |
| **Diplo** | 2 | 101823.2102 | 50911.6051 | 87.11 | <.0001 |

| **Source** | **DF** | **Type III SS** | **Mean Square** | **F Value** | **Pr > F** |
| --- | --- | --- | --- | --- | --- |
| **Diplo** | 2 | 101823.2102 | 50911.6051 | 87.11 | <.0001 |


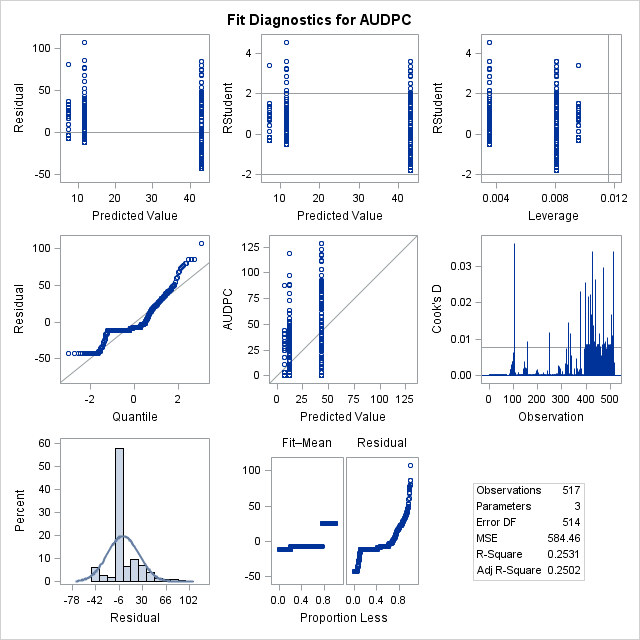


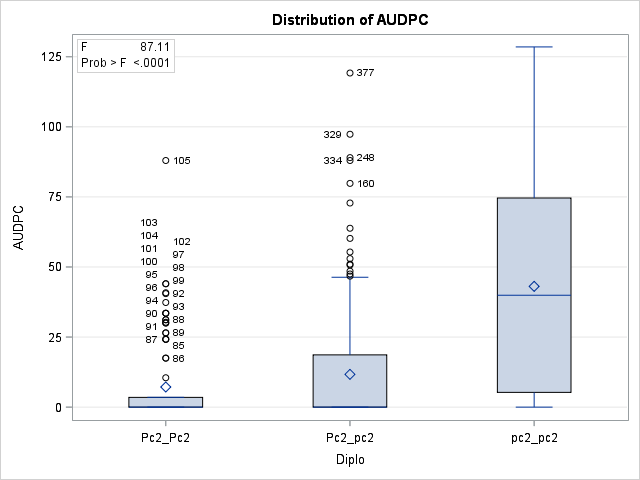


| The SAS System |
| --- |

The GLM Procedure


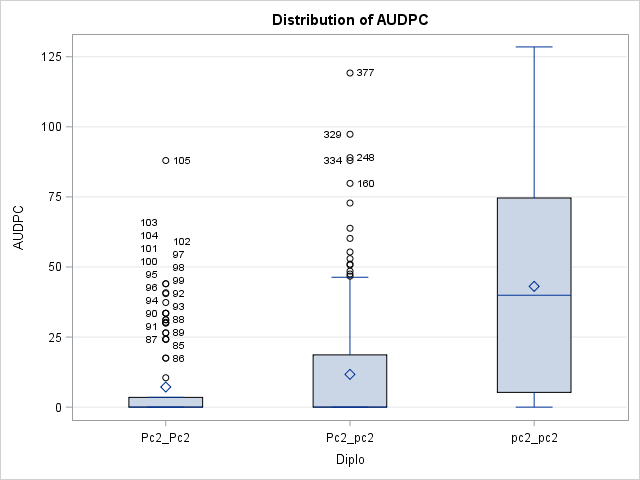


| The SAS System |
| --- |

The GLM Procedure

t Tests (LSD) for AUDPC

| Note: | This test controls the Type I comparisonwise error rate, not the experimentwise error rate. |
| --- | --- |

| **Alpha** | 0.05 |
| --- | --- |
| **Error Degrees of Freedom** | 514 |
| **Error Mean Square** | 584.4648 |
| **Critical Value of t** | 1.96459 |
| **Least Significant Difference** | 5.6278 |
| **Harmonic Mean of Cell Sizes** | 142.4464 |

| Note: | Cell sizes are not equal. |
| --- | --- |

| **Means with the same letter are not significantly different.** | | | |
| --- | --- | --- | --- |
| **t Grouping** | **Mean** | **N** | **Diplo** |
| A | 43.089 | 124 | pc2_pc2 |
|  |  |  |  |
| B | 11.674 | 288 | Pc2_pc2 |
| B |  |  |  |
| B | 7.179 | 105 | Pc2_Pc2 |

| The SAS System |
| --- |

The UNIVARIATE Procedure

Variable: resid

| **Moments** | | | |
| --- | --- | --- | --- |
| **N** | 517 | **Sum Weights** | 517 |
| **Mean** | 0 | **Sum Observations** | 0 |
| **Std Deviation** | 24.1288083 | **Variance** | 582.199389 |
| **Skewness** | 1.12410988 | **Kurtosis** | 2.06517558 |
| **Uncorrected SS** | 300414.885 | **Corrected SS** | 300414.885 |
| **Coeff Variation** | . | **Std Error Mean** | 1.06118378 |

| **Basic Statistical Measures** | | | |
| --- | --- | --- | --- |
| **Location** | | **Variability** | |
| **Mean** | 0.0000 | **Std Deviation** | 24.12881 |
| **Median** | -7.1794 | **Variance** | 582.19939 |
| **Mode** | -11.6745 | **Range** | 150.61423 |
|  |  | **Interquartile Range** | 23.33333 |

| **Tests for Location: Mu0=0** | | | | |
| --- | --- | --- | --- | --- |
| **Test** | **Statistic** | | **p Value** | |
| **Student's t** | **t** | 0 | **Pr > \|t\|** | 1.0000 |
| **Sign** | **M** | -94.5 | **Pr >= \|M\|** | <.0001 |
| **Signed Rank** | **S** | -10110.5 | **Pr >= \|S\|** | 0.0027 |

| **Tests for Normality** | | | | |
| --- | --- | --- | --- | --- |
| **Test** | **Statistic** | | **p Value** | |
| **Shapiro-Wilk** | **W** | 0.869183 | **Pr < W** | <0.0001 |
| **Kolmogorov-Smirnov** | **D** | 0.22239 | **Pr > D** | <0.0100 |
| **Cramer-von Mises** | **W-Sq** | 6.055051 | **Pr > W-Sq** | <0.0050 |
| **Anderson-Darling** | **A-Sq** | 28.66428 | **Pr > A-Sq** | <0.0050 |

| **Quantiles (Definition 5)** | |
| --- | --- |
| **Level** | **Quantile** |
| **100% Max** | 107.52552 |
| **99%** | 79.57796 |
| **95%** | 44.91129 |
| **90%** | 33.24462 |
| **75% Q3** | 11.65885 |
| **50% Median** | -7.17937 |
| **25% Q1** | -11.67448 |
| **10%** | -16.58871 |
| **5%** | -39.58871 |
| **1%** | -43.08871 |
| **0% Min** | -43.08871 |

| **Extreme Observations** | | | |
| --- | --- | --- | --- |
| **Lowest** | | **Highest** | |
| **Value** | **Obs** | **Value** | **Obs** |
| -43.0887 | 514 | 80.8206 | 105 |
| -43.0887 | 503 | 85.4113 | 427 |
| -43.0887 | 502 | 85.4113 | 511 |
| -43.0887 | 500 | 85.6589 | 329 |
| -43.0887 | 498 | 107.5255 | 377 |

The UNIVARIATE Procedure


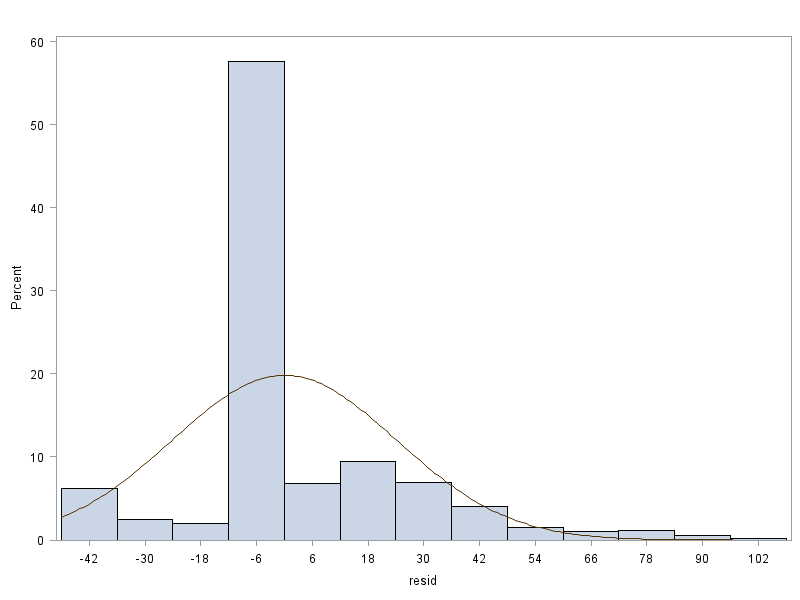


| The SAS System |
| --- |

The UNIVARIATE Procedure

Fitted Normal Distribution for resid

| **Parameters for Normal Distribution** | | |
| --- | --- | --- |
| **Parameter** | **Symbol** | **Estimate** |
| **Mean** | Mu | 0 |
| **Std Dev** | Sigma | 24.12881 |

| **Goodness-of-Fit Tests for Normal Distribution** | | | | |
| --- | --- | --- | --- | --- |
| **Test** | **Statistic** | | **p Value** | |
| **Kolmogorov-Smirnov** | **D** | 0.2223900 | **Pr > D** | <0.010 |
| **Cramer-von Mises** | **W-Sq** | 6.0550513 | **Pr > W-Sq** | <0.005 |
| **Anderson-Darling** | **A-Sq** | 28.6642849 | **Pr > A-Sq** | <0.005 |

| **Quantiles for Normal Distribution** | | |
| --- | --- | --- |
| **Percent** | **Quantile** | |
|  | **Observed** | **Estimated** |
| **1.0** | -43.08871 | -56.1320 |
| **5.0** | -39.58871 | -39.6884 |
| **10.0** | -16.58871 | -30.9223 |
| **25.0** | -11.67448 | -16.2746 |
| **50.0** | -7.17937 | 0.0000 |
| **75.0** | 11.65885 | 16.2746 |
| **90.0** | 33.24462 | 30.9223 |
| **95.0** | 44.91129 | 39.6884 |
| **99.0** | 79.57796 | 56.1320 |
