# Supplementary material for: Pedigree-Based Analysis in a Multiparental Population of Octoploid Strawberry Reveals QTL Alleles Conferring Resistance to Phytophthora cactorum
Source: G3 (Bethesda). 2017 Jun 5;7(6):1707–19. doi: 10.1534/g3.117.042119 (PMC5473751; doi:10.1534/g3.117.042119)
Supplement: Supplementary file 18 [file 1707FileS7.zip › File S7/2 SAS-analysis/QTL-genotype effect analysis/output/2013-14 Validation - QTL-genotype analysis SAS results.docx]

| The SAS System |
| --- |

The GLM Procedure

| **Class Level Information** | | |
| --- | --- | --- |
| **Class** | **Levels** | **Values** |
| **ID** | 189 | 11.105-1 11.107-24 11.107-5 11.108-5 11.116-58 11.116-68 11.121-19 11.13-54 11.139-10 11.20-22 11.21-6 11.23-8 11.31-14 11.31-36 11.31-43 11.31-54 11.34-47 11.34-51 11.40-12 11.40-44 11.43-15 11.66-1 11.83-20 11.85-105 11.85-58 11.88-10 11.93-21 12.10-71 12.11-129 12.11-51 12.115-10 12.119-23 12.119-49 12.12-105 12.12-22 12.121-18 12.121-5 12.125-19 12.125-21 12.13-89 12.15-105 12.15-107 12.15-8 12.17-11 12.17-19 12.17-36 12.17-62 12.18-136 12.18-153 12.18-177 12.18-75 12.19-114 12.19-56 12.19-63 12.20-31 12.21-28 12.23-127 12.23-67 12.23-71 12.24-51 12.24-60 12.25-60 12.26-133 12.26-135 12.26-162 12.26-180 12.26-192 12.26-248 12.26-49 12.27-16 12.27-45 12.27-89 12.28-26 12.28-50 12.28-77 12.30-19 12.30-22 12.31-104 12.32-14 12.32-31 12.32-91 12.33-30 12.34-2 12.35-2 12.35-32 12.35-66 12.35-89 12.37-37 12.39-60 12.4-9 12.43-40 12.45-11 12.45-85 12.47-115 12.47-116 12.47-5 12.47-68 12.5-103 12.5-110 12.5-130 12.5-85 12.5-97 12.50-94 12.52-22 12.54-130 12.54-22 12.54-49 12.54-72 12.54-9 12.55-100 12.55-101 12.55-140 12.55-187 12.55-214 12.55-220 12.55-230 12.55-29 12.55-64 12.55-69 12.55-72 12.55-78 12.55-88 12.56-24 12.57-25 12.59-1 12.59-39 12.6-38 12.60-34 12.61-96 12.62-22 12.62-23 12.67-25 12.68-11 12.68-12 12.70-47 12.70-54 12.70-55 12.70-64 12.71-62 12.74-39 12.75-19 12.75-77 12.76-35 12.76-69 12.77-11 12.77-151 12.77-238 12.77-45 12.77-68 12.77-82 12.82-44 12.85-57 12.86-70 12.87-42 12.88-28 12.88-61 12.88-83 12.88-9 12.89-35 12.89-91 12.9-4 12.90-2 12.90-38 12.90-39 12.90-43 12.90-53 12.90-62 12.91-93 12.92-42 12.93-2 12.93-4 12.94-109 12.94-43 12.94-58 12.94-66 12.94-86 FL_06-38 FL_07-193 FL_09-100 FL_09-46 FL_09-76 FL_10-143 FL_10-163 FL_10-24 FL_10-64 FL_10-72 FL_10-92 Festival Wintersta |
| **Female** | 50 | - 10.133-42 10.133-87 10.133-98 11.143-11 AU_2008-01 AU_2010-11 FL_05-190 FL_06-134 FL_06-38 FL_06-45 FL_06-89 FL_07-102 FL_07-122 FL_07-193 FL_07-68 FL_09-100 FL_09-148 FL_09-150 FL_09-46 FL_09-53 FL_09-57 FL_09-76 FL_09-89 FL_10-131 FL_10-132 FL_10-139 FL_10-15 FL_10-157 FL_10-163 FL_10-175 FL_10-187 FL_10-24 FL_10-47 F_11.116-6 F_11.121-1 F_11.139 F_11.20-22 F_11.21-6 F_11.23-8 F_11.34 F_11.43-15 F_12.25-60 F_12.33 F_12.55-64 F_FL_10-92 Festival Florida127 RosaLinda Winterstar |
| **Male** | 57 | - 11.106-44 11.106-63 11.107-5 11.122-36 11.141-36 11.159-2 11.63-41 11.64-32 11.89-32 FL_04-61 FL_05-131 FL_05-183 FL_06-38 FL_06-89 FL_07-168 FL_07-193 FL_07-68 FL_08-10 FL_08-50 FL_08-78 FL_09-100 FL_09-110 FL_09-134 FL_09-148 FL_09-150 FL_09-76 FL_09-89 FL_10-1 FL_10-131 FL_10-139 FL_10-140 FL_10-15 FL_10-157 FL_10-16 FL_10-163 FL_10-175 FL_10-187 FL_10-19 FL_10-22 FL_10-24 FL_10-47 Festival Florida127 M_11.13 M_11.20-22 M_11.66-1 M_12.10 M_12.121 M_12.26 M_12.31-10 M_12.60-34 M_12.82 M_12.89 M_FL_10-64 OsoGrande Winterstar |
| **Alle1** | 2 | Pc2 pc2 |
| **Alle2** | 2 | Pc2 pc2 |
| **Diplo** | 3 | Pc2_Pc2 Pc2_pc2 pc2_pc2 |
| **AUDPC** | 99 | 0 7 3.5 4.2 4.9 6.3 9.1 9.8 11.9 13.5 15.4 16.8 18.2 18.9 3.85 31.5 36.4 37.1 4.55 43.5 5.25 53.9 7.35 8.05 8.75 0.525 0.875 1.225 1.575 10.15 103.6 14.35 15.75 19.95 2.625 25.55 27.65 29.75 3.325 3.675 4.025 4.375 4.725 6.125 64.75 66.15 79.45 8.075 84.35 95.55 103.25 104.65 11.375 12.075 12.425 12.775 13.125 14.175 18.725 19.425 19.775 20.125 20.475 21.875 23.275 25.375 26.075 31.675 32.025 39.375 47.775 58.975 89.775 10.0625 102.375 121.625 25.4625 30.1875 24.28125 28.13125 29.61875 30.93125 0.972222222 11.76388889 12.01136364 14.58333333 15.16666667 16.04166667 2.916666667 26.61944444 28.09722222 32.66666667 47.48333333 6.805555556 63.02777778 71.59444444 8.166666667 8.458333333 83.14444444 |
| **Outl** | 2 | 0 1 |

| **Number of Observations Read** | 189 |
| --- | --- |
| **Number of Observations Used** | 189 |

| The SAS System |
| --- |

The GLM Procedure

Dependent Variable: AUDPC

| **Source** | **DF** | **Sum of Squares** | **Mean Square** | **F Value** | **Pr > F** |
| --- | --- | --- | --- | --- | --- |
| **Model** | 2 | 42317.4198 | 21158.7099 | 56.10 | <.0001 |
| **Error** | 186 | 70145.6523 | 377.1272 |  |  |
| **Corrected Total** | 188 | 112463.0722 |  |  |  |

| **R-Square** | **Coeff Var** | **Root MSE** | **AUDPC Mean** |
| --- | --- | --- | --- |
| 0.376278 | 132.3236 | 19.41976 | 14.67596 |

| **Source** | **DF** | **Type I SS** | **Mean Square** | **F Value** | **Pr > F** |
| --- | --- | --- | --- | --- | --- |
| **Diplo** | 2 | 42317.41984 | 21158.70992 | 56.10 | <.0001 |

| **Source** | **DF** | **Type III SS** | **Mean Square** | **F Value** | **Pr > F** |
| --- | --- | --- | --- | --- | --- |
| **Diplo** | 2 | 42317.41984 | 21158.70992 | 56.10 | <.0001 |


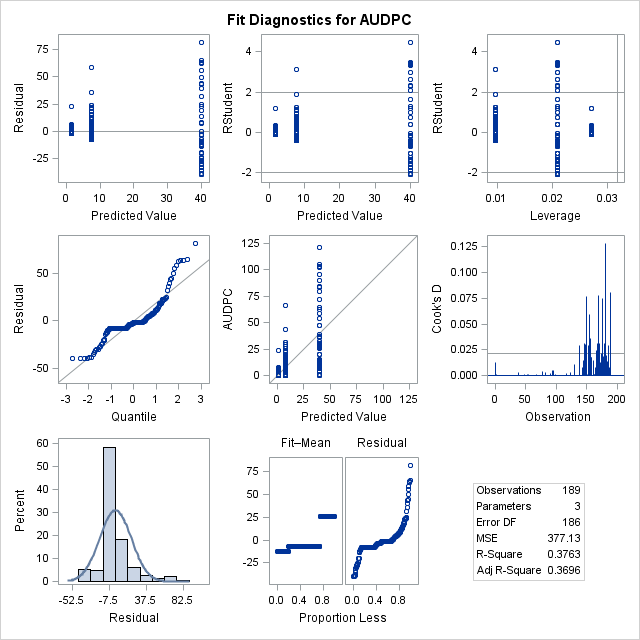


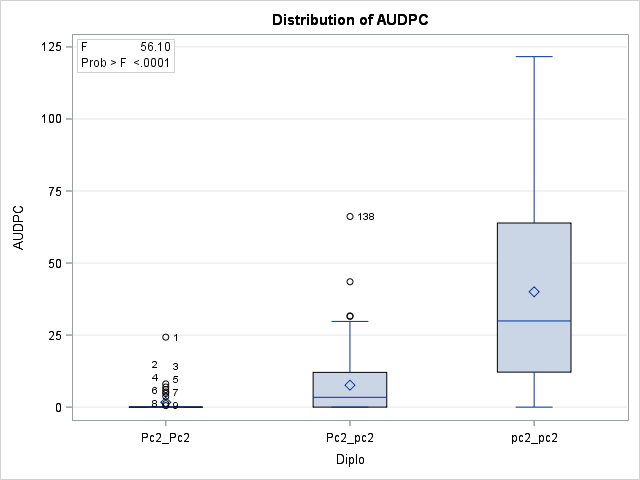


| The SAS System |
| --- |

The GLM Procedure


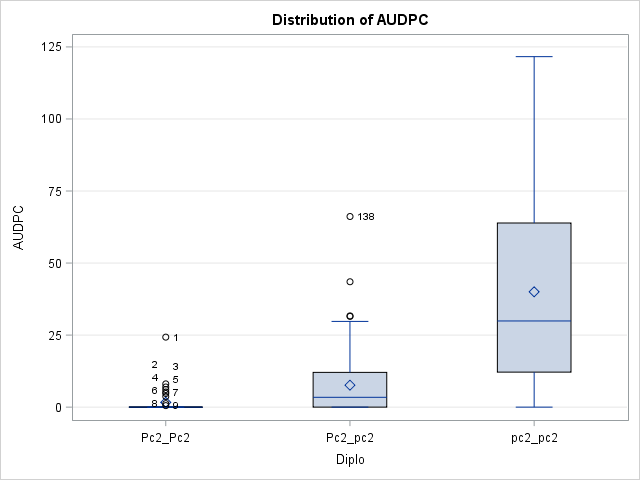


| The SAS System |
| --- |

The GLM Procedure

t Tests (LSD) for AUDPC

| Note: | This test controls the Type I comparisonwise error rate, not the experimentwise error rate. |
| --- | --- |

| **Alpha** | 0.05 |
| --- | --- |
| **Error Degrees of Freedom** | 186 |
| **Error Mean Square** | 377.1272 |
| **Critical Value of t** | 1.97280 |
| **Least Significant Difference** | 7.4994 |
| **Harmonic Mean of Cell Sizes** | 52.19593 |

| Note: | Cell sizes are not equal. |
| --- | --- |

| **Means with the same letter are not significantly different.** | | | |
| --- | --- | --- | --- |
| **t Grouping** | **Mean** | **N** | **Diplo** |
| A | 40.026 | 48 | pc2_pc2 |
|  |  |  |  |
| B | 7.612 | 104 | Pc2_pc2 |
| B |  |  |  |
| B | 1.645 | 37 | Pc2_Pc2 |

| The SAS System |
| --- |

The UNIVARIATE Procedure

Variable: resid

| **Moments** | | | |
| --- | --- | --- | --- |
| **N** | 189 | **Sum Weights** | 189 |
| **Mean** | 0 | **Sum Observations** | 0 |
| **Std Deviation** | 19.3161894 | **Variance** | 373.115172 |
| **Skewness** | 1.3463679 | **Kurtosis** | 3.86892482 |
| **Uncorrected SS** | 70145.6523 | **Corrected SS** | 70145.6523 |
| **Coeff Variation** | . | **Std Error Mean** | 1.40504603 |

| **Basic Statistical Measures** | | | |
| --- | --- | --- | --- |
| **Location** | | **Variability** | |
| **Mean** | 0.00000 | **Std Deviation** | 19.31619 |
| **Median** | -1.64476 | **Variance** | 373.11517 |
| **Mode** | -7.61221 | **Range** | 121.62500 |
|  |  | **Interquartile Range** | 12.01136 |

| **Tests for Location: Mu0=0** | | | | |
| --- | --- | --- | --- | --- |
| **Test** | **Statistic** | | **p Value** | |
| **Student's t** | **t** | 0 | **Pr > \|t\|** | 1.0000 |
| **Sign** | **M** | -34.5 | **Pr >= \|M\|** | <.0001 |
| **Signed Rank** | **S** | -1939.5 | **Pr >= \|S\|** | 0.0095 |

| **Tests for Normality** | | | | |
| --- | --- | --- | --- | --- |
| **Test** | **Statistic** | | **p Value** | |
| **Shapiro-Wilk** | **W** | 0.841252 | **Pr < W** | <0.0001 |
| **Kolmogorov-Smirnov** | **D** | 0.203902 | **Pr > D** | <0.0100 |
| **Cramer-von Mises** | **W-Sq** | 2.186417 | **Pr > W-Sq** | <0.0050 |
| **Anderson-Darling** | **A-Sq** | 11.1525 | **Pr > A-Sq** | <0.0050 |

| **Quantiles (Definition 5)** | |
| --- | --- |
| **Level** | **Quantile** |
| **100% Max** | 81.59935 |
| **99%** | 64.62435 |
| **95%** | 43.11879 |
| **90%** | 22.13779 |
| **75% Q3** | 4.39916 |
| **50% Median** | -1.64476 |
| **25% Q1** | -7.61221 |
| **10%** | -19.90065 |
| **5%** | -30.22565 |
| **1%** | -40.02565 |
| **0% Min** | -40.02565 |

| **Extreme Observations** | | | |
| --- | --- | --- | --- |
| **Lowest** | | **Highest** | |
| **Value** | **Obs** | **Value** | **Obs** |
| -40.0257 | 179 | 62.3493 | 175 |
| -40.0257 | 170 | 63.2243 | 149 |
| -40.0257 | 167 | 63.5743 | 169 |
| -40.0257 | 146 | 64.6243 | 188 |
| -39.1507 | 186 | 81.5993 | 180 |

The UNIVARIATE Procedure


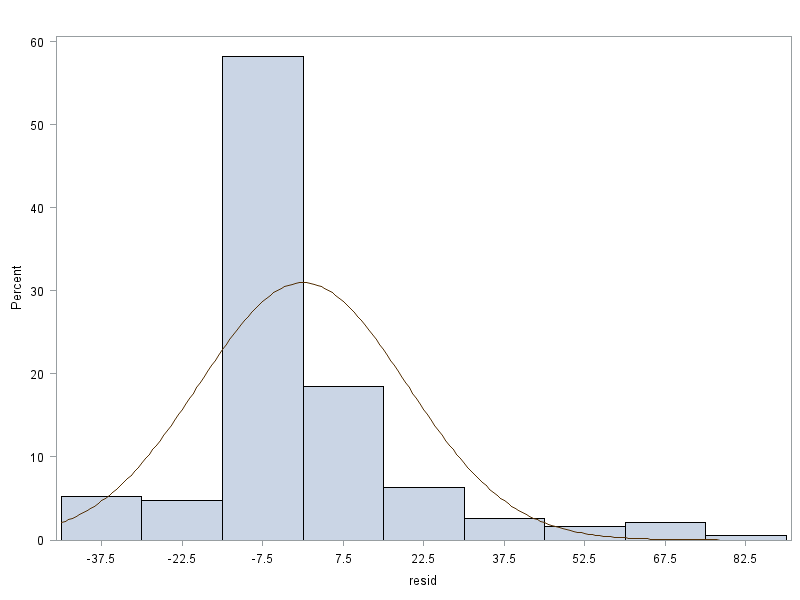


| The SAS System |
| --- |

The UNIVARIATE Procedure

Fitted Normal Distribution for resid

| **Parameters for Normal Distribution** | | |
| --- | --- | --- |
| **Parameter** | **Symbol** | **Estimate** |
| **Mean** | Mu | 0 |
| **Std Dev** | Sigma | 19.31619 |

| **Goodness-of-Fit Tests for Normal Distribution** | | | | |
| --- | --- | --- | --- | --- |
| **Test** | **Statistic** | | **p Value** | |
| **Kolmogorov-Smirnov** | **D** | 0.2039022 | **Pr > D** | <0.010 |
| **Cramer-von Mises** | **W-Sq** | 2.1864165 | **Pr > W-Sq** | <0.005 |
| **Anderson-Darling** | **A-Sq** | 11.1525035 | **Pr > A-Sq** | <0.005 |

| **Quantiles for Normal Distribution** | | |
| --- | --- | --- |
| **Percent** | **Quantile** | |
|  | **Observed** | **Estimated** |
| **1.0** | -40.02565 | -44.9362 |
| **5.0** | -30.22565 | -31.7723 |
| **10.0** | -19.90065 | -24.7547 |
| **25.0** | -7.61221 | -13.0286 |
| **50.0** | -1.64476 | 0.0000 |
| **75.0** | 4.39916 | 13.0286 |
| **90.0** | 22.13779 | 24.7547 |
| **95.0** | 43.11879 | 31.7723 |
| **99.0** | 64.62435 | 44.9362 |
