# Supplementary material for: Pedigree-Based Analysis in a Multiparental Population of Octoploid Strawberry Reveals QTL Alleles Conferring Resistance to Phytophthora cactorum
Source: G3 (Bethesda). 2017 Jun 5;7(6):1707–19. doi: 10.1534/g3.117.042119 (PMC5473751; doi:10.1534/g3.117.042119)
Supplement: Supplementary file 18 [file 1707FileS7.zip › File S7/2 SAS-analysis/QTL-genotype effect analysis/output/2014-15 Discovery - QTL-genotype analysis SAS results.docx]

| The SAS System |
| --- |

The GLM Procedure

| **Class Level Information** | | |
| --- | --- | --- |
| **Class** | **Levels** | **Values** |
| **ID** | 497 | 14.11-11 14.11-29 14.11-36 14.11-4 14.11-41 14.11-49 14.11-66 14.11-99 14.12-1 14.12-11 14.12-12 14.12-19 14.12-22 14.12-25 14.12-26 14.12-34 14.12-41 14.12-8 14.13-16 14.13-38 14.13-4 14.13-49 14.13-57 14.13-62 14.13-65 14.13-70 14.14-1 14.14-2 14.14-3 14.14-7 14.14-9 14.15-1 14.15-11 14.15-21 14.15-45 14.15-47 14.15-57 14.16-13 14.16-21 14.16-23 14.16-24 14.16-26 14.16-35 14.16-36 14.16-37 14.16-42 14.16-9 14.17-1 14.17-30 14.17-41 14.17-56 14.17-67 14.17-78 14.17-83 14.17-88 14.17-90 14.17-97 14.18-10 14.18-15 14.18-18 14.18-25 14.18-30 14.18-31 14.18-33 14.18-36 14.18-9 14.19-10 14.19-12 14.19-38 14.19-51 14.19-57 14.19-61 14.19-71 14.19-79 14.19-88 14.22-13 14.22-15 14.22-17 14.22-18 14.22-19 14.22-20 14.22-5 14.22-6 14.22-7 14.22-8 14.23-12 14.23-14 14.23-17 14.23-18 14.23-22 14.23-5 14.23-52 14.23-53 14.23-57 14.25-1 14.25-17 14.25-4 14.25-41 14.25-5 14.27-54 14.27-61 14.27-63 14.27-67 14.27-72 14.27-77 14.27-78 14.27-82 14.27-84 14.28-1 14.28-14 14.28-2 14.28-20 14.28-21 14.28-3 14.28-4 14.28-5 14.28-8 14.28-9 14.31-13 14.31-14 14.31-16 14.31-21 14.31-24 14.31-27 14.31-34 14.31-4 14.31-6 14.31-8 14.33-1 14.33-10 14.33-15 14.33-17 14.33-18 14.33-19 14.33-2 14.33-22 14.33-4 14.33-7 14.34-11 14.34-13 14.34-18 14.34-21 14.34-29 14.34-39 14.34-40 14.34-5 14.34-7 14.35-1 14.35-11 14.35-2 14.35-53 14.35-54 14.35-76 14.35-78 14.35-89 14.35-98 14.37-2 14.37-22 14.37-23 14.37-27 14.37-34 14.37-48 14.37-64 14.37-72 14.38-24 14.38-25 14.38-36 14.38-42 14.38-5 14.38-65 14.38-73 14.38-82 14.38-83 14.38-92 14.39-14 14.39-15 14.39-19 14.39-2 14.39-26 14.39-30 14.39-31 14.39-35 14.39-4 14.41-5 14.41-57 14.44-13 14.44-20 14.44-27 14.44-28 14.44-30 14.44-35 14.44-36 14.44-42 14.45-13 14.45-19 14.45-29 14.45-36 14.45-39 14.45-4 14.45-58 14.45-9 14.47-2 14.47-22 14.47-24 14.47-36 14.47-44 14.47-55 14.47-90 14.47-94 14.48-19 14.48-25 14.48-28 14.48-29 14.48-35 14.48-4 14.48-43 14.48-47 14.48-6 14.48-8 14.50-10 14.50-11 14.50-12 14.50-67 14.50-71 14.50-78 14.50-82 14.50-83 14.50-84 14.50-92 14.51-1 14.51-12 14.51-19 14.51-2 14.51-23 14.51-3 14.51-33 14.51-37 14.51-40 14.51-6 14.52-12 14.52-13 14.52-14 14.52-17 14.52-21 14.52-30 14.52-38 14.52-60 14.52-89 14.53-1 14.53-10 14.53-12 14.53-13 14.53-18 14.53-20 14.53-24 14.53-29 14.53-31 14.53-9 14.55-12 14.55-15 14.55-2 14.55-25 14.55-3 14.55-8 14.56-18 14.56-22 14.56-4 14.56-44 14.56-64 14.56-71 14.56-73 14.56-74 14.56-76 14.57-1 14.57-17 14.57-26 14.57-27 14.57-3 14.57-44 14.57-6 14.58-1 14.58-13 14.58-2 14.58-20 14.58-21 14.58-28 14.58-29 14.58-3 14.58-4 14.58-9 14.6-104 14.6-12 14.6-22 14.6-4 14.6-6 14.6-74 14.6-82 14.6-83 14.6-85 14.6-88 14.62-16 14.62-20 14.62-43 14.62-55 14.62-8 14.65-38 14.65-49 14.65-66 14.65-76 14.67-1 14.67-10 14.67-11 14.67-15 14.67-19 14.67-2 14.67-23 14.67-24 14.67-32 14.67-5 14.7-100 14.7-110 14.7-113 14.7-114 14.7-123 14.7-126 14.7-86 14.7-92 14.7-94 14.7-95 14.72-1 14.72-16 14.72-24 14.72-46 14.72-52 14.72-62 14.74-10 14.74-12 14.74-13 14.74-16 14.74-21 14.74-22 14.74-4 14.74-6 14.74-7 14.74-9 14.75-29 14.75-33 14.75-63 14.75-69 14.75-84 14.75-85 14.75-87 14.75-94 14.75-98 14.76-11 14.76-12 14.76-13 14.76-23 14.76-26 14.76-30 14.76-35 14.76-5 14.76-7 14.76-8 14.77-2 14.77-72 14.77-87 14.78-10 14.78-15 14.78-29 14.78-3 14.78-30 14.78-36 14.78-45 14.78-47 14.78-50 14.81-10 14.81-21 14.81-25 14.81-3 14.81-32 14.81-60 14.81-63 14.81-65 14.81-73 14.81-9 14.82-10 14.82-14 14.82-23 14.82-25 14.82-28 14.82-31 14.82-52 14.82-59 14.82-7 14.82-8 14.83-14 14.83-18 14.83-49 14.83-5 14.83-8 14.84-1 14.84-11 14.84-2 14.84-27 14.84-29 14.84-3 14.84-31 14.84-33 14.84-58 14.84-7 14.85-16 14.85-18 14.85-46 14.85-48 14.85-9 14.86-21 14.86-22 14.86-23 14.86-33 14.86-39 14.86-42 14.86-48 14.86-5 14.86-57 14.87-18 14.87-21 14.87-22 14.87-28 14.87-29 14.87-37 14.87-40 14.87-52 14.87-8 14.88-14 14.88-22 14.88-44 14.88-9 14.89-13 14.89-19 14.89-33 14.89-5 14.89-9 14.9-102 14.9-106 14.9-116 14.9-34 14.9-38 14.9-47 14.9-55 14.9-63 14.9-77 14.9-9 14.90-10 14.90-21 14.90-27 14.90-35 14.90-57 14.90-60 14.90-79 14.90-85 14.90-9 14.91-20 14.91-25 14.91-28 14.91-33 14.91-38 14.91-4 14.91-42 14.91-44 14.92-11 14.92-22 14.92-23 14.92-3 14.92-31 14.92-45 14.92-65 14.92-73 14.92-74 14.92-84 14.93-10 14.93-11 14.93-12 14.93-39 14.93-61 14.93-67 14.93-77 14.93-78 14.94-1 14.94-12 14.94-16 14.94-3 14.94-31 14.94-33 14.94-42 14.94-63 14.94-72 |
| **Female** | 24 | 11.107-5 11.108-5 11.13-54 11.139-1 11.21-6 11.28-34 11.31-54 11.34-47 11.58-72 11.71-9 11.77-96 11.83-20 11.85-58 11.98-41 FL_08-10 FL_09-46 FL_10-12 FL_10-15 FL_10-16 FL_10-24 FL_10-46 FL_10-89 FL_10-97 Florida1 |
| **Male** | 29 | 11.107-2 11.107-5 11.108-5 11.116-5 11.121-1 11.13-54 11.28-34 11.31-14 11.31-54 11.34-47 11.43-15 11.71-9 11.77-96 11.83-20 11.85-58 FL_07-19 FL_08-10 FL_09-46 FL_09-76 FL_10-12 FL_10-14 FL_10-15 FL_10-16 FL_10-24 FL_10-46 FL_10-89 FL_10-92 FL_10-97 Florida1 |
| **Alle1** | 2 | Pc2 pc2 |
| **Alle2** | 2 | Pc2 pc2 |
| **Diplo** | 3 | Pc2_Pc2 Pc2_pc2 pc2_pc2 |
| **AUDPC** | 87 | 0 7 35 42 49 56 63 70 77 79 84 91 127 3.5 10.5 17.5 24.5 31.5 38.5 45.5 61.5 68.5 73.5 80.5 87.5 94.5 101.5 108.5 115.5 61.25 64.75 1.166666667 103.8333333 11.66666667 110.8333333 113.1666667 12.83333333 120.1666667 15.16666667 16.33333333 19.83333333 22.16666667 23.33333333 26.83333333 29.16666667 30.33333333 32.66666667 36.16666667 37.33333333 39.66666667 40.83333333 43.16666667 44.33333333 46.66666667 47.66666667 47.83333333 48.66666667 5.833333333 50.16666667 51.33333333 53.66666667 54.83333333 56.83333333 57.16666667 58.33333333 60.66666667 64.16666667 65.33333333 67.66666667 68.83333333 72.33333333 74.66666667 79.33333333 8.166666667 81.33333333 81.66666667 82.83333333 85.16666667 87.16666667 89.83333333 90.66666667 90.83333333 92.16666667 95.33333333 96.33333333 96.83333333 97.33333333 |
| **Outl** | 1 | 0 |

| **Number of Observations Read** | 498 |
| --- | --- |
| **Number of Observations Used** | 498 |

| The SAS System |
| --- |

The GLM Procedure

Dependent Variable: AUDPC

| **Source** | **DF** | **Sum of Squares** | **Mean Square** | **F Value** | **Pr > F** |
| --- | --- | --- | --- | --- | --- |
| **Model** | 2 | 111172.9578 | 55586.4789 | 77.09 | <.0001 |
| **Error** | 495 | 356921.1588 | 721.0528 |  |  |
| **Corrected Total** | 497 | 468094.1166 |  |  |  |

| **R-Square** | **Coeff Var** | **Root MSE** | **AUDPC Mean** |
| --- | --- | --- | --- |
| 0.237501 | 116.9249 | 26.85243 | 22.96553 |

| **Source** | **DF** | **Type I SS** | **Mean Square** | **F Value** | **Pr > F** |
| --- | --- | --- | --- | --- | --- |
| **Diplo** | 2 | 111172.9578 | 55586.4789 | 77.09 | <.0001 |

| **Source** | **DF** | **Type III SS** | **Mean Square** | **F Value** | **Pr > F** |
| --- | --- | --- | --- | --- | --- |
| **Diplo** | 2 | 111172.9578 | 55586.4789 | 77.09 | <.0001 |


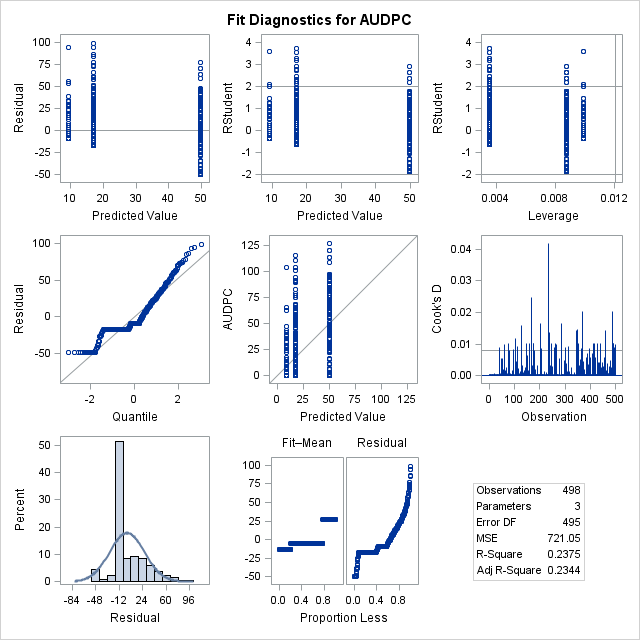


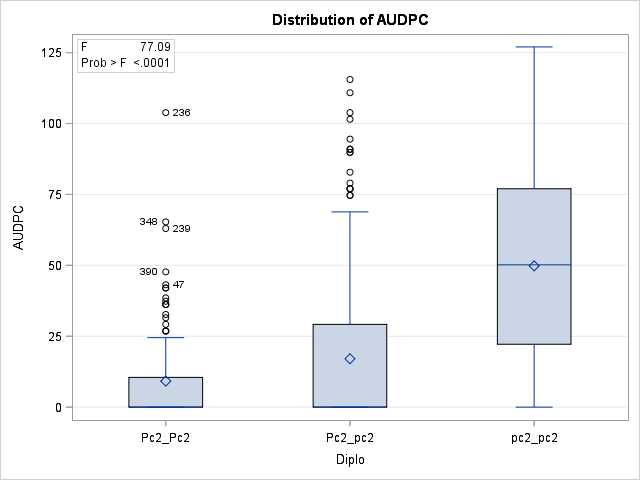


| The SAS System |
| --- |

The GLM Procedure


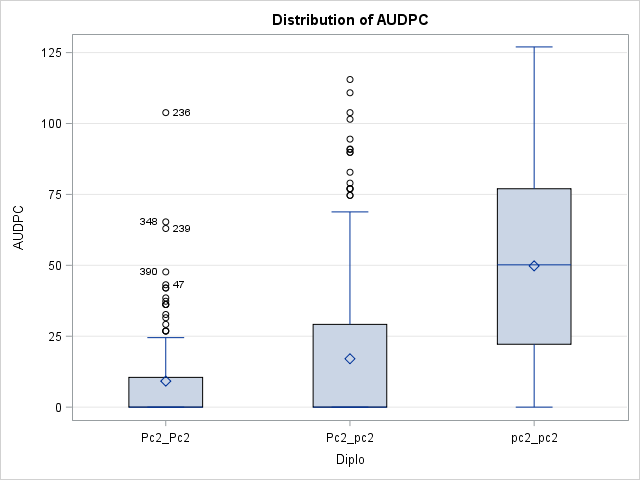


| The SAS System |
| --- |

The GLM Procedure

t Tests (LSD) for AUDPC

| Note: | This test controls the Type I comparisonwise error rate, not the experimentwise error rate. |
| --- | --- |

| **Alpha** | 0.05 |
| --- | --- |
| **Error Degrees of Freedom** | 495 |
| **Error Mean Square** | 721.0528 |
| **Critical Value of t** | 1.96477 |
| **Least Significant Difference** | 6.4193 |
| **Harmonic Mean of Cell Sizes** | 135.0956 |

| Note: | Cell sizes are not equal. |
| --- | --- |

| **Means with the same letter are not significantly different.** | | | |
| --- | --- | --- | --- |
| **t Grouping** | **Mean** | **N** | **Diplo** |
| A | 49.810 | 114 | pc2_pc2 |
|  |  |  |  |
| B | 17.071 | 283 | Pc2_pc2 |
|  |  |  |  |
| C | 9.182 | 101 | Pc2_Pc2 |

| The SAS System |
| --- |

The UNIVARIATE Procedure

Variable: resid

| **Moments** | | | |
| --- | --- | --- | --- |
| **N** | 498 | **Sum Weights** | 498 |
| **Mean** | 0 | **Sum Observations** | 0 |
| **Std Deviation** | 26.7983437 | **Variance** | 718.151225 |
| **Skewness** | 1.01852497 | **Kurtosis** | 1.15785114 |
| **Uncorrected SS** | 356921.159 | **Corrected SS** | 356921.159 |
| **Coeff Variation** | . | **Std Error Mean** | 1.2008625 |

| **Basic Statistical Measures** | | | |
| --- | --- | --- | --- |
| **Location** | | **Variability** | |
| **Mean** | 0.0000 | **Std Deviation** | 26.79834 |
| **Median** | -9.1815 | **Variance** | 718.15122 |
| **Mode** | -17.0713 | **Range** | 148.23868 |
|  |  | **Interquartile Range** | 31.50000 |

| **Tests for Location: Mu0=0** | | | | |
| --- | --- | --- | --- | --- |
| **Test** | **Statistic** | | **p Value** | |
| **Student's t** | **t** | 0 | **Pr > \|t\|** | 1.0000 |
| **Sign** | **M** | -67 | **Pr >= \|M\|** | <.0001 |
| **Signed Rank** | **S** | -6701.5 | **Pr >= \|S\|** | 0.0361 |

| **Tests for Normality** | | | | |
| --- | --- | --- | --- | --- |
| **Test** | **Statistic** | | **p Value** | |
| **Shapiro-Wilk** | **W** | 0.887981 | **Pr < W** | <0.0001 |
| **Kolmogorov-Smirnov** | **D** | 0.210483 | **Pr > D** | <0.0100 |
| **Cramer-von Mises** | **W-Sq** | 4.561255 | **Pr > W-Sq** | <0.0050 |
| **Anderson-Darling** | **A-Sq** | 23.26596 | **Pr > A-Sq** | <0.0050 |

| **Quantiles (Definition 5)** | |
| --- | --- |
| **Level** | **Quantile** |
| **100% Max** | 98.42874 |
| **99%** | 84.42874 |
| **95%** | 53.81848 |
| **90%** | 38.48515 |
| **75% Q3** | 14.42874 |
| **50% Median** | -9.18152 |
| **25% Q1** | -17.07126 |
| **10%** | -17.07126 |
| **5%** | -38.14327 |
| **1%** | -49.80994 |
| **0% Min** | -49.80994 |

| **Extreme Observations** | | | |
| --- | --- | --- | --- |
| **Lowest** | | **Highest** | |
| **Value** | **Obs** | **Value** | **Obs** |
| -49.8099 | 493 | 84.4287 | 104 |
| -49.8099 | 490 | 86.7621 | 234 |
| -49.8099 | 440 | 93.7621 | 346 |
| -49.8099 | 430 | 94.6518 | 236 |
| -49.8099 | 415 | 98.4287 | 126 |

The UNIVARIATE Procedure


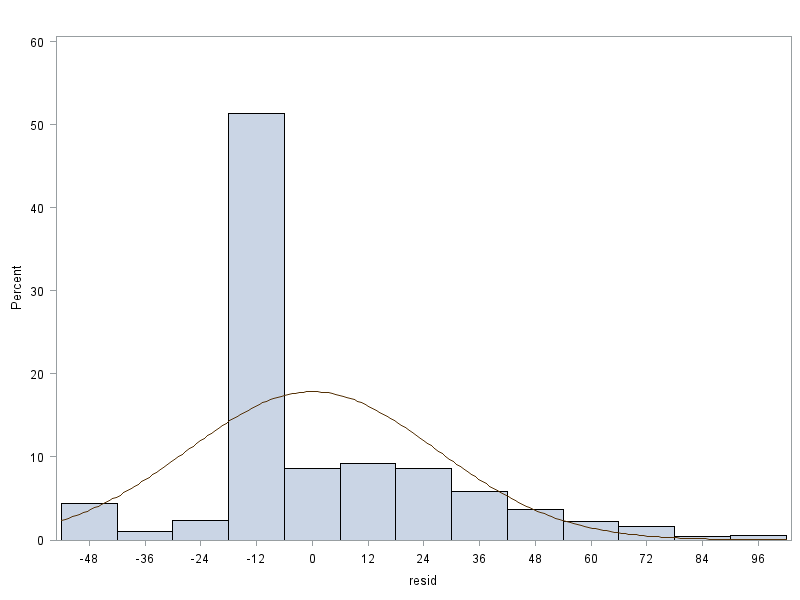


| The SAS System |
| --- |

The UNIVARIATE Procedure

Fitted Normal Distribution for resid

| **Parameters for Normal Distribution** | | |
| --- | --- | --- |
| **Parameter** | **Symbol** | **Estimate** |
| **Mean** | Mu | 0 |
| **Std Dev** | Sigma | 26.79834 |

| **Goodness-of-Fit Tests for Normal Distribution** | | | | |
| --- | --- | --- | --- | --- |
| **Test** | **Statistic** | | **p Value** | |
| **Kolmogorov-Smirnov** | **D** | 0.2104829 | **Pr > D** | <0.010 |
| **Cramer-von Mises** | **W-Sq** | 4.5612553 | **Pr > W-Sq** | <0.005 |
| **Anderson-Darling** | **A-Sq** | 23.2659573 | **Pr > A-Sq** | <0.005 |

| **Quantiles for Normal Distribution** | | |
| --- | --- | --- |
| **Percent** | **Quantile** | |
|  | **Observed** | **Estimated** |
| **1.0** | -49.80994 | -62.3423 |
| **5.0** | -38.14327 | -44.0794 |
| **10.0** | -17.07126 | -34.3435 |
| **25.0** | -17.07126 | -18.0752 |
| **50.0** | -9.18152 | 0.0000 |
| **75.0** | 14.42874 | 18.0752 |
| **90.0** | 38.48515 | 34.3435 |
| **95.0** | 53.81848 | 44.0794 |
| **99.0** | 84.42874 | 62.3423 |
