# Supplementary material for: Pedigree-Based Analysis in a Multiparental Population of Octoploid Strawberry Reveals QTL Alleles Conferring Resistance to Phytophthora cactorum
Source: G3 (Bethesda). 2017 Jun 5;7(6):1707–19. doi: 10.1534/g3.117.042119 (PMC5473751; doi:10.1534/g3.117.042119)
Supplement: Supplementary file 18 [file 1707FileS7.zip › File S7/2 SAS-analysis/QTL-genotype effect analysis/output/2014-15 Validation - QTL-genotype analysis SAS results.docx]

| The SAS System |
| --- |

The GLM Procedure

| **Class Level Information** | | |
| --- | --- | --- |
| **Class** | **Levels** | **Values** |
| **ID** | 218 | 11.107-24 11.107-5 11.116-56 11.121-19 11.13-54 11.28-34 11.31-54 11.34-47 11.71-9 11.83-20 11.85-58 11.98-41 12.74-39 13.1-77 13.10-32 13.12-146 13.12-148 13.13-179 13.13-93 13.14-14 13.14-152 13.14-18 13.14-69 13.19-137 13.19-143 13.19-86 13.20-109 13.20-138 13.20-149 13.20-70 13.21-173 13.22-121 13.22-158 13.22-191 13.22-200 13.22-222 13.22-32 13.22-327 13.22-330 13.22-336 13.22-397 13.22-403 13.22-407 13.22-412 13.22-445 13.22-46 13.22-499 13.22-51 13.22-549 13.22-566 13.22-62 13.22-64 13.23-106 13.23-107 13.23-32 13.23-48 13.24-148 13.24-63 13.25-289 13.25-35 13.26-124 13.26-126 13.26-131 13.26-162 13.26-90 13.27-142 13.28-24 13.28-6 13.29-61 13.29-66 13.30-173 13.30-225 13.32-13 13.34-102 13.34-95 13.35-105 13.37-127 13.37-144 13.37-173 13.37-203 13.37-212 13.37-399 13.38-141 13.38-185 13.38-46 13.4-119 13.4-122 13.4-130 13.4-135 13.4-151 13.4-191 13.4-31 13.4-53 13.4-56 13.4-79 13.4-83 13.4-86 13.41-108 13.41-181 13.41-67 13.41-7 13.41-75 13.41-8 13.42-1 13.42-113 13.42-171 13.42-257 13.42-37 13.42-5 13.42-78 13.44-60 13.44-77 13.44-82 13.45-10 13.45-106 13.45-13 13.45-211 13.45-73 13.46-137 13.46-178 13.46-88 13.46-98 13.47-140 13.47-145 13.47-177 13.47-183 13.47-188 13.47-210 13.47-232 13.47-58 13.48-205 13.48-242 13.48-252 13.5-61 13.50-124 13.50-214 13.50-226 13.50-258 13.51-103 13.51-134 13.51-135 13.51-154 13.51-157 13.51-162 13.51-186 13.51-207 13.51-35 13.51-64 13.52-19 13.52-65 13.53-98 13.54-165 13.54-171 13.55-124 13.55-126 13.55-127 13.55-154 13.55-169 13.55-178 13.55-179 13.55-195 13.55-20 13.56-1 13.56-211 13.56-222 13.56-33 13.56-83 13.58-17 13.58-40 13.58-5 13.58-8 13.6-127 13.6-199 13.6-234 13.60-98 13.61-70 13.61-92 13.63-60 13.64-133 13.64-157 13.64-7 13.65-107 13.65-140 13.65-143 13.65-157 13.65-159 13.65-16 13.65-177 13.65-180 13.65-89 13.66-45 13.7-18 13.7-186 13.7-78 13.70-12 13.70-17 13.8-54 13.8-66 13.8-88 13.9-59 FL_06-134 FL_06-38 FL_06-89 FL_07-102 FL_07-193 FL_09-134 FL_09-46 FL_10-153 FL_10-24 FL_10-47 FL_10-51 FL_10-64 FL_10-89 FL_10-94 FL_10-97 Festival Florida12 Wintersta |
| **Female** | 36 | - 11.85-58 AU_2006-47 FL_06-134 FL_06-38 FL_06-45 FL_06-89 FL_07-102 FL_07-193 FL_08-10 FL_08-78 FL_09-134 FL_09-46 FL_09-57 FL_10-153 FL_10-166 FL_10-24 FL_10-47 FL_10-51 FL_10-89 FL_10-94 FL_10-97 F_11.121-1 F_11.28 F_11.34 F_13.10-32 F_13.24 F_13.48-24 F_13.51-15 F_13.6-126 F_13.70 F_FL_10-47 F_FL_10-89 Florida127 WinterDawn Winterstar |
| **Male** | 43 | - 11.107-5 11.116-68 11.121-19 11.28-34 11.31-36 11.63-41 11.98-41 AU_2004-2 FL_02-58 FL_05-131 FL_06-134 FL_06-89 FL_07-102 FL_07-168 FL_07-193 FL_07-68 FL_08-10 FL_08-50 FL_09-110 FL_09-134 FL_09-46 FL_09-57 FL_10-15 FL_10-153 FL_10-19 FL_10-24 FL_10-47 FL_10-51 FL_10-64 FL_10-94 FL_10-97 Florida12 M_11.13 M_11.28 M_13.13 M_13.13-9 M_13.14-1 M_13.35 M_13.38-1 M_13.38-4 M_FL_10-6 Wintersta |
| **Alle1** | 2 | Pc2 pc2 |
| **Alle2** | 2 | Pc2 pc2 |
| **Diplo** | 3 | Pc2_Pc2 Pc2_pc2 pc2_pc2 |
| **AUDPC** | 190 | 0 1.51612662 120.365474 13.0619043 14.9007959 16.0369043 16.6507959 18.4007959 21.4507959 27.6757959 28.3222935 3.35306992 3.96612662 32.4007959 33.0472935 46.3570459 53.0507959 65.6507959 74.6632959 95.9257959 0.200795899 0.238335615 0.376081348 0.402041475 0.436403253 0.507175807 0.530301506 0.550131668 0.611403253 0.679249085 1.018964978 1.173185136 1.243137973 1.492177193 1.511904305 1.738167907 1.775131668 1.866416483 1.929818867 10.01084522 10.54835373 10.79141648 103.7764713 109.3531234 11.05108135 11.09676859 11.78833561 11.80806744 11.86085373 11.91813797 111.2881679 12.39569765 13.17704148 13.37176859 13.46085706 13.47981887 13.52704148 13.70204148 14.00500228 14.05989726 14.08640325 14.11612662 14.20108135 14.55013167 15.06813797 15.85204109 16.45481887 16.62981887 16.65013167 16.68833561 17.89552579 19.10108135 19.16140325 2.058067444 2.125131668 2.293137973 2.714317444 2.825131668 2.871768587 22.28833561 23.31889117 23.36140325 23.47513167 23.64625469 23.80481887 24.34313797 24.73489726 25.38030151 26.26813797 26.51731887 26.97513167 27.00357097 27.20941468 27.59153099 28.78412923 28.89112662 28.92857097 29.05481887 29.63833561 3.446968173 3.913335615 30.45530151 30.83412653 32.05108135 32.21431744 33.80013167 34.54026739 35.02513167 36.56055784 37.78555784 38.50481887 38.61065748 39.75013167 39.78833561 4.141530988 4.555301506 4.750795899 4.901817444 4.986403253 41.15108135 41.41641648 41.71333561 41.83030151 41.90835762 43.20501551 43.56316791 43.76439942 44.06918706 44.28030151 46.81180706 47.28259703 48.14313797 48.30481887 5.016530988 5.035853731 5.063167907 5.315623411 5.450795899 5.451081348 5.604818867 5.975795899 5.976081348 50.69818514 51.47513167 51.82513167 52.39026739 53.31612662 54.07981887 54.31333561 55.08516648 57.06813797 59.46085373 59.56526739 6.304818867 6.363335615 6.540267386 6.643708142 6.668137973 6.785853731 6.888335615 6.960853731 66.37704148 66.68030151 66.73833561 67.00250469 68.77445995 7.588335615 7.704818867 7.900131668 72.87752759 73.83816791 75.13833561 8.243137973 8.251081348 8.426081348 8.465267386 8.990267386 86.19668937 87.00108135 87.98585373 88.78833561 89.19112662 9.081381668 9.279818867 9.300795899 9.825795899 93.93306744 94.58931744 96.83833561 96.93816791 |
| **Outl** | 2 | 0 1 |

| **Number of Observations Read** | 218 |
| --- | --- |
| **Number of Observations Used** | 218 |

| The SAS System |
| --- |

The GLM Procedure

Dependent Variable: AUDPC

| **Source** | **DF** | **Sum of Squares** | **Mean Square** | **F Value** | **Pr > F** |
| --- | --- | --- | --- | --- | --- |
| **Model** | 2 | 51046.1291 | 25523.0646 | 49.44 | <.0001 |
| **Error** | 215 | 110997.2663 | 516.2664 |  |  |
| **Corrected Total** | 217 | 162043.3954 |  |  |  |

| **R-Square** | **Coeff Var** | **Root MSE** | **AUDPC Mean** |
| --- | --- | --- | --- |
| 0.315015 | 93.31114 | 22.72150 | 24.35025 |

| **Source** | **DF** | **Type I SS** | **Mean Square** | **F Value** | **Pr > F** |
| --- | --- | --- | --- | --- | --- |
| **Diplo** | 2 | 51046.12912 | 25523.06456 | 49.44 | <.0001 |

| **Source** | **DF** | **Type III SS** | **Mean Square** | **F Value** | **Pr > F** |
| --- | --- | --- | --- | --- | --- |
| **Diplo** | 2 | 51046.12912 | 25523.06456 | 49.44 | <.0001 |


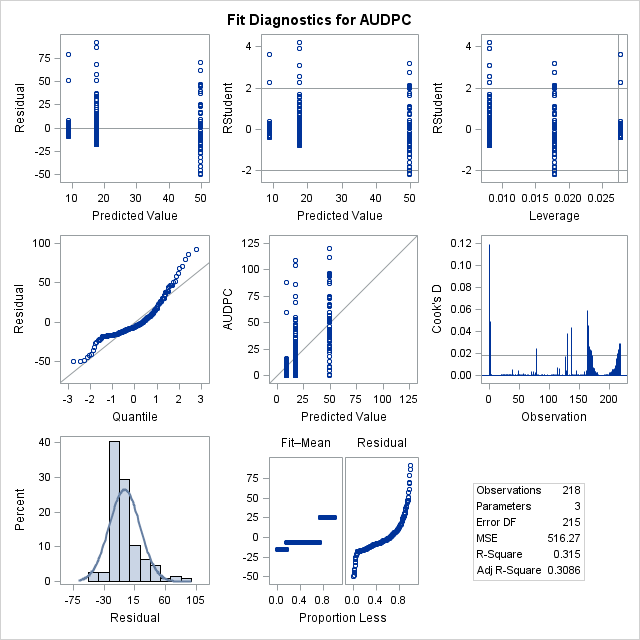


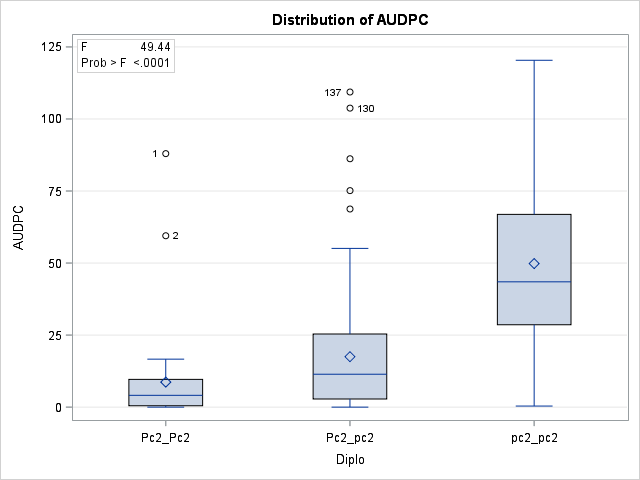


| The SAS System |
| --- |

The GLM Procedure


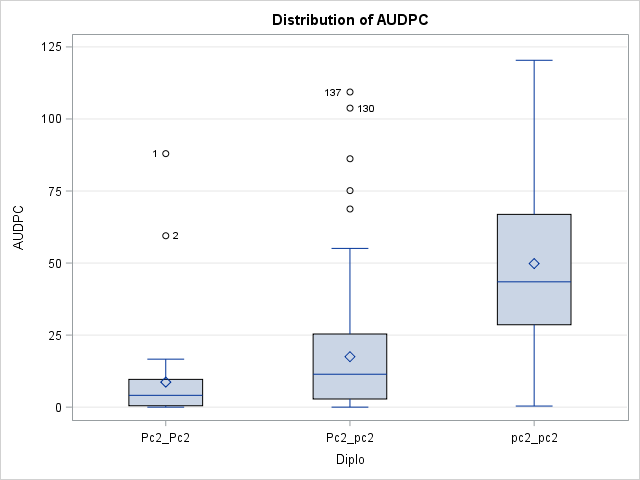


| The SAS System |
| --- |

The GLM Procedure

t Tests (LSD) for AUDPC

| Note: | This test controls the Type I comparisonwise error rate, not the experimentwise error rate. |
| --- | --- |

| **Alpha** | 0.05 |
| --- | --- |
| **Error Degrees of Freedom** | 215 |
| **Error Mean Square** | 516.2664 |
| **Critical Value of t** | 1.97106 |
| **Least Significant Difference** | 8.4636 |
| **Harmonic Mean of Cell Sizes** | 56 |

| Note: | Cell sizes are not equal. |
| --- | --- |

| **Means with the same letter are not significantly different.** | | | |
| --- | --- | --- | --- |
| **t Grouping** | **Mean** | **N** | **Diplo** |
| A | 49.816 | 56 | pc2_pc2 |
|  |  |  |  |
| B | 17.506 | 126 | Pc2_pc2 |
|  |  |  |  |
| C | 8.691 | 36 | Pc2_Pc2 |

| The SAS System |
| --- |

The UNIVARIATE Procedure

Variable: resid

| **Moments** | | | |
| --- | --- | --- | --- |
| **N** | 218 | **Sum Weights** | 218 |
| **Mean** | 0 | **Sum Observations** | 0 |
| **Std Deviation** | 22.6165457 | **Variance** | 511.508139 |
| **Skewness** | 1.35871585 | **Kurtosis** | 2.91012134 |
| **Uncorrected SS** | 110997.266 | **Corrected SS** | 110997.266 |
| **Coeff Variation** | . | **Std Error Mean** | 1.53178576 |

| **Basic Statistical Measures** | | | |
| --- | --- | --- | --- |
| **Location** | | **Variability** | |
| **Mean** | 0.0000 | **Std Deviation** | 22.61655 |
| **Median** | -5.8848 | **Variance** | 511.50814 |
| **Mode** | -17.5063 | **Range** | 141.28679 |
|  |  | **Interquartile Range** | 20.84506 |

| **Tests for Location: Mu0=0** | | | | |
| --- | --- | --- | --- | --- |
| **Test** | **Statistic** | | **p Value** | |
| **Student's t** | **t** | 0 | **Pr > \|t\|** | 1.0000 |
| **Sign** | **M** | -32 | **Pr >= \|M\|** | <.0001 |
| **Signed Rank** | **S** | -2251.5 | **Pr >= \|S\|** | 0.0154 |

| **Tests for Normality** | | | | |
| --- | --- | --- | --- | --- |
| **Test** | **Statistic** | | **p Value** | |
| **Shapiro-Wilk** | **W** | 0.88206 | **Pr < W** | <0.0001 |
| **Kolmogorov-Smirnov** | **D** | 0.15204 | **Pr > D** | <0.0100 |
| **Cramer-von Mises** | **W-Sq** | 1.61519 | **Pr > W-Sq** | <0.0050 |
| **Anderson-Darling** | **A-Sq** | 8.729107 | **Pr > A-Sq** | <0.0050 |

| **Quantiles (Definition 5)** | |
| --- | --- |
| **Level** | **Quantile** |
| **100% Max** | 91.84684 |
| **99%** | 79.29516 |
| **95%** | 47.02231 |
| **90%** | 30.63685 |
| **75% Q3** | 7.25211 |
| **50% Median** | -5.88479 |
| **25% Q1** | -13.59295 |
| **10%** | -17.50628 |
| **5%** | -22.81246 |
| **1%** | -48.57289 |
| **0% Min** | -49.43995 |

| **Extreme Observations** | | | |
| --- | --- | --- | --- |
| **Lowest** | | **Highest** | |
| **Value** | **Obs** | **Value** | **Obs** |
| -49.4399 | 218 | 68.6904 | 78 |
| -49.2659 | 217 | 70.5494 | 163 |
| -48.5729 | 216 | 79.2952 | 1 |
| -44.9142 | 215 | 86.2702 | 130 |
| -41.5649 | 214 | 91.8468 | 137 |

The UNIVARIATE Procedure


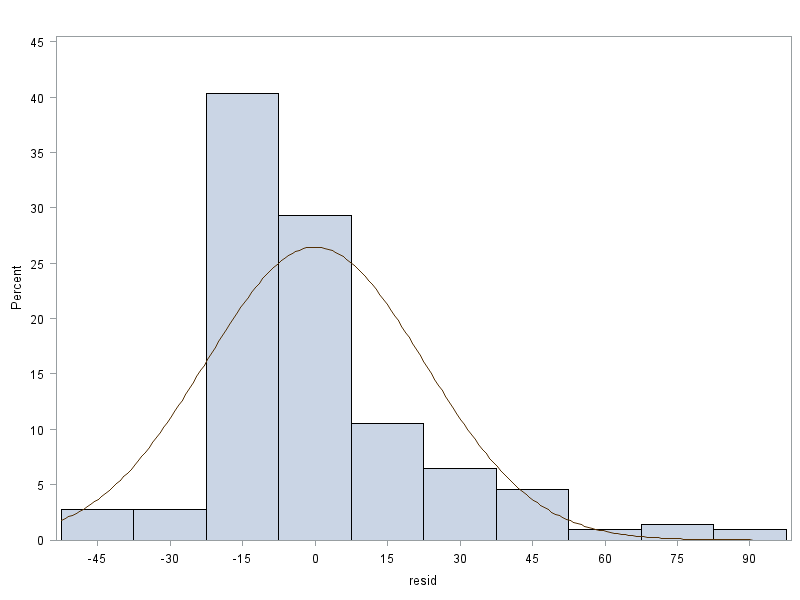


| The SAS System |
| --- |

The UNIVARIATE Procedure

Fitted Normal Distribution for resid

| **Parameters for Normal Distribution** | | |
| --- | --- | --- |
| **Parameter** | **Symbol** | **Estimate** |
| **Mean** | Mu | 0 |
| **Std Dev** | Sigma | 22.61655 |

| **Goodness-of-Fit Tests for Normal Distribution** | | | | |
| --- | --- | --- | --- | --- |
| **Test** | **Statistic** | | **p Value** | |
| **Kolmogorov-Smirnov** | **D** | 0.15203965 | **Pr > D** | <0.010 |
| **Cramer-von Mises** | **W-Sq** | 1.61518994 | **Pr > W-Sq** | <0.005 |
| **Anderson-Darling** | **A-Sq** | 8.72910682 | **Pr > A-Sq** | <0.005 |

| **Quantiles for Normal Distribution** | | |
| --- | --- | --- |
| **Percent** | **Quantile** | |
|  | **Observed** | **Estimated** |
| **1.0** | -48.57289 | -52.6140 |
| **5.0** | -22.81246 | -37.2009 |
| **10.0** | -17.50628 | -28.9843 |
| **25.0** | -13.59295 | -15.2546 |
| **50.0** | -5.88479 | 0.0000 |
| **75.0** | 7.25211 | 15.2546 |
| **90.0** | 30.63685 | 28.9843 |
| **95.0** | 47.02231 | 37.2009 |
| **99.0** | 79.29516 | 52.6140 |
