# Supplementary material for: Pedigree-Based Analysis in a Multiparental Population of Octoploid Strawberry Reveals QTL Alleles Conferring Resistance to Phytophthora cactorum
Source: G3 (Bethesda). 2017 Jun 5;7(6):1707–19. doi: 10.1534/g3.117.042119 (PMC5473751; doi:10.1534/g3.117.042119)
Supplement: Supplementary file 18 [file 1707FileS7.zip › File S7/Whitaker_Pedigree_results_README.docx]

Dear Editorial Board members,

Followings are descriptions of result folders and files that we have been sked to submit.

Under the folder “Results” there are three sub folders:

1. “**Software**”- under the “software” sub folder there are two folders, one for “Genome wide QTL analysis” and another for “Linkage group 7D specific QTL analysis”.
   1. Under folder “Genome wide QTL analysis”: 2013-14 genome wide QTL analysis (rep 1, 2, 3) and 2014-15 genome wide QTL analysis (rep 1, 2, 3).
   2. There are three folders under each rep: “input”, “output”, and “setting”. For example, under “**input**” folder there are 7 files, “Geno….txt” is data file which contains SNP calls, pedigree and phenotypic data. “FQmap3.8K_2.txt” contain genetic map for 3,799 SNP probes and their genomic locations. ”flexqtl.PAR” file is parameter file that contains all software settings used to execute FlexQTL™ analysis. “flexqtl.LIC” is license file. “flexqtl_job.txt” is batch file used to execute LINUX version of FlexQTL™. “flexqtl099130” and “postqtl099130” are LINUX version of FlexQTL™ software.
   3. Under the folder “Linkage group 7D specific QTL analysis”, there are two folders: “Pc 2013-14-LG7D” and “Pc 2014-15 – LG7D”, which contains 3 reps of FlexQTL™ analysis inputs, outputs, and settings, as detailed above.
   4. Under “**output**” folder, there are two files “Flex.log” and “mhaplotype.csv” files. Flex.log file contains relevant results at the bottom and “mhaplotype.csv” file contains phased marker haplotypes.
   5. Under “settings” folder, there is only one file called “flexqtl.PAR” (also present on input folder) containing all parameter settings to execute the analysis.
2. “**SAS-analysis**” - Under SAS analysis folder, there are two folders: (1) “Diplotype effect analysis”, and (2) “QTL-genotype effect analysis”, each containing two sub folders called “input” and “output”. “Input” folder contains SAS input files and “Output” folder contains SAS output results.
3. “**Production trait analysis**” – This folder contains two files containing inputs and outputs of analysis of QTL-genotype vs. production traits in validation sets from 2013 and 2014.
