# Supplementary material for: Pedigree-Based Analysis in a Multiparental Population of Octoploid Strawberry Reveals QTL Alleles Conferring Resistance to Phytophthora cactorum
Source: G3 (Bethesda). 2017 Jun 5;7(6):1707–19. doi: 10.1534/g3.117.042119 (PMC5473751; doi:10.1534/g3.117.042119)
Supplement: Supplementary file 20 [file 1707TableS1.pdf]

Supplementary Table S1 Parameters for QTL analyses with FlexQTL™ software, values used for analyses in the present study and justifications for values chosen

FlexQTL™ parameters	Parameter description	Value	Value description
**indiN**	Indicator for nuisance variable	3	All indicated individuals will have genetic information displayed in output
We desired output for all individuals, as there were no subsets of individuals for which we did not want outputs or for which a separate type of analysis was conducted.			
**nchrom**	Number of chromosomes in the data file	37	Number of chromosomes used for genome-wide runs
Whole-genome analyses were conducted. Since 9 linkage groups were split into 2 around homozygous regions, this made for a total of 28 +9 =37 linkage groups for QTL analysis.			
**indiC**	Indicator of chromosomes to be included in the QTL analysis	0 or 1	0=excluded, 1=included
All 37 linkage groups were included in each genome-wide analysis.			
**nmrkrC**	Number of markers per linkage group as in data file and map file	n	Varies across chromosomes
For example, linkage group 1A had 316 polymorphic SNP markers, n = 316.			
**indicQTL**	Indicator for QTL OR IBD analysis	1	1 = QTL analysis<br>2 = IBD analysis
FlexQTL™ software conducts either a QTL or an IBD (Identity-By-Descent) analysis. We performed a QTL analysis.			
**maximQTL**	Maximum number of QTL to be included in the model	15	Up to 15 QTL allowed in each run
This is a random value based on the presumed complexity of the trait under analysis. Based on phenotypic distributions of mortality, we did not expect more than 5 QTLs influencing mortality due to *P. cactorum*. We typically choose to identify and further analyze QTLs only when they have a certain level of statistical evidence (2lnBF>5), whereas FlexQTL™ considers regions with a lower thresholds (2lnBF>2). For this reason, the maximum number is usually doubled. The number is then further increased such that the prior distributions for the number of QTL frequently allow the testing of ~10 QTLs. This maximum number could have been reduced after the initial runs showed only one highly significant QTL.			
**priorQTL**	Prior distribution for the number of QTL, the variable being the mean of a (truncated) Poisson distribution	1 or 3	One or three QTL assumed across replicate runs
FlexQTL™ is based on Bayesian statistics that require priors. The minimum value is 1. Outcomes of Bayesian			

analyses might vary with priors. Therefore, it is common practice to test the robustness of results for different settings. In the present study, without previous information, we chose “1” and “3” as priors for replicate runs. The same QTL was detected in both cases, increasing confidence in the results.			
**addGENE**	Indicator for additive genetic effects for individual QTL	5	Additive genetic effects with normal prior distribution
FlexQTL™ is capable of simulating additive and dominance genetic models. For each of these two genetic models there were six options with unique identifiers from 0-5, where 0 = model excluded from analysis. We chose to run additive genetic models with normal prior distributions and random (Co) variance matrix diagonals (5) because this is the standard in previous literature (Bink et al. 2012, 2014; Roach et al. 2016).			
**domGENE**	Indicator for dominant genetic effects for individual QTL	0	Dominance model excluded
We chose the additive over the dominance model for the final replicate runs. We did run preliminary dominance models which showed no differences in results from additive models and were thus not continued.			
**length**	Length of Markov chain	100,000	100,000 QTL simulations
FlexQTL™ simulates Markov chain Monte Carlo (MCMC) iterations. For statistical inference, the sampling of 1000 models is common in the literature (e.g. Bink et al. 2012, 2014). Applying a thinning of 100 (see below), a chain length of 100,000 was needed to obtain 1000 independent samples.			
**Thinning**	Thinning of Markov chain with respect to writing samples to file	100	With a chain length of 100,000 this writes 100,000/100 or 1000 models (samples) to an output file
Thinning is needed to allow the sampling of simulations that can be considered as independent from each other. The size depends on the complexity of the trait and data. When results show convergence, the thinning was adequate from a statistical point of view. Subsequently, lower values might be explored to reduce chain sizes and thereby reduce computation time.			
**Ess**	Effective sample size (ess) , a convergence measure variable to obtain a sufficient number of samples for statistical inference (100 minimum)	101	Minim effective sample size of 100 required for convergence
Defining ess=101 will guarantee an effective chain size of > 100 samples for the estimation of the mean (mean1), variance of errors (VERR11) , number of QTLs (nQTL), and variance for the number of QTLs (VQTL11). A non-zero value of the variable will supersede the value of the length of the Markov chain, i.e. the chain will be prolonged until the ESS criteria has not been reached with a maximum of five times the defined length. The minimum threshold of 100 has been applied on all previously published papers with FlexQTL™.			
**markerblock**	Number of marker loci within a block that are jointly updated with respect to linkage phase among marker (and	5	5 markers per block

<table>
<tr><td></td><td>QTL) alleles</td><td></td><td></td></tr>
<tr><td colspan="4">FlexQTL™ guidelines advise a minimum block size of 2 (especially in case of IBD estimation) and a maximum block size of 5 (may be increased further for high density maps but sampling will require more computational time). We agreed that 5 was appropriate for the level of LD in our germplasm and the density of our map.</td></tr>
<tr><td>skipSampleMarkers</td><td>Indicator variable to skip sampling of marker information in Markov chain</td><td>100</td><td>In order to obtain independent samples for statistical inference, every 100th sample of 100,000 was sampled</td></tr>
<tr><td colspan="4">Indicator variable that defines how frequently the linkage phase of markers will be re-estimated. With dense, high quality SNP data, this value could be 5% of the chain length for a QTL analysis. As marker density varied considerably with our dataset, we preferred a conservative value. When the aim is to accurately define SNP haplotypes rather than perform a QTL analyses, this variable should be zero.</td></tr>
<tr><td>distanceQTLs</td><td>Minimal distance between any pair of two QTLs</td><td>0.0</td><td>Did not restrict distance</td></tr>
<tr><td colspan="4">We prefered 0.0 because we wanted to explore all possibilities. Higher values might not have allowed us to detect QTLs in close proximity to one another. The only other option is 0.1 which indicates 0.1 M or 10 cM.</td></tr>
<tr><td>MSegDelta</td><td>Neutralizer for extreme segregation distortion for marker loci during the marker consistency check preceeding the actual QTL analysis</td><td>1</td><td>0 = Extreme segregation distortion is not accepted
1 = Extreme distortion is accepted</td></tr>
<tr><td colspan="4">This indicator variable defines whether extreme segregation distortion (e.g. lack of a genotype class in a segregating progeny) should be rejected or accepted. Such a distortion could be due to genotyping errors or have a biological basis, such as gametic incompatibility or selection against disease susceptibility. When distortion is not accepted, FlexQTL™ will adjust the parental genotype calls according to the genotype calls of the progeny.</td></tr>
<tr><td>DeleteDR</td><td>Indicator variable for deleting ALL observed singletons</td><td>1</td><td>0 = All marker calls that cause singletons are maintained
1 = Calls are deleted</td></tr>
<tr><td colspan="4">Double recombinations around a single marker should be extremely rare with high-density SNP data. When present, these are mostly due to calling errors rather than to true double recombination. We chose to delete marker calls causing double recombinants, replacing the call with the symbol for missing values.</td></tr>
<tr><td>Seed</td><td>A random integer value to start the simulation process and to also define the number of QTL to be included in the initial model of the MCMC simulation</td><td>variable</td><td>variable</td></tr>
<tr><td colspan="4">Large integer value that is used in random number generating algorithms of the MCMC simulation process.</td></tr>
</table>

For example these can be random numbers from uniform, normal, and inverted gamma distributions. With FlexQTL™ the last digit of the seed determines the number of QTL in the initial model of the Markov chain: with 0-4 the initial model has no QTL and with 5-9 the initial model has the maximum number indicated by 'maximQTL'. Replicated runs with different seeds were performed to examine whether results were robust across seeds or whether outcomes varied considerably. Though this number is supposed to be randomly chosen, it is common practice to edit the last digit such that both possible starts are used.
