# Supplementary material for: Pedigree-Based Analysis in a Multiparental Population of Octoploid Strawberry Reveals QTL Alleles Conferring Resistance to Phytophthora cactorum
Source: G3 (Bethesda). 2017 Jun 5;7(6):1707–19. doi: 10.1534/g3.117.042119 (PMC5473751; doi:10.1534/g3.117.042119)
Supplement: Supplementary file 21 [file 1707Whitaker_pedigree_data_policydocumentation.docx]

**Pedigree-based analysis in a multiparental population of octoploid strawberry reveals QTL alleles conferring resistance to Phytophthora cactorum**

Jozer Mangandi, Sujeet Verma, Luis F. Osorio, Natalia A. Peres, Eric van de Weg, and Vance M. Whitaker

**Raw genotype/marker information**

**Data File S1**: Genotypic data from the 2013-14 discovery population set. This file contains 3,799 probes and their SNP calls for 551 seedling individuals and 19 parents. The 2013-14 discovery population individuals’ names start with “13.” Each row from row #3 represents a probeset ID and each column from column D represents an individual.

**Data File S2**: Genotypic data from the 2013-14 validation set. This file contains 3,799 probes and their SNP calls for 252 advanced selections and cultivars. Each row from row #3 represents a probeset ID and each column from column D represents an individual.

**Data File S3**: Genotypic data from the 2014-15 discovery population set. This file contains 3,799 probes and their SNP calls for 576 seedling individuals and 40 parents. The 2014-15 discovery population individuals’ names start with “14.” Each row from row #3 represents a probeset ID and each column from column D represents an individual.

**Data File S4**: Genotypic data from the 2014-15 validation set. This file contains 3,799 probes and their SNP calls for 273 advanced selections and cultivars. Each row from row #3 represents a probeset ID and each column from column D represents an individual.

**Data File S6**: Physical and genetic locations for SNP probes. This file contains physical locations for 3,799 SNP probes based on the *Fragaria* *vesca* ‘Hawaii 4’ v1.0 and *Fragaria* *vesca* ssp. *bracteata* v2.0 (Tennessen et al. 2014) reference genomes. Genetic locations are provided for linkage group 7D and should be sufficient for recapitulating the analysis. A separate manuscript is in preparation for publication in 2017 that details the entire reference genetic map, which the map in the present study closely resembles and upon which it is based. We are concerned that the public availability of all map positions at this time will cause problems for the reference map publication. The reference map will be the most dense and most highly curated map for octoploid strawberry to date, and the publication in preparation will provide all marker calls and JoinMap input files.

**Raw phenotype data**

**Data File S5**: Phenotypic data from all four trials. Mortality due to Phytophthora crown rot is expressed as the mean area under disease progress curve (AUDPC) for each individual. Immediate parents and diplotypes are also included for each individual.

**Results files**

**Data File S7**: Zip folder containing results from software outputs. The folder also contains a word file “Whitaker_Pedigree_results_README” explaining the outputs in further detail.

**Software**

**Data File S7**: Zip folder containing results from software outputs. The folder also contains a word file “Whitaker_Pedigree_results_README” explaining the outputs in further detail.
